# Llama Single Domain Antibodies Specific for the 7 Botulinum Neurotoxin Serotypes as Heptaplex Immunoreagents

**DOI:** 10.1371/journal.pone.0008818

**Published:** 2010-01-21

**Authors:** Jerry O. Conway, Laura J. Sherwood, M. Thelma Collazo, John A. Garza, Andrew Hayhurst

**Affiliations:** Department of Virology and Immunology, Southwest Foundation for Biomedical Research, San Antonio, Texas, United States of America; Griffith University, Australia

## Abstract

**Background:**

There are currently 7 known serotypes of botulinum neurotoxin (BoNT) classified upon non-cross reactivity of neutralizing immunoglobulins. Non-neutralizing immunoglobulins, however, can exhibit cross-reactivities between 2 or more serotypes, particularly mosaic forms, which can hamper the development of highly specific immunoassays, especially if based on polyclonal antisera. Here we employ facile recombinant antibody technology to subtractively select ligands to each of the 7 BoNT serotypes, resulting in populations with very high specificity for their intended serotype.

**Methods and Findings:**

A single llama was immunized with a cocktail of 7 BoNT toxoids to generate a phage display library of single domain antibodies (sdAb, VHH or nanobodies) which were selected on live toxins. Resulting sdAb were capable of detecting both toxin and toxin complex with the best combinations able to detect 100s-10s of pg per 50 µL sample in a liquid bead array. The most sensitive sdAb were combined in a heptaplex assay to identify each of the BoNT serotypes in buffer and milk and to a lesser extent in carrot juice, orange juice and cola. Several anti-A(1) sdAb recognized A2 complex, showing that subtype cross-reactivity within a serotype was evident. Many of our sdAb could act as both captor and tracer for several toxin and toxin complexes suggesting sdAb can be used as architectural probes to indicate BoNT oligomerisation. Six of 14 anti-A clones exhibited inhibition of SNAP-25 cleavage in the neuro-2A assay indicating some sdAb had toxin neutralizing capabilities. Many sdAb were also shown to be refoldable after exposure to high temperatures in contrast to polyclonal antisera, as monitored by circular dichroism.

**Conclusions:**

Our panel of molecularly flexible antibodies should not only serve as a good starting point for ruggedizing assays and inhibitors, but enable the intricate architectures of BoNT toxins and complexes to be probed more extensively.

## Introduction

Botulinum neurotoxins (BoNT) are still the most poisonous naturally occurring substances known [1], with extrapolations from non-human primate studies indicating that lethal doses for humans would be 1 µg/kg, 10 ng/kg or 1 pg/kg for oral, inhalation and injection routes respectively [2], [3], [4]. For perspective, it has been estimated that BoNT are 100 billion times more toxic than cyanide [5]. The extreme potency, widespread distribution in soils of producing strains, and relative ease of production has meant that BoNT are the only toxins in the highest risk group i.e., CDC category A, of biological agents thought to pose a potential threat alongside *Marburgvirus* and *Bacillus anthracis* [6], [7]. Indeed, it has been predicted that contamination of centralized milk supplies could result in hundreds of thousands of cases in the absence of suitable detection methods [8] and it has been speculated that, “it is likely only a matter of time until botulism is intentionally caused…” [9]. Since intoxication can result in a paralysis so severe it can require mechanical ventilation for weeks to months, it would be facile to overwhelm health authorities and cause mass casualties *via* BoNT mis-use [9], [10].

Specific species of the spore forming anaerobe *Clostridium* produce BoNT as 150 kDa proteins with one or more neurotoxin accessory proteins (NAPs) to form toxin complexes or progenitors of varying sizes approximately 300, 500, and 900 kDa known as M, L and LL. The NAPs shield the toxin from the harsh protease rich environments of the stomach and intestine, elevating the potency of the ingestion route several hundred fold over toxin and may also play a role in uptake across the intestinal epithelium. The toxins themselves consist of an N-terminal translocation domain (Hn or HCT) and C-terminal receptor binding domain (Hc or HCR) comprising a 100 kDa heavy chain (HC) fragment, which is disulfide linked to a 50 kDa proteolytic light chain domain (LC or Lc). The Hc targets receptors on pre-synaptic membranes at neuromuscular junctions where the toxin is endocytosed and the Hn is subsequently triggered by low pH to translocate the Lc into the cytosol. Lc cleaves specific proteins involved in acetylcholine release to inhibit nerve transmission and cause muscle relaxation (for recent review see [11]).

There are 7 serotypes of BoNT (A, B, C, D, E, F and G) based upon non-cross-reactive neutralizing antisera specific to the 150 kDa toxin component. While A, B, E, and F have been definitively linked to human botulism, G has been implicated in 7 cases of sudden unexpected deaths [12], [13], and C, D are typically associated with farming/wildlife outbreaks. Importantly, all 7 serotypes have been shown to be highly lethal in non-human primate models [3]. Neutralizing sera are not necessarily absolutely specific since cross-neutralization can occur between E and A [14], B, F [15]. A non-protective antibody cross-reactive with B, C, D and E has been isolated [16] and polyclonal antibodies raised to fragments of A have been shown to cross react with heterologous holotoxins [17]. Furthermore, serotypes D and C have high Lc and Hn homologies, which can confer cross-reactivity to antibodies [18], [19] and some C/D and D/C toxins are “mosaics” with low homology to either parent in the Hc domain [20]. Several serotypes have subtypes (currently A = 5, C = 2, D = 2, E = 3 and F = 4) that can show reduced reactivity towards antibodies to the major subtype [21], [22], [23]. Indeed, sequence divergence of the BoNT is substantial considering they share many similarities in a complex series of functions [24]. For example, each serotype has a unique protease cleavage site specificity within the SNARE complex, though often in shared targets with A and E recognizing SNAP-25, B, D, F and G recognizing VAMP and C recognizing both SNAP-25 and syntaxin. The cleavage specificities of various subtypes are under study with remarkable differences in cleavage rates among recombinant Lc of A1-A4 already being observed using synthetic SNAPtide substrate [25].

The NAPs tend to be less conserved across serotypes, though homologies have been identified from recent genomic studies [26], [27] and cross-reactivities have been noted from immunological studies [28], [29], [30]. Within those subtypes so far studied, NAPS can also vary, with prominent components such as HA33 of subtype A1 being present in A4 but not A2 or A3 for example [27]. Thus, the addition of NAPs to toxin varies according to serotype and potentially subtype: A exists in M, L and LL forms; B/C/D/E in L and M forms; F in M form; and G in L form. *C. botulinum* normally produces one of A, B, C, D, E or F and *C. argentinense* produces G. *C. butyricum* and *C. baratii* also produce E and F, respectively, yet the E has been shown to be partially immunologically distinct from the botulinum E counterpart [31] and the F is even more divergent [32] and expected to be sero-distinguishable. Some B and all E strains are non-proteolytic and so the toxin is a single chain molecule that may have different surface topography to the component chains once nicked and activated as shown for serotype E [33]. To complicate matters more, bivalent strains of *C. botulinum* exist, which can produce two different serotypes (Ab, Af, Ba, or Bf) where the capital letter indicates the prominent serotype [15]. Finally, the NAPs have been shown to occlude the recognition of toxin by particular antibodies [34], though exposure to high pH can reduce this steric hindrance [35], [36]. As can be seen, immunoassay development for BoNT and their complexes is extremely challenging!

We are interested in developing disruptive antibody technologies to probe and counter high consequence targets, and one aspect of this employs llama single domain antibodies (sdAb or nanobodies) to generate rugged ligands [30], [37], [38]. SdAb are derived by cloning the variable domains of the heavy chain only antibody of Camelids or IgNAR of sharks and expressing them recombinantly (see [39], [40], [41] for reviews). SdAb are highly soluble, well expressed, and have been shown to refold after thermal or chemical denaturation unlike conventional multi-domain immunoglobulins or their recombinant derivatives [42], [43], [44], [45]. At approximately 1/10^th^ the size of an IgG, their compact architecture is proving advantageous in accessing cryptic epitopes normally out of bounds to standard immunoglobulins [46], [47] potentially offering new routes of neutralization. SdAb also appear highly capable of crystallizing and co-crystallizing with their target antigens to enable high resolution structures of even fickle targets to be obtained [48], [49].

With all of these advantages in hand, we reasoned that sdAb would make ideal candidates to begin probing the complex antigenic make-up of the BoNT molecules and offer potentially novel inhibitory routes. Therefore, we chose to generate sdAb to all 7 BoNT serotypes and examine their specificities and sensitivities on both toxins and toxin complexes. We also examined the *in vitro* neutralizing ability and the molecular flexibility of some of the sdAb obtained.

## Results and Discussion

### Generating Anti-BoNT sdAb

**Figure 1:**
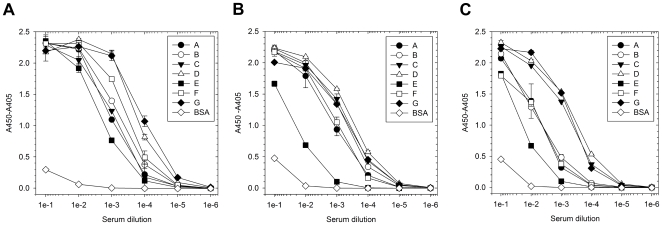
Seroconversion of Snoop the llama after being immunized with the 7 serotypes of BoNT toxoids. Antibody capture from serum after the 6^th^ immunization was monitored on the seven serotypes of a) toxoid, b) toxin and c) toxin complexes versus a control antigen, bovine serum albumin (BSA).

We immunized a single llama with a cocktail of all 7 serotypes of the toxins as toxoids and observed seroconversion against toxoids, toxins and toxin complexes by ELISA (Figure 1). We used 6 immunizations yet did not see appreciable increase in seroconversion between doses 4 and 6, suggesting the response had plateaued (data not shown). After the final immunization, we cloned the variable heavy chain repertoire into a phage display vector using variable heavy domain framework 1 and 4 specific primers to capture VHH and VH genes since the latter have been shown to produce sdAb with favorable biophysical characteristics too [50]. The library was approximately 1e+9 clones with 24/24 unique clones having inserts at a ratio of almost 2 VH: 1VHH as judged by examining the amino acid composition of framework 2 [51].

**Figure 2:**
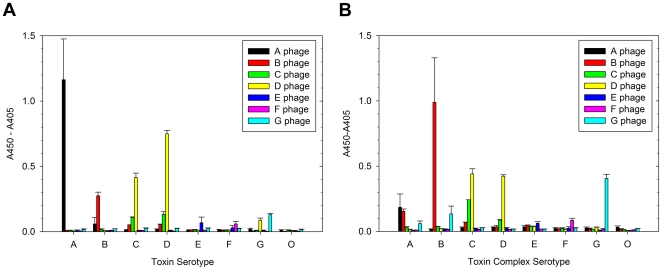
Capturing the llama anti-BoNT repertoire by phage display. Polyclonal phage ELISA of round 2 selected phage populations analyzed on each a) toxin or b) toxin complex indicates the potential degree of specificity for the serotype of toxin upon which the population was selected.

The library was mined for BoNT binders by selections on biotinylated toxins in the presence of excess non-biotinylated decoy toxins, resulting in the isolation of polyclonal phage mostly specific for the serotype upon which they were selected except for those selected on C (C1) and D (Figure 2). Serotypes C and D share regions of homology, particularly in the Hn domain [20], implying that some sdAb selected on D or C could be cross-reactive. Furthermore, the D serotype supplied by Metabiologics is actually a D/C mosaic, and while it shares high Hn homology and partial Lc homology, it differs from both D and C parental serotypes in its Hc [20].

The polyclonal phage populations were deconvoluted by monoclonal phage ELISA on the serotype of toxin upon which they were selected and positive clones sequenced to reveal several unique predicted amino acid sequences for each serotype A(18), B(21), C(26), D(36), E(8), F(10) and G(27) (Figure S1). It is noteworthy that only 10 of 146 unique sequences were VH (A14, A15, B21, D4, D5, D12, D13, D19, D20 and G24) suggesting that there may actually be a counter-selection against these domains perhaps due to the lack of affinity contributions by VL. However, the dominance of VH over VHH has recently been shown for cancer marker antigens [52], indicating that the target requirement for VL contributions and/or other factors are at play. Despite the cross-reactivity between C and D, we could not identify any clones that were common to both populations.

### Characterization of Anti-BoNT sdAb Serotype Specificity

We managed to subclone, express and purify over 100 different sdAb genes A(18), B(19), C(22), D(28), E(8), F(9) and G(22) for analysis. The purified sdAb proteins were incorporated into bead suspension microarrays (Luminex). Each antibody was coupled to a unique bead region so that the mix of captors could be analyzed against each biotinylated tracer individually for specificity to be gauged and a first indication of sensitivity to be noted. To choose appropriate clones, we used a concentration of 1e+5 pg BoNT per well, which did not saturate our ligands and so resulted in sensitivity discrimination. First, we chose an upper limit of approximately 500 median fluorescent intensities (MFI) from this data to rule out relatively insensitive combinations that would not have the dynamic range we sought (ideally several thousand MFI). Second, we calculated the percentage cross-reactivity of each pair on mixes of decoy toxins and toxin complexes (Tables S1).

Almost all sdAb were capable of highly specific recognition of the serotype upon which they were selected apart from some of the C and most of the D populations, presumably owing to the homologies previously discussed. Typically, for each pair of sdAb captor and tracer, the mean percentage cross-reactivity on a mix of non-cognate or decoy serotypes was below 0.1%. High resolution of target serotype indicates that, apart from when mosaic toxins are encountered, subtractive panning on live holotoxins is capable of delivering ligands with the desired specificity. The anti-C clones that did have high reactivities on non-cognate mixes were further examined to confirm that it was D (D/C mosaic) reactivity rather than broader cross-reactivity (data not shown). We therefore did not pursue anti-C clones that showed high cross-reactivities further. Since the E proteins we used were based upon non-activated forms we expect our clones to be able to detect naturally occurring forms and further studies will be needed to determine if they are capable of recognizing the dichain molecules.

All sdAb were capable of binding both toxin and toxin complex suggesting that 1) either the sdAb targeted epitopes on the toxin not shielded by the complex of NAP proteins as seen with some scFv [34], or 2) the toxin complexes were disassembled and were not shielding the toxin effectively. We found it odd that *none* of our sdAb to any serotype appeared to be occluded by the complex proteins and it would be tempting to speculate that the small size of the sdAb may enable them to penetrate the NAP shield and target the toxin beneath. However, it is important to note that our PBSTB assay buffer is pH 7.3, which may encourage the dissociation of the complexes which is favored by alkaline pH 7.5–8 [35], [36], [53], [54]. However, as noted below, the range of sdAb able to act as both tracer and captor varied between toxin and complex, indicating that toxin epitopes were not identically presented, inferring that complex dissociation was not occurring or was incomplete.

Many sdAb could perform as both captor and tracer, thereby hinting at the oligomeric potential of some toxins and their complexes. It has been found previously that at pH 7.0 toxin A can be detected as a dimer, trimer and higher species, B as a dimer, and E as a dimer and monomer [55]. We noticed sdAb clones B2, B3, B5, B7, B17 and B18 were capable of detecting toxin when used alone and also capable of detecting complex alone, as were B4, B6, B10, B13, B14 and B20. Only C6 could detect toxin alone, yet C3, C4, C6, C7, C8, C22 and C25 were able to detect complex. D22, E8, F5 and F9 were also single-handedly able to detect their respective toxins and toxin complexes. Of the anti-G clones, only G11, when used alone, was able to detect toxin, while G2, G3, G4, G11 and G18 were all able to detect complex. Therefore, it is possible that B, C, D, E, F, G toxin complexes, and perhaps C, F and G toxins also form dimers or higher structures, though cross-linking and other biophysical analyses will naturally be needed to confirm these suspicions.

The pairs with the lowest cross-reactivity and therefore the highest specificity are presented in Table 1 with the values obtained using rabbit polyclonal sera as controls to demonstrate improved specificities of sdAb. Our sdAb are between 775 fold (B complex) and 48 fold (C toxin) less cross-reactive than the corresponding rabbit polyclonal sera.

**Table 1:** Comparison of cross-reactivities of the chosen sdAb pairs specific for each serotype and rabbit polyclonal antibodies when challenged with 1e+5 pg of toxins or toxin complexes.

Antigen	Captor	Tracer	Cognate mMFI^1^	Non-cognate mMFI^2^	% Cross-reactivity
A toxin	A18	A17	7349±77	2.25±0.25	0.03
B toxin	B4	B2	1871±247	1.25±0.75	0.07
C toxin	C1	C24	7009±61	8.0±2.0	0.11
D toxin	D22	D16	1393±48	32.25±0.25	2.32
E toxin	E7	E4	3052±58	2.75±0.75	0.09
F toxin	F9	F5	7032±77	4.5±0.5	0.06
G toxin	G20	G3	5008±197	1.5±0.5	0.05
A toxin	A Rab	A Rab	910±40	111±8.5	12.1
B toxin	B Rab	B Rab	2226±34	125±4.75	5.6
C toxin	C Rab	C Rab	1453±71	77±2	5.3
E toxin	E Rab	E Rab	388±41	47±1.75	12.2
F toxin	F Rab	F Rab	415±27	42±2	10.1
A complex	A18	A17	4091±102	2.0±0	0.05
B complex	B4	B2	11390±130	2.0±0	0.02
C complex	C1	C24	3766±358	3.25±0.75	0.09
D complex	D22	D16	1343±78	4.0±2	0.3
E complex	E7	E4	2311±126	3.5±0.5	0.15
F complex	F9	F5	5940±113	7.5±0.5	0.13
G complex	G20	G3	5374±357	2.0±0	0.04
A complex	A Rab	A Rab	1690±205	68±8	4.0
B complex	B Rab	B Rab	2655±57	411±23.5	15.5
C complex	C Rab	C Rab	808±40	77±8.5	9.5
E complex	E Rab	E Rab	470±35	53±1.25	11.2
F complex	F Rab	F Rab	247±12	36±5.5	14.4

1 Cognate refers to the serotype on which the sdAb were selected upon or raised against (for the rabbit sera).

2 Non-cognate refers to a mix of all other serotypes at 1e+5 pg each.

1,2 mMFI, mean median fluorescent intensity.

### Characterization of Anti-BoNT sdAb Sensitivity

**Figure 3:**
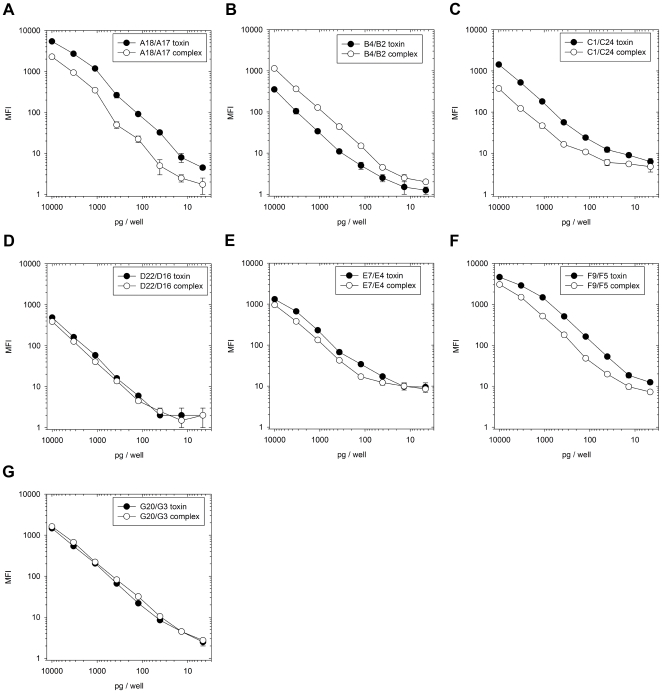
Deducing the sensitivity of selected anti-BoNT llama single domain antibodies. Lower limits of detection of the best antibody pairs on cognate toxin and toxin complex for each serotype a) A, b) B, c) C, d) D, e) E, f) F, and g) G. To provide a non-specific background value for each plot, the mMFI of the pairs employed on 1e+5 pg/well (i.e. 10x the top concentration used in this titration) of non-cognate serotypes are provided as follows: A toxin, 2.3; A complex, 2.0; B toxin, 1.3; B complex, 2.0; C toxin, 8.0; C complex, 3.3; D toxin, 32.3; D complex, 4; E toxin, 2.8; E complex; 3.5; F toxin, 4.5; F complex, 7.5; G toxin, 2.5; G complex, 2.0.

**Figure 4:**
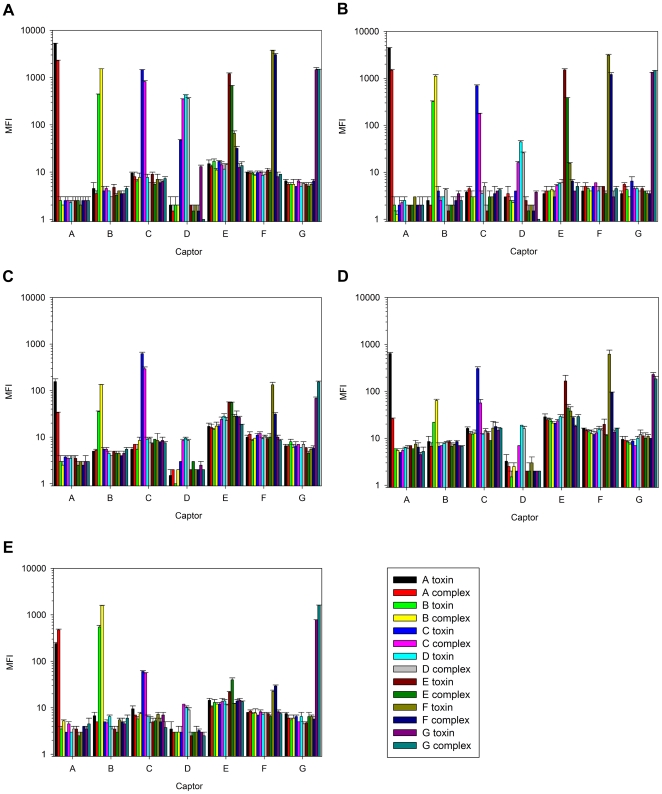
Heptaplex assays using selected pairs of anti-BoNT captors and tracers in various food matrices. Assays were challenged with 1e+4 pg per well of each of the toxin or toxin complexes in a) PBSTB, b) 2% reduced fat milk, c) orange juice d) carrot juice and e) cola.

The best pair of sdAb against each serotype was then employed in a titration of cognate toxin and toxin complex to determine the lower limits of detection (Figure 3). Our threshold was set at the MFI value obtained by multiplying by ten, the MFI yielded on 1e+5 pg mix of non-target serotype. Since there can be batch to batch variation of toxin preparations and a standardized mouse bioassay can be used to normalize active toxin concentrations between them, we also calculated the lower LOD in MLD_50_ based upon activity data kindly provided by Metabiologics. We therefore estimate our lower LOD per 50 µL sample to be approximately 30 pg (0.81 MLD_50_) of A toxin, 100 pg (3.5 MLD_50_) of A complex, 300 pg (33 MLD_50_) of B toxin, 100 pg (0.95 MLD_50_) of B complex, 300 pg (7.2 MLD_50_) of C toxin, 600 pg (4.2 MLD_50_) of C complex, 500 pg (45 MLD_50_) of D toxin, 60 pg (1.74 MLD_50_) of D complex, 80 pg (4.8 MLD_50_) of E toxin, 300 pg (9 MLD_50_) of E complex, 30 pg (0.6 MLD_50_) of F toxin, 200 pg (0.64 MLD_50_) of F complex, 100 pg (1.4 MLD_50_) of G toxin and 70 pg (0.27 MLD_50_) of G complex. A heptaplex assay was established in buffer, milk, orange, carrot juice and cola (Figure 4) revealing that the panel could detect and discriminate all serotypes when present at 1e+4 pg/well in buffer and milk, but to a lesser extent in the other matrices. We relied on microfiltration for our assay and had to centrifuge the food matrices to avoid clogging and did not monitor any losses of toxin or complex in that process nor adherence to the filters themselves. Perhaps magnetic beads would be more reliable for food-stuff analysis to avoid such aggressive sample preparation [56]. Captor D (raised against a D/C mosaic) though relatively specific with tracer D alone (table 1), here appeared to also capture C complex (and C toxin in buffer) perhaps by virtue of tracer C binding. While we have not matched the high sensitivities of and breadth of foodstuffs tested by others [57] the methodology could be improved using directed evolution of the sdAb genes and can also be fully automated on the FDA approved Bioplex 2200. It would certainly be exciting to test the current assay on “real world samples” to give us an idea of whether our chosen clones are indeed the best performers in harsh matrices and crude culture filtrates.

### Characterization of Anti-BoNT sdAb Subtype Specificity

The anti-A (A1) clones showed varying reactivity against A2 complex and we tabulated the A2 cross-reactivities for those combinations already having the highest specificity for A1 (Table 2). While many (n = 10) combinations were highly specific for A1 (0–1% A2/A1 cross-reactivity), most (n = 19) showed marginal (1–10%) cross-reactivities, yet some (n = 3) showed high (80–100%) cross-reactivities. Our most sensitive combination of A18 captor with A17 tracer was still capable of 32% cross-reactivity. Based on extrapolation of data in figure 3a., we would expect a lower limit of detection of approximately 300 pg (21 MLD_50_) of A2 complex and 100 pg of A2 toxin in a 50 µL sample volume if a signal to noise ratio of 10 is used to define a positive. We aim to further explore subtype specificity of anti-A clones and other clones using synthetic gene assembly of BoNT domains since rare subtypes can be fickle to obtain.

**Table 2:** Distribution of A2 cross-reactivities of the most serotype A specific tracer captor pairs.

% cross-reactivity on A2	Pairs with non-cognate cross-reactivity < = 0.1%	Pairs with non-cognate cross-reactivity < = 1%
0–1	2	8
1–10	5	14
10–20	4	4
20–40	5	4
40–60	2	2
60–80	0	2
80–100	0	3

### Inhibitory Effect of sdAb on BoNT Activity

**Figure 5:**
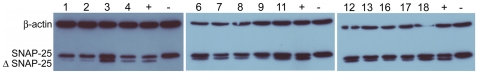
*In vitro* tissue culture assay to discover potential inhibitory activities of our sdAb clones. Neuro-2A assay demonstrating the ability of some of the anti-A BoNT sdAb to inhibit the process of BoNT intoxication by reducing the intracellular cleavage of SNAP-25. + indicates toxin without sdAb and – indicates no toxin and no sdAb.

We employed the neuro-2A assay to preliminarily explore any inhibitory effects of sdAb on BoNT activity. The assay involves combining toxin with antibody and incubating the mix over the neuroblastoma monolayer for several days, after which, the cleavage of the pertinent SNARE target is monitored. We concentrated on our anti-A clones and show that six of the anti-A clones (A1, A2, A8, A9, A16, A17) demonstrate reduced SNAP-25 cleavage by virtue of a decrease in the amount of Δ-SNAP-25 relative to SNAP-25 itself (Figure 5). This assay and others like it are complex and rather fickle since a relatively large amount of toxin is required to elicit modest cleavage which can only be monitored after cell collection, lysis and western blotting. That these remarkable little antibodies 1/10^th^ the size of the toxins can impact intoxication under these conditions certainly indicates more decisive studies are needed. We hope to use more streamlined assays that enable cleavage to be monitored *in vivo* [58] for all of our clones and then deconvolute the sites of inhibition. Since sdAb have been shown to be ideally suited for inhibiting enzymes by intruding into their catalytic pockets [47], exploring this potential with *in vitro* cleavage assays [59] will be especially exciting!

### Molecular Flexibility of the Anti-BoNT sdAb

We examined the refoldability of our best sdAb candidates using circular dichroism (Figure S2, panels a-n) and found they varied in their ability to regain a refolded state with C24, D22, F5, G3 and G21 outperforming the rest (Table 3). Suspecting that molecular heterogeneity may have been responsible for mediochre performance in this assay we re-purified A17 and A18 on a larger scale and subjected the proteins to more stringent chromatography conditions such that by SDS-PAGE they were judged to be >98% pure, yet saw the same CD signatures (data not shown). While we chose the fastest cycling times and did not optimize buffer conditions, nor explore chemical denaturation, it has been noted previously that sdAb can vary widely in their molecular flexibility and ability to refold [60]. Despite this, all but one of our candidates (F9) appeared superior to the polyclonal conventional IgG preparations (Figure S2, panels o-s).

**Table 3:** Percent refoldedness of the most specific and sensitive sdAb clones at each of the two cooling cycles.

sdAb	cool 1	cool 2
A17	105.0	102.3
A18	52.2	32.2
B4	64.0	60.7
B2	70.4	59.3
C1	87.5	80.2
C24	93.8	93.2
D22	96.9	96.0
D16	100.9	97.3
E7	105.5	104.9
E4	89.4	86.7
F9	87.2	89.7
F5	98.9	99.3
G20	94.0	97.5
G3	87.7	85.2

### Conclusions

In this work, our goal was not so much to compete with existing diagnostics and therapeutics for BoNT but rather begin exploring the capacity of a relatively novel type of antibody (sdAb or nanobody) to probe the BoNT architectures for unique epitopes and inhibitory activities. It appears that sdAb are capable of highly specific BoNT recognition, perhaps by virtue of their smaller non-antigen binding surface areas minimizing unwanted cross-reactivities as opposed to larger multi-domain immunoglobulins. Several sdAb were capable of acting as both captor and detector for specific BoNT serotypes, indicating their potential as probes for toxin and toxin complex higher order structures. A handful of anti-A clones were also shown to inhibit the activity of BoNT A in a tissue culture assay and it would be pertinent to determine if the inhibition occurs *via* receptor blocking or at a later stage *via* uptake [61].

We were impressed by the ability of a single llama to deliver a broad range of ligands with good sensitivity and mostly exquisite specificity, after being immunized with relatively low amounts (since they were so costly) of multiple immunogens that have been shown to be far from native [62] and far from optimal in eliciting the highest antibody titers [63], [64]. Our low immune responses and resulting mediochre limits of detection for serotype E and F especially, would indicate that using newer more native toxoided formulations [63], [64], catalytically inactive mutants [65], [66], [67], bead bound forms [56] or recombinant BoNT fragments [14], [18], [68], [69] may deliver a wider diversity of sdAb with higher sensitivities. To our knowledge, these sdAb represent the first recombinant antibodies specific for BoNT serotypes other than A, B or E [70], [71], [72], [73] and we hope these and future improved derivatives will facilitate increased biosecurity. For example, many new and promising detection systems can be super-sensitized with an antibody capture step [74], [75], and these may benefit from non-inhibitory antibodies to less common BoNT serotypes.

It would be difficult to envision these current sdAb as competitors with very promising immunotherapeutics derived from fully human recombinant antibody cocktails [76] that are aimed at clearing toxin appearing in serum prior to uptake by susceptible neurons, since sdAb are likely to be both rapidly cleared without modification [77] and then potentially immunogenic unless humanized. However, since such a countermeasure must be given immediately after exposure, there is great interest in novel approaches to inactivate/eliminate toxin once neuronal uptake has occurred and botulism is fully apparent. Once inside the neuron, toxin is refractory to conventional circulating antibodies though may perhaps be targeted by anti-Lc sdAb fusions as an Hc targeted intrabody [78]. It would also be tempting to speculate that engineered anti-BoNT sdAb might also be one day employed as efficacious oral anti-dotes with further ruggedization to counter the harsh gastric environment [79].

## Materials and Methods

### Materials

All BoNT toxoids, toxins, toxin complexes and anti-BoNT rabbit polyclonal antibodies were from Metabiologics (Madison, WI). The primary production strains used by Metabiologics were A Hall, B Okra, C Brazil, D 5995, E Alaska, F Langeland, G 89, and A2 complex was from FR1 honey isolate. Mouse lethal dose 50% (MLD_50_) per mg values were provided as follows: Toxins: A 2.7e+7, B 1.1e+8, C 2.4e+7, D 1.0e+8, E 6.0e+7, F 2.0e+7, G 1.4e+7; Complexes: A 3.5e+7, 9.5e+6, C 7.0e+6, 2.9e+7, E 3.0e+7, F 3.2e+6, G 3.9e+6, A2 7e+7. Both toxins and complexes were provided at 1 mg/mL.

### Biosafety

All protocols involving BoNT were approved by the SFBR Biohazards and Safety Committee and carried out under the CDC Select Agent Program following all applicable federal guidelines.

### Llama Immunization

Institutional Animal Care and Use Committee (IACUC) approval for this experiment was through the Triple J Farms (Bellingham, WA) protocol application process. A single male llama (*Lama glama*) named “Snoop” was immunized six times at 3 week intervals with a cocktail of toxoided botulinum neurotoxins. Snoop is kept with ten other male llamas ranging in age from 4 to 20 years of age. All llamas are housed in an eight acre grass paddock and have free access to a barn enclosure. All bleeds and injections are done in the barn enclosure with the herd mates present so as not to cause undue stress. The llamas used are acclimatized to being handled and bled, so no anesthesia is necessary. Triple J Farms IACUC committee inspects the facilities at six month intervals and USDA inspections are done by their veterinarians at least once a year. The first immunization was in Freunds complete adjuvant and subsequent immunizations were in Freunds incomplete adjuvant, all being one subcutaneous injection. Each dose was 1 mL total, 500 µL of which was adjuvant and 500 µL was phosphate buffered saline (PBS) containing 10 µg of each toxin serotype as formalin cross-linked toxoid. A 5 mL serum sample was taken one week prior to each immunization for analysis of seroconversion, and a full bleed of 800 mL was taken 3 weeks after the final immunization.

### Seroconversion Enzyme Linked Immunosorbant Assay (ELISA)

100 µL of 1 µg/mL of antigen in PBS was used to coat high binding ELISA plate wells overnight at 4°C. Plates were washed 3 times with 175 µL PBS and blocked with 300 µL of PBS +2% Carnation non fat dried milk (PBSM) for 1 h. Dilutions of serum in PBSM were then applied for 1 h, the plates washed 3x with 175 µL PBS +0.1% Tween-20 (PBST) and 2x with PBS. 100 µL of a 1 in 10,000 dilution of goat anti-llama horseradish peroxidase conjugate (Bethyl laboratories, Montgomery, TX) in PBSM was applied for 1 h and the plates washed again. TMB-Ultra (Pierce, Rockford, IL) was used for color development with sulfuric acid stop solution and absorbances read on a microplate reader (BioRad, Hercules, CA).

### Isolating Antibody Genes

White blood cells were first separated from half of the whole blood using UNI-SEPmaxi+ columns (Novamed, Jerusalem, Israel) and then total RNA was extracted using Trizol (Invitrogen, Carlsbad, CA). 10 µg of RNA was used in multiple 20 µL oligo-dT primed reverse transcription reactions (Ambion, Austin, TX) to generate cDNA. 2 µL aliquots were then used in 24×100 µL polymerase chain reaction (PCR) volumes with Hotstart YieldAce (Stratagene, La Jolla, CA) using 95°C for 5 min, 25×(95°C for 30 s, 50°C for 30 s, 72°C for 30 s) and 72°C for 5 min. The front primers were specific for the framework (FR) 1 region of llama variable heavy domains and encoded a *Pst* I site (“PstN1” = 5′-VAGGTSMARCTGCAGSAGTCWGG-3′ [80] and “PstN2” = 5′-GATGTGCAGCTGCAGGCGTCTGGRGGAGG-3′ [81]). The back primer was specific for framework 4 and encoded a *Not* I site (“GenNot” 5′-AAAAAAGCGGCCGCTGAGGAGACGGTGACCTG-3′ based upon [82]). PCR products were phenol chloroform extracted and ethanol precipitated, digested with *Pst* I and *Not* I and ligated to similarly digested phage display vector pecan21 LgEBOZg which has an anti-Ebola NP sdAb providing the FR1 and FR4 scaffold (unpublished observations). Home-made electrocompetent XL-1 Blue were used in over 120 electroporations to make the library of >1e+9 transformants. 24 clones were miniprepped, mapped and sequenced to gauge the fidelity of the library, which was then rescued with M13K07 and aliquots of phage stored at −80°C long term.

### Phage Selection and Screening for Anti-BoNT sdAb

100 µg amounts of toxins were individually biotinylated in 400 µL reactions using Sulfo-NHS-LC-Biotin (Pierce) and purified on Zeba Desalt Spin Columns (Pierce). Biotinylation was confirmed by comparing neutravidin capture efficiencies of modified and unmodified toxins in ELISA employing Snoop serum from the final bleed and anti-llama HRP as above (data not shown).

In a 500 µL volume, 100 representations of each clone or 1e+11 phagemids were combined with 10 µL streptavidin coated M-280 magnetic beads (Dynal Biotech ASA, Oslo, Norway) on a rotisserie to pre-absorb background binders for 1 h in PBS+2% bovine serum albumin+0.05% Tween-20 (PBSBT). 100 nM of target biotinylated toxin and 100 nM of each of the 6 unbiotinylated non-target or decoy toxins were assembled in 500 µL PBST and left to block for an hour. After magnetic capture of the beads, the supernatant containing blocked phage was combined with the toxin mix and rotissaried for 1 h. 10 µL of beads that had been blocked in 1 mL of PBSBT were magnetically captured, the supernatant removed, the phage/toxin mix added and rotated for 30 min to capture the biotinylated toxin and any specifically bound phage. The beads were then captured and washed 5x with 900 µL of PBSBT over the course of about 10 min. Phage remaining on the toxin were eluted with 500 µL 100 mM triethylamine for 10 min and neutralized with 250 µL of 0.5 M Tris-HCl pH 7.5. Half of this mix was used to infect exponential phase XL1-Blue cells which were plated on selective medium and rescued by super infection the next day according to standard practices [83]. 2 to 4 rounds of panning were performed at 100, 20, 4 and 0.8 nM antigen concentration, with many clones isolated after a single round. Polyclonal phage from round 2 was analyzed on all 7 serotypes of toxins and toxin complexes to determine if antigen specific clones were being enriched and a minimum of 96 clones from each panning round was analyzed by monoclonal ELISA on toxin as described above but employing anti-M13HRP conjugate (GE Healthcare, Piscataway, NJ) as the secondary antiserum. Positive clones having signals greater than 10x background were sequenced, amino acid sequences predicted using BioEdit [84] and unique clones identified using Multalin [85].

### Isolating sdAb Proteins

Unique clones were mobilized from the phage display vector to a soluble sdAb expression vector pecan45 LgEBOZg by *Pst* I/*Not* I and resequenced before transfer to Rosetta (Novagen/EMD Chemicals, Gibbstown, MD) for protein expression. Briefly, saturated 40 mL overnight cultures grown in terrific broth (TB) plus 2% glucose at 30°C were transferred to 400 mL of fresh TB without glucose and shaken for 3 h at 25°C. Expression was induced by addition of IPTG to 1 mM for 3 h at 25°C, the cells pelleted (typical wet weights of 8–9 g) and osmotically shocked [86] by resuspension in 14 mL ice-cold 0.75 M sucrose in 100 mM Tris-HCl pH 7.5, addition of 1.4 mL of 1 mg/mL hen egg lysozyme, followed by drop-wise addition of 28 mL of 1 mM EDTA pH 7.5 and swirling on ice for 15 min. 2.0 mL of 0.5 M MgCl_2_ was added, swirling continued for 15 min and cells pelleted. The 45 mL supernatant (shockate) was mixed with 5 mL of 10xIMAC (immobilized metal affinity chromatography buffer - 0.2 M Na_2_HPO_4_, 5 M NaCl, 0.2 M imidazole, 1% Tween-20, pH 7.5), followed by 0.5 mL of High Peformance Ni Separose (GE Healthcare) and the suspension gently mixed on ice for 1 h. Resin was pelleted and washed twice with 2×40 mL of 1xIMAC solution before elution with 2 mL of 0.45 M EDTA in 1xIMAC buffer. Proteins were concentrated in Amicon 10 kDa ultrafiltration devices (Millipore, Billerica, MA) to 200 µL for separation by gel filtration on a Superdex 200 HR 10/300 column (GE Healthcare) operating in PBS. Proteins were quantified by BCA assay (Pierce) and 10 µg analysed by SDS-PAGE and silver staining for impurities.

### Characterizing sdAb Proteins

To generate captor motifs, 10 µg of antibody was coupled to Bioplex beads (BioRad) according to the manufacturer's instructions to yield 150 µL of bead suspension. To generate tracer motifs, 200 µg of protein was biotinylated with a 10 fold molar excess of Sulfo-LC-NHS-biotin and purified as for the toxins to yield 0.5 µg/uL solutions.

Cross-reactivity assays were performed in duplicate by combining 0.125 µL of each of the beadsets against a particular serotype made in 50 µL of PBSBT with either 1e+5 pg of cognate toxin, cognate toxin complex or 1e+5 pg of each of the other toxin or toxin complex serotypes made in 50 µL. These mixes were incubated with shaking in the dark for 30 min and then washed by vacuum filtration twice with 175 µL PBSBT. 0.4 µL of a single biotinylated tracer sdAb in 100 µL PBSBT (to give approx. 133 nM) was added to the wells, incubated with shaking for 30 min and washed as above. 100 µL of PBSTB containing 2.5 µL/mL PhycoLink strepatavidin-PE PJ31S (PROzyme, San Leandro, CA) was added, wells shaken for 30 min, washed twice and the beads resuspended in 130 µL of PBSBT. Plates were read in a Bioplex (Biorad) with 100 events collected from each region to yield a series of median fluorescence intensities (MFI). The captor tracer pairs with mean MFI below 500 were discarded, and remaining pairs tabulated for percentage cross-reactivities.

LOD assays were essentially performed as above except threefold serial dilutions of toxin or toxin complex from 1e+4 pg/well in duplicate were employed in place of the fixed 1e+5 pg/well concentrations. Plots of duplicate MFI versus concentration were used to evaluate the lower LOD by using a value of 10 fold above background (set as non-cognate mMFI given by 1e+5 pg/well).

Heptaplex assays were performed by combining all of the selected pairs of beads and tracers, challenging them in duplicate with 1e+4 pg/well of each of the toxins or toxin complexes diluted in buffer, 2% reduced fat milk, orange juice (some pulp), carrot juice or cola and plotting duplicate MFIs. The milk and orange juice were microfuged prior to mixing with the beads.

### Neuro-2A Intoxication Assay

Neuro-2A assays were performed by combining 10 µg of sdAb with 2 µg of toxin in 0.5 mL of Eagle's minimum essential medium with Earle's balanced salt solution, non essential amino acids, sodium pyruvate (ATCC, Manassas, VA), 5% FBS and penicillin and streptomycin, incubating for 1 h at 37°C and then using the mix to replace supernatant on 90% confluent neuro-2A cells (ATCC, CCL-131) in 24 well plates. After incubation for 48 hours at 37°C in 5% CO_2_ in a humidified incubator the supernatant was aspirated, the cells were washed with serum free media and then lysed with 75 µL of 20 mM HEPES, 50 mM NaCl, 1% Triton X-100, pH 7.4 plus protease inhibitor cocktail (Roche, Nutley, NJ). 30 µL was combined with 75 µL of Laemmli sample buffer, boiled for 5 min and loaded on a 12% SDS-PAGE gel. Following semi-dry transfer to Immobilon P the membranes were blocked in 2% milk in PBS (MPBS) overnight. Probing was with 1 in 1000 of mouse anti-SNAP, clones SP12 and 4H251 (Santa Cruz Biotechnology, Santa Cruz, CA) and 1 in 5000 of mouse anti-actin clone C4 (Santa Cruz Biotechnology) followed by 1 in 10,000 of goat anti mouse IgG (H+L) HRP (Pierce, Rockford, IL). Pico west (Pierce) and Fuji X-ray film were used to develop and capture the images. The entire assay was repeated several times to try and generate the clearest images, and the same subset of clones appeared to show inhibitory effect each time.

### Circular Dichroism of sdAb and Conventional Immunoglobulins

Polyclonal antisera and sdAb were used at a concentration of 0.2 µg/µL in PBS. Data was collected in a 1 mm path length cuvette at 216 nm with a JASCO J-815 CD spectropolarimeter equipped with a temperature controlled Peltier cell holder. Data points were collected every 0.5°C and the temperature was increased from 20°C to 80 or 90°C and reversed at a rate of 10°C/min. To calculate the percentage refolded, the data points at 80°C were taken as 100% unfolded and values between 20 and 40°C on the curves were averaged and taken as the folded values. The folded value of the cooling curve (1 or 2) was subtracted from heating curve (1 or 2) and then divided by the 100% unfolded value.

## Supporting Information

Table S1 Chequerboard cross-reactivity assays of sdAb clones on toxins and complexes to identify best captor/tracer combinations. Duplicate median fluorescent intensities (MFI) and mean MFI (mMFI) calculated for tracer/captor pairs employing 1e+5 pg of target versus non-target serotype to evaluate the percentage cross-reactivity (% x-reactivity) S1) A toxin, S2) A complex, S3) B toxin, S4) B complex, S5) C toxin, S6) C complex, S7) D toxin, S8) D complex, S9) E toxin, S10) E complex, S11) F toxin, S12) F complex, S13) G toxin, S14) G complex. The most specific combinations with the largest expected dynamic range selected for further study in limit of detection trials are highlighted in yellow.
(0.61 MB PDF)

Figure S1 Primary structures of anti-BoNT sdAb clones. Predicted amino acid sequences of sdAb identified as positive by monoclonal phage ELISA on each serotype of toxin: a) A toxin; b) B toxin c) C toxin d) D toxin, e) E toxin, f) F toxin, g) G toxin.
(0.13 MB PDF)

Figure S2 Examining the refoldabilty of sdAb in contrast to conventional immunoglobulins. Circular dichroism analysis of our final captor tracer pairs of sdAb specific for each serotype versus polyclonal immunoglobulins for those serotypes that were available: a) A18, b) A17, c) B4, d) B2, e) C1, f) C24, g) D22, h) D16, i) E7, j) E4, k) F9, l) F5, m) G20, n) G3 o) A Ig, p) B Ig, q) C Ig, r) E Ig, s) F Ig. First heating = red, first cooling = dark blue, second heating = green, second cooling = light blue.
(0.15 MB PDF)
