# Supplementary material for: Llama Single Domain Antibodies Specific for the 7 Botulinum Neurotoxin Serotypes as Heptaplex Immunoreagents
Source: PLoS One. 2010 Jan 21;5(1):e8818. doi: 10.1371/journal.pone.0008818 (PMC2809108; doi:10.1371/journal.pone.0008818)
Supplement: Table S1 — Chequerboard cross-reactivity assays of sdAb clones on toxins and complexes to identify best captor/tracer combinations. Duplicate median fluorescent intensities (MFI) and mean MFI (mMFI) calculated for tracer/captor pairs employing 1e+5 pg of target versus non-target serotype to evaluate the percentage cross-reactivity (% x-reactivity) S1) A toxin, S2) A complex, S3) B toxin, S4) B complex, S5) C toxin, S6) C complex, S7) D toxin, S8) D complex, S9) E toxin, S10) E complex, S11) F toxin, S12) F complex, S13) G toxin, S14) G complex. The most specific combinations with the largest expected dynamic range selected for further study in limit of detection trials are highlighted in yellow. (0.61 MB PDF) [file pone.0008818.s001.pdf]

Table S1. Anti-A clones on toxins

Captor	Tracer	A MFI 1	A MFI 2	A mMFI	Non-A MFI 1	Non-A MFI 2	Non-A mMFI	% x-reactivity
1	3	4224	3623.5	3923.8	7	5	6.0	0.2
1	4	1547.5	1518	1532.8	4	4	4.0	0.3
1	6	566	575	570.5	2	2	2.0	0.4
1	7	1432	1238	1335.0	2.5	2.5	2.5	0.2
1	8	1755	1848	1801.5	2	2	2.0	0.1
1	9	172.5	208.5	190.5	3	3	3.0	1.6
1	11	1162	1057	1109.5	17.5	12.5	15.0	1.4
1	12	1118	1059.5	1088.8	3	4	3.5	0.3
1	13	2467	2137	2302.0	2	2	2.0	0.1
1	15	2640.5	2707	2673.8	2.5	2	2.3	0.1
1	17	1740	1916.5	1828.3	2	2	2.0	0.1
1	18	3985.5	4219	4102.3	4	2	3.0	0.1
2	3	4965	5076	5020.5	8	5	6.5	0.1
2	4	2294	2257	2275.5	6	4	5.0	0.2
2	6	1148.5	1133	1140.8	2	4	3.0	0.3
2	7	2163	2331	2247.0	3	2	2.5	0.1
2	8	2513	2662	2587.5	3	3	3.0	0.1
2	11	1917	1819.5	1868.3	27	28	27.5	1.5
2	12	1857	2061	1959.0	3	2	2.5	0.1
2	13	2978	2884	2931.0	3	2	2.5	0.1
2	15	2992	3267.5	3129.8	2	2	2.0	0.1
2	17	2544	2509	2526.5	2	3	2.5	0.1
2	18	4677	4613	4645.0	3	3	3.0	0.1
3	1	6234	6527	6380.5	4	5	4.5	0.1
3	2	6952.5	6702.5	6827.5	3	5	4.0	0.1
3	4	6232	5518	5875.0	10	5	7.5	0.1
3	5	5246	5482.5	5364.3	2	3	2.5	0.0
3	6	2642	2946	2794.0	1	2	1.5	0.1
3	7	4871	5134.5	5002.8	1	2	1.5	0.0
3	8	4840	4610.5	4725.3	2	0	1.0	0.0
3	9	5817	6559.5	6188.3	3	2	2.5	0.0
3	11	5737.5	4499	5118.3	32.5	37	34.8	0.7
3	12	5833	5783	5808.0	2	2	2.0	0.0
3	13	4940	4885	4912.5	1	1	1.0	0.0
3	16	5858	6251.5	6054.8	1	1	1.0	0.0
3	17	6601	7673	7137.0	2	2	2.0	0.0
3	18	601	572	586.5	1	1	1.0	0.2
4	1	3004	2927	2965.5	2	1.5	1.8	0.1
4	2	3061	3092	3076.5	1	1	1.0	0.0
4	3	2380	1960	2170.0	2	1	1.5	0.1
4	4	945	931	938.0	2	1	1.5	0.2

4	5	2478	2269	2373.5	1	1	1.0	0.0
4	6	556	574.5	565.3	1	1	1.0	0.2
4	8	1381.5	1220.5	1301.0	1	1	1.0	0.1
4	9	2917	2879	2898.0	1.5	2	1.8	0.1
4	11	2654	2441.5	2547.8	14	15	14.5	0.6
4	12	2428.5	2429	2428.8	3	2	2.5	0.1
4	13	1708.5	1646	1677.3	1	1	1.0	0.1
4	15	1079.5	1198.5	1139.0	1	0	0.5	0.0
4	16	3000	2966.5	2983.3	1	1	1.0	0.0
4	17	2367	2838	2602.5	2	1	1.5	0.1
4	18	2044	1928	1986.0	1	1	1.0	0.1
5	3	3774	3659	3716.5	7	4	5.5	0.1
5	4	1640	1450	1545.0	3	2.5	2.8	0.2
5	6	715	642	678.5	2	1	1.5	0.2
5	7	1546.5	1291	1418.8	1	1	1.0	0.1
5	8	1707	1868.5	1787.8	1	2	1.5	0.1
5	11	1413.5	1351	1382.3	71	58	64.5	4.7
5	12	1195	1240	1217.5	2	2	2.0	0.2
5	13	2016	2074	2045.0	1	1	1.0	0.0
5	15	2037	2321	2179.0	1	1	1.0	0.0
5	17	1567	1487.5	1527.3	1	1	1.0	0.1
5	18	3173	3329.5	3251.3	2	1	1.5	0.0
6	1	838	764.5	801.3	4	1	2.5	0.3
6	2	756	862	809.0	1	2	1.5	0.2
6	3	2048	1869.5	1958.8	4	2	3.0	0.2
6	5	671.5	633	652.3	2	2	2.0	0.3
6	8	608	714.5	661.3	1	1	1.0	0.2
6	9	715.5	746	730.8	2	2	2.0	0.3
6	13	981.5	841	911.3	1.5	1	1.3	0.1
6	15	1197	1199	1198.0	2	2	2.0	0.2
6	16	821	938	879.5	1	1	1.0	0.1
6	17	901	1077	989.0	2	1	1.5	0.2
6	18	1826	1726	1776.0	2.5	2	2.3	0.1
7	1	2417	3010	2713.5	1	2	1.5	0.1
7	2	2936	3006	2971.0	1	1	1.0	0.0
7	3	2937.5	2552.5	2745.0	2	1	1.5	0.1
7	4	756	794	775.0	1	1	1.0	0.1
7	5	2536.5	2986	2761.3	2	1	1.5	0.1
7	6	737.5	631	684.3	1	1	1.0	0.1
7	8	1540	1318	1429.0	1	2	1.5	0.1
7	9	2611	3198	2904.5	1	1	1.0	0.0
7	11	2594.5	2625	2609.8	10.5	9	9.8	0.4
7	12	2421.5	2217	2319.3	1	1	1.0	0.0
7	13	1865	1376	1620.5	1	1	1.0	0.1
7	15	1344	1461	1402.5	1	2	1.5	0.1
7	16	3484.5	3463	3473.8	1	1	1.0	0.0

7	17	2696.5	2573	2634.8	1	1	1.0	0.0
7	18	2455	2565	2510.0	1	1	1.0	0.0
8	1	2756	2462	2609.0	4	3	3.5	0.1
8	2	2683.5	3111	2897.3	2	2	2.0	0.1
8	3	3170	3154	3162.0	3.5	3	3.3	0.1
8	4	2219	1628	1923.5	2.5	2	2.3	0.1
8	5	2287.5	2145	2216.3	2	1.5	1.8	0.1
8	6	1013	1040	1026.5	1	2	1.5	0.1
8	7	1250	1463	1356.5	1	2	1.5	0.1
8	9	1804	2461	2132.5	2	1	1.5	0.1
8	11	3185.5	2600	2892.8	9	12.5	10.8	0.4
8	12	2003.5	2462	2232.8	2	2	2.0	0.1
8	15	2014.5	2779	2396.8	2	2	2.0	0.1
8	16	2498.5	3260.5	2879.5	1	1	1.0	0.0
8	17	2085	2991.5	2538.3	1	2	1.5	0.1
8	18	3044.5	2578	2811.3	1	1	1.0	0.0
9	3	4470	4399	4434.5	22	13	17.5	0.4
9	4	2094	1809	1951.5	16	12	14.0	0.7
9	6	961	760	860.5	11	8	9.5	1.1
9	7	2079	2147	2113.0	12	8.5	10.3	0.5
9	8	2329.5	2305	2317.3	9	10	9.5	0.4
9	11	1958	1580	1769.0	44	47	45.5	2.6
9	12	1723	1608	1665.5	15	11	13.0	0.8
9	13	2662.5	2449	2555.8	9	8	8.5	0.3
9	15	2250	2976	2613.0	7.5	8	7.8	0.3
9	17	2143.5	2094	2118.8	7	8	7.5	0.4
9	18	3689	3851	3770.0	7	7	7.0	0.2
11	1	1408.5	1805	1606.8	6	5	5.5	0.3
11	2	1761	1980	1870.5	4	4	4.0	0.2
11	3	4899	4648	4773.5	10	9	9.5	0.2
11	4	3127	2971	3049.0	6	7	6.5	0.2
11	5	1580	1654.5	1617.3	2.5	4	3.3	0.2
11	6	1059	1113	1086.0	3	3	3.0	0.3
11	7	2778	3093	2935.5	5.5	2	3.8	0.1
11	8	2532	2674	2603.0	4	3	3.5	0.1
11	9	1982	2207	2094.5	4	3.5	3.8	0.2
11	13	2992	2633	2812.5	3	3	3.0	0.1
11	15	2971	3698	3334.5	3	3	3.0	0.1
11	16	1538	1782	1660.0	3	2	2.5	0.2
11	17	4548	4284	4416.0	3	2	2.5	0.1
11	18	3964.5	4215	4089.8	4	3	3.5	0.1
12	1	2218	2521	2369.5	3	2	2.5	0.1
12	2	2711	2241	2476.0	3	3	3.0	0.1
12	3	4875	4263	4569.0	3	4	3.5	0.1
12	4	3026	3213	3119.5	4.5	3	3.8	0.1
12	5	2049	1844.5	1946.8	3	2	2.5	0.1

12	6	1235	1092	1163.5	2	3	2.5	0.2
12	7	2613	3032.5	2822.8	1.5	3	2.3	0.1
12	8	2407.5	2435	2421.3	2	3	2.5	0.1
12	9	2499.5	2685	2592.3	2.5	3	2.8	0.1
12	13	2753.5	2448	2600.8	3	2	2.5	0.1
12	15	3406	3691.5	3548.8	2	3	2.5	0.1
12	16	2180	2646	2413.0	3	1	2.0	0.1
12	17	4493	4963.5	4728.3	2	2	2.0	0.0
12	18	4184	4537	4360.5	2	3	2.5	0.1
13	15	1017.5	1336	1176.8	1	1	1.0	0.1
13	16	1606	1600.5	1603.3	0.5	1	0.8	0.0
13	17	1927	2241	2084.0	1	1	1.0	0.0
13	18	1859	1793	1826.0	1	1	1.0	0.1
16	3	5252	4226	4739.0	10.5	5	7.8	0.2
16	4	1603.5	1716	1659.8	3	3	3.0	0.2
16	6	661.5	676.5	669.0	1	2	1.5	0.2
16	7	1378	1678	1528.0	2	2	2.0	0.1
16	8	1739.5	1664	1701.8	3	2	2.5	0.1
16	11	1157	1342	1249.5	24	19	21.5	1.7
16	12	1219	1268.5	1243.8	2	3	2.5	0.2
16	13	2342	2063	2202.5	2	1	1.5	0.1
16	15	2735	2629	2682.0	1	1.5	1.3	0.0
16	17	1645.5	1758	1701.8	2	1.5	1.8	0.1
16	18	3707.5	4005	3856.3	2	3	2.5	0.1
17	1	1789	1668.5	1728.8	2	2	2.0	0.1
17	2	2036	1919	1977.5	1	1	1.0	0.1
17	3	1829.5	1610.5	1720.0	3	1	2.0	0.1
17	4	608.5	641	624.8	1.5	1.5	1.5	0.2
17	5	1474	1502	1488.0	1	1	1.0	0.1
17	6	510	434	472.0	1	1	1.0	0.2
17	8	840	772	806.0	2	1	1.5	0.2
17	9	1992	1827	1909.5	1	1.5	1.3	0.1
17	11	2006	1916	1961.0	11	10	10.5	0.5
17	12	1755.5	1815	1785.3	1	2	1.5	0.1
17	13	1079	980	1029.5	1	1	1.0	0.1
17	15	865.5	938	901.8	1.5	0	0.8	0.1
17	16	1763	1864	1813.5	1	1	1.0	0.1
17	17	1503.5	1608.5	1556.0	1	1	1.0	0.1
17	18	1732	1514	1623.0	1	2	1.5	0.1
18	1	6081	6573.5	6327.3	6	5	5.5	0.1
18	2	7068	6612	6840.0	3	3	3.0	0.0
18	4	5279	5435	5357.0	9	6	7.5	0.1
18	5	5895	5993	5944.0	2	5	3.5	0.1
18	6	2900	2820	2860.0	1	1	1.0	0.0
18	7	4037	4440	4238.5	2	2	2.0	0.0
18	8	4325.5	3955	4140.3	2	2	2.0	0.0

18	9	6649	6397.5	6523.3	3	2	2.5	0.0
18	11	5855	5552	5703.5	6	7	6.5	0.1
18	12	5891.5	5688	5789.8	2	1	1.5	0.0
18	13	4236.5	4302	4269.3	2	2	2.0	0.0
18	16	6197.5	6161	6179.3	2	1	1.5	0.0
18	17	7272	7426	7349.0	2	2.5	2.3	0.0
18	18	568	574.5	571.3	1	1	1.0	0.2

Table S2. Anti-A clones on complexes

Captor	Tracer	Ac MFI 1	Ac MFI 2	Ac mMFI	Non-Ac MFI 1	Non-Ac MFI 2	Non-Ac mMFI	% x-reactivity
1	3	1017	1057	1037.0	4	11	7.5	0.7
1	13	659	612.5	635.8	2	2	2.0	0.3
1	15	584	607.5	595.8	2	2	2.0	0.3
1	18	883	1093	988.0	2	5	3.5	0.4
2	3	1587	1661	1624.0	5.5	30	17.8	1.1
2	4	679.5	627	653.3	3	2	2.5	0.4
2	7	563	581	572.0	3	2	2.5	0.4
2	8	761.5	659.5	710.5	2	3	2.5	0.4
2	11	697	611	654.0	25	22.5	23.8	3.6
2	12	496	515	505.5	3	3	3.0	0.6
2	13	976	1076	1026.0	2	2	2.0	0.2
2	15	935.5	1145	1040.3	2	2	2.0	0.2
2	17	650	659	654.5	2	2	2.0	0.3
2	18	1358	1640	1499.0	3	6	4.5	0.3
3	1	2291	2285	2288.0	7	13	10.0	0.4
3	2	2487	2315	2401.0	3	2	2.5	0.1
3	4	2967.5	2540	2753.8	3	2	2.5	0.1
3	5	1770	1950	1860.0	2	2	2.0	0.1
3	6	1145.5	1253	1199.3	1	2	1.5	0.1
3	7	2565	2237	2401.0	3	2	2.5	0.1
3	8	1888	2124.5	2006.3	0	1.5	0.8	0.0
3	9	2238	2542.5	2390.3	4	4	4.0	0.2
3	11	3131.5	2791	2961.3	44	27	35.5	1.2
3	12	2733	2975	2854.0	1	1	1.0	0.0
3	13	2363	2551	2457.0	1	1	1.0	0.0
3	16	2156.5	2100	2128.3	1	1	1.0	0.0
3	17	3315.5	3800	3557.8	2	1	1.5	0.0
4	11	539.5	442	490.8	18	18	18.0	3.7
5	3	1290	808	1049.0	3	14	8.5	0.8
5	8	456	543	499.5	2	1	1.5	0.3
5	11	551.5	521	536.3	72	73	72.5	13.5
5	13	778.5	767	772.8	1	1	1.0	0.1
5	15	714	743.5	728.8	1	1	1.0	0.1
5	17	525	542	533.5	2	2	2.0	0.4
5	18	1143	1333	1238.0	3	3	3.0	0.2
6	3	875	733	804.0	2	8	5.0	0.6
6	18	699.5	835	767.3	2	2	2.0	0.3
8	3	673	712.5	692.8	1.5	5	3.3	0.5
8	18	564.5	681	622.8	3	1	2.0	0.3
9	3	1297	1307	1302.0	12	29	20.5	1.6
9	4	560	522	541.0	12	14	13.0	2.4
9	7	521.5	448.5	485.0	11	8	9.5	2.0
9	8	550.5	564	557.3	11	9	10.0	1.8
9	11	559	457	508.0	50.5	37	43.8	8.6
9	13	785	675	730.0	5	9	7.0	1.0

9	15	808	1040	924.0	7	5	6.0	0.6
9	17	464.5	619	541.8	10	6	8.0	1.5
9	18	964.5	1367	1165.8	12	12	12.0	1.0
11	3	2484.5	2522.5	2503.5	3.5	28	15.8	0.6
11	4	1333	1448	1390.5	5	3.5	4.3	0.3
11	7	1584	1265	1424.5	3.5	5	4.3	0.3
11	8	1088	1170	1129.0	4	2.5	3.3	0.3
11	13	1332	1511.5	1421.8	3	3	3.0	0.2
11	15	1668	1881	1774.5	3	3	3.0	0.2
11	17	1608.5	2069.5	1839.0	3	2	2.5	0.1
11	18	2094	2477	2285.5	4	6	5.0	0.2
12	3	1199	1393	1296.0	3	6	4.5	0.3
12	4	944	722	833.0	4	2	3.0	0.4
12	7	616	677	646.5	3	2	2.5	0.4
12	13	640	682.5	661.3	2		2.0	0.3
12	15	800	1044	922.0	3	2	2.5	0.3
12	17	1187	1295.5	1241.3	3	2	2.5	0.2
12	18	1173	1292.5	1232.8	2	3	2.5	0.2
16	3	1469	1492.5	1480.8	3	19	11.0	0.7
16	4	514	413	463.5	2	2	2.0	0.4
16	8	547	549	548.0	1	2.5	1.8	0.3
16	11	541	452	496.5	22	22	22.0	4.4
16	13	824	789	806.5	1	1	1.0	0.1
16	15	810	1053	931.5	2	1	1.5	0.2
16	17	405	521	463.0	2	1	1.5	0.3
16	18	1433	1340	1386.5	3	4	3.5	0.3
18	1	2671	2810.5	2740.8	13	21	17.0	0.6
18	2	3041	2959	3000.0	3	3	3.0	0.1
18	4	3212	2971.5	3091.8	2	2	2.0	0.1
18	5	2610	2439	2524.5	3	2	2.5	0.1
18	6	1487	1607	1547.0	1	2	1.5	0.1
18	7	2830	2624	2727.0	3	3	3.0	0.1
18	8	2379	2765	2572.0	2	1	1.5	0.1
18	9	2718	2905	2811.5	5	5	5.0	0.2
18	11	4064	3824.5	3944.3	6	8	7.0	0.2
18	12	3925	4096	4010.5	2	1	1.5	0.0
18	13	2825	2993	2909.0	1	1	1.0	0.0
18	16	2800.5	2732.5	2766.5	1	1	1.0	0.0
18	17	3989.5	4193	4091.3	2	2	2.0	0.0

Table S3. Anti-B clones on toxins

Captor	Tracer	B MFI 1	B MFI 2	B mMFI	Non-B MFI 1	Non-B MFI 2	Non-B mMFI	% x-reactivity
1	2	1046.5	2211	1628.8	3	3.5	3.3	0.2
1	3	1869	2924.5	2396.8	7	6	6.5	0.3
1	4	1205.5	2201.5	1703.5	5	4	4.5	0.3
1	5	2037.5	872	1454.8	7	3	5.0	0.3
1	6	978.5	622	800.3	4	9	6.5	0.8
1	7	1048	1011.5	1029.8	2.5	3	2.8	0.3
1	8	658	863	760.5	3.5	2	2.8	0.4
1	9	534	975	754.5	1.5	4	2.8	0.4
1	10	1571	1652	1611.5	16	10	13.0	0.8
1	13	1258.5	1773	1515.8	2	1	1.5	0.1
1	14	1044	913.5	978.8	5	7	6.0	0.6
1	17	2114	1577	1845.5	2	11	6.5	0.4
1	18	1977.5	2071	2024.3	3.5	3	3.3	0.2
1	19	751	639	695.0	7	4	5.5	0.8
1	20	445	584	514.5	2.5	9	5.8	1.1
1	21	690	665.5	677.8	4	4	4.0	0.6
2	2	1307.5	1738	1522.8	2	2.5	2.3	0.1
2	3	1426.5	1625	1525.8	2	3	2.5	0.2
2	4	1513	1364	1438.5	5	8	6.5	0.5
2	5	1255	1117	1186.0	2.5	0	1.3	0.1
2	6	1026	1013	1019.5	2	2	2.0	0.2
2	7	1253	639	946.0	1	4	2.5	0.3
2	8	627	566	596.5	7	3	5.0	0.8
2	9	621	666.5	643.8	0	5	2.5	0.4
2	10	1859.5	1743	1801.3	7.5	9	8.3	0.5
2	13	800	670	735.0	3	2	2.5	0.3
2	14	718	811	764.5	10	10	10.0	1.3
2	17	860	919	889.5	4	3	3.5	0.4
2	18	1307	1140	1223.5	3	1	2.0	0.2
2	19	547	431	489.0	9	7	8.0	1.6
2	20	993	725.5	859.3	1	3	2.0	0.2
3	1	579.5	383	481.3	2	5	3.5	0.7
3	2	2423	2056	2239.5	3	2	2.5	0.1
3	3	1869.5	1948	1908.8	3	6.5	4.8	0.2
3	4	1860	2051.5	1955.8	13	11	12.0	0.6
3	5	2007	2094	2050.5	10.5	9	9.8	0.5
3	6	1245.5	1396	1320.8	3	6	4.5	0.3
3	7	1743	1053.5	1398.3	4	5	4.5	0.3
3	8	888.5	1133	1010.8	3	4	3.5	0.3
3	9	1067	741.5	904.3	3.5	4	3.8	0.4
3	10	2295	2282.5	2288.8	15	13	14.0	0.6
3	13	1113.5	1068	1090.8	2	5	3.5	0.3
3	14	1209	914	1061.5	11	9	10.0	0.9
3	17	1315	1434	1374.5	6.5	5.5	6.0	0.4
3	18	1904	1719	1811.5	3	5	4.0	0.2

3	19	866	700	783.0	6.5	7	6.8	0.9
3	20	1321	1206	1263.5	3	4	3.5	0.3
3	21	668	631	649.5	6	8	7.0	1.1
4	1	528	652	590.0	4	1	2.5	0.4
4	2	1625	2118	1871.5	0.5	2	1.3	0.1
4	3	1632.5	2350.5	1991.5	2.5	1.5	2.0	0.1
4	4	2299	2127	2213.0	7	13	10.0	0.5
4	5	1576	1953	1764.5	4	5	4.5	0.3
4	6	1618.5	1344.5	1481.5	3.5	1	2.3	0.2
4	7	1593.5	1997	1795.3	1.5	1	1.3	0.1
4	8	669	946	807.5	3	5	4.0	0.5
4	9	1064	1150.5	1107.3	3	4	3.5	0.3
4	10	2517	3086.5	2801.8	14	13	13.5	0.5
4	13	1365	1306.5	1335.8	2.5	3	2.8	0.2
4	14	1200	1108.5	1154.3	8	15	11.5	1.0
4	17	1356.5	915	1135.8	3	7	5.0	0.4
4	18	1152	1607	1379.5	2	3	2.5	0.2
4	19	797	1020	908.5	6	2	4.0	0.4
4	20	1021	1289.5	1155.3	2	2.5	2.3	0.2
4	21	681.5	591	636.3	6	6.5	6.3	1.0
5	2	1151.5	2186	1668.8	1	4	2.5	0.1
5	3	1586	1512.5	1549.3	3	1	2.0	0.1
5	4	1840	1655	1747.5	3	4	3.5	0.2
5	5	1733	1444	1588.5	5	2	3.5	0.2
5	6	1198	1058	1128.0	1	1	1.0	0.1
5	7	1318	1802	1560.0	2	2	2.0	0.1
5	8	386	729	557.5	5.5	3.5	4.5	0.8
5	9	663	1022	842.5	2	3	2.5	0.3
5	10	1278	2233	1755.5	12.5	11	11.8	0.7
5	13	1203.5	1304.5	1254.0	3	2	2.5	0.2
5	14	1186	1004	1095.0	5	6	5.5	0.5
5	17	1227	1108	1167.5	3	4	3.5	0.3
5	18	1941.5	1739	1840.3	4	2.5	3.3	0.2
5	19	664	660	662.0	4.5	3	3.8	0.6
5	20	1249	1126	1187.5	1	3.5	2.3	0.2
6	2	471	647.5	559.3	3.5	1.5	2.5	0.4
6	3	446.5	749	597.8	3.5	1	2.3	0.4
6	5	573	554	563.5	4	6	5.0	0.9
6	18	463	529.5	496.3	1.5	4	2.8	0.6
7	2	1032	1127	1079.5	4	1	2.5	0.2
7	3	703	1281.5	992.3	2.5	2.5	2.5	0.3
7	4	1190	892	1041.0	7	14	10.5	1.0
7	5	1145	633	8.0	4		4.0	50.0
7	7	546	506	526.0	2.5	3	2.8	0.5
7	10	1002	910	956.0	7.5	13.5	10.5	1.1
7	13	545	860	702.5	3	0	1.5	0.2
7	14	650	627	638.5	15	27	21.0	3.3
7	17	748	817	782.5	3.5	3	3.3	0.4
7	18	1053	997.5	1025.3	2	3	2.5	0.2
10	3	545	438	491.5	1	2	1.5	0.3

10	4	561	588.5	574.8	5	0	2.5	0.4
13	2	747	930	838.5	2	0.5	1.3	0.1
13	3	582	809.5	695.8	10	3	6.5	0.9
13	4	826	799	812.5	9	3.5	6.3	0.8
13	5	417	614	515.5	3	3	3.0	0.6
13	6	642.5	470.5	556.5	2	3	2.5	0.4
13	7	596	699	647.5	2	1	1.5	0.2
13	10	1230	1144.5	1187.3	7	9	8.0	0.7
13	17	604	381	492.5	0.5	2	1.3	0.3
13	18	627	666.5	646.8	2.5	1	1.8	0.3
13	20	537	618	577.5	1	1	1.0	0.2
14	2	1282.5	1081	1181.8	0	2	1.0	0.1
14	3	851	840	845.5	6	3	4.5	0.5
14	4	627	754	690.5	20	6	13.0	1.9
14	5	922	813	867.5	4	2.5	3.3	0.4
14	6	850.5	486	668.3	1.5	5	3.3	0.5
14	7	642	797	719.5	6	0	3.0	0.4
14	9	530	368	449.0	3	2	2.5	0.6
14	10	1251	1309	1280.0	11	15	13.0	1.0
14	18	704	778	741.0	1.5	1.5	1.5	0.2
14	20	498	648.5	573.3	3.5	2.5	3.0	0.5
17	1	486	672	579.0	4	2.5	3.3	0.6
17	2	1949	1602	1775.5	4	2	3.0	0.2
17	3	1307	1303.5	1305.3	2	0	1.0	0.1
17	4	1204	1252	1228.0	3.5	10.5	7.0	0.6
17	5	1107.5	823	965.3	5.5	2	3.8	0.4
17	6	1259	1081	1170.0	3	2	2.5	0.2
17	7	1485	1396	1440.5	5	6	5.5	0.4
17	8	707.5	860.5	784.0	4.5	7	5.8	0.7
17	9	779.5	896	837.8	5	3	4.0	0.5
17	10	1976	2262	2119.0	12	19	15.5	0.7
17	13	660	699.5	679.8	2.5	1.5	2.0	0.3
17	14	604	451	527.5	7	11	9.0	1.7
17	17	894.5	955.5	925.0	6.5	3	4.8	0.5
17	18	1049	990.5	1019.8	0	4	2.0	0.2
17	19	517	464	490.5	3.5	7	5.3	1.1
17	20	1232	1160	1196.0	3	3	3.0	0.3
18	1	921	1207	1064.0	7	6	6.5	0.6
18	2	2256.5	2474.5	2365.5	5.5	5.5	5.5	0.2
18	3	1489	1495.5	1492.3	1	2	1.5	0.1
18	4	1681	1381	1531.0	11	12	11.5	0.8
18	5	1306	1344.5	1325.3	4.5	5	4.8	0.4
18	6	1917	1935.5	1926.3	6	7	6.5	0.3
18	7	1651	1965	1808.0	15	7	11.0	0.6
18	8	1110	1096	1103.0	9.5	6	7.8	0.7
18	9	1213.5	1203.5	1208.5	7.5	5	6.3	0.5
18	10	2906	2838.5	2872.3	15	25	20.0	0.7
18	13	1121	975.5	1048.3	4	4	4.0	0.4
18	14	751	501	626.0	22	11	16.5	2.6
18	17	1076	980	1028.0	11.5	8	9.8	0.9

18	18	1208	1582	1395.0	7.5	4.5	6.0	0.4
18	19	884	839	861.5	11	16	13.5	1.6
18	20	1801.5	1843	1822.3	4	7	5.5	0.3
18	21	661	717.5	689.3	10	8	9.0	1.3
20	2	1273	812	1042.5	0	6	3.0	0.3
20	3	0	1163.5	581.8	0	5	2.5	0.4
20	4	1044	593	818.5	5	3	4.0	0.5
20	5	0	652	326.0	2	8	5.0	1.5
20	7	2104.5	187.5	1146.0	2	5	3.5	0.3
20	10	526	581	553.5	0	14.5	7.3	1.3
20	13	673	506	589.5	2	0	1.0	0.2
20	14	368	761	564.5	7	9	8.0	1.4
20	17	727.5	654	690.8	7	16.5	11.8	1.7
20	18	1265	6601	3933.0	3	1	2.0	0.1
20	19	55	28612	14333.5	9	5	7.0	0.0
20	20	158	904	531.0	5	6	5.5	1.0

Table S4. Anti-B clones on complexes

Captor	Tracer	Bc MFI 1	Bc MFI 2	Bc mMFI	Non-Bc MFI 1	Non-Bc MFI 2	Non-Bc mMFI	% x-reactivity
1	2	8822.5	8190	8506.3	58.5	61	59.8	0.7
1	3	6698	7949	7323.5	14	9	11.5	0.2
1	4	7898	8052	7975.0	15	13.5	14.3	0.2
1	5	7899	8676	8287.5	9	17	13.0	0.2
1	6	2407.5	2836	2621.8	1283	400	841.5	32.1
1	7	2784	2880	2832.0	883	1287	1085.0	38.3
1	8	832	1416.5	1124.3	528.5	456.5	492.5	43.8
1	9	4729	3970	4349.5	43	49	46.0	1.1
1	10	3553	3974	3763.5	2162.5	1638	1900.3	50.5
1	11	503.5	520.5	512.0	408.5	309.5	359.0	70.1
1	13	4276.5	5302	4789.3	7	18	12.5	0.3
1	14	5845	6019	5932.0	15.5	12	13.8	0.2
1	17	7803	7779.5	7791.3	7	8.5	7.8	0.1
1	18	8946	9250	9098.0	9.5	13.5	11.5	0.1
1	19	2366	1982	2174.0	325	566.5	445.8	20.5
1	20	2550	2464	2507.0	832	794.5	813.3	32.4
1	21	1832	1967	1899.5	896.5	463	679.8	35.8
2	1	804	845	824.5	6	7	6.5	0.8
2	2	7656.5	7588.5	7622.5	3	4	3.5	0.0
2	3	7123	7643	7383.0	1	2	1.5	0.0
2	4	7979.5	7817.5	7898.5	8	6	7.0	0.1
2	5	6461	6954	6707.5	2	1	1.5	0.0
2	6	5055.5	4983.5	5019.5	82	99	90.5	1.8
2	7	5436	5797	5616.5	43	61	52.0	0.9
2	8	2798.5	2950	2874.3	50.5	42	46.3	1.6
2	9	4457	4973	4715.0	4	4	4.0	0.1
2	10	6422.5	9432	7927.3	160	154.5	157.3	2.0
2	11	718	736	727.0	25	25	25.0	3.4
2	13	6732	6719	6725.5	3	3	3.0	0.0
2	14	5431	5091	5261.0	12.5	11	11.8	0.2
2	15	523	448.5	485.8	36	33	34.5	7.1
2	17	7024	6504	6764.0	5	4	4.5	0.1
2	18	7405	6952	7178.5	3	2	2.5	0.0
2	19	3033	2902.5	2967.8	29	24	26.5	0.9
2	20	5078	5533	5305.5	40	46	43.0	0.8
2	21	2720	2586	2653.0	43	40	41.5	1.6
3	1	1046.5	1233	1139.8	5	6	5.5	0.5
3	2	10538	10092.5	10315.3	3	5	4.0	0.0
3	3	9987	8761	9374.0	4	2	3.0	0.0
3	4	9699	10089	9894.0	13	11.5	12.3	0.1
3	5	9410	9701.5	9555.8	5	5	5.0	0.1
3	6	6993	5942.5	6467.8	5.5	3.5	4.5	0.1
3	7	7366	6925.5	7145.8	5	4	4.5	0.1
3	8	3149.5	3825	3487.3	6	7	6.5	0.2
3	9	6109	5464	5786.5	8.5	7	7.8	0.1

3	10	8515.5	9111	8813.3	18	11	14.5	0.2
3	11	975	1302	1138.5	4	5	4.5	0.4
3	13	7316	8982	8149.0	2	2	2.0	0.0
3	14	7579	7127.5	7353.3	15	11.5	13.3	0.2
3	15	852	618.5	735.3	32	40	36.0	4.9
3	17	8784	8353	8568.5	4	6	5.0	0.1
3	18	9668	9880	9774.0	3.5	3	3.3	0.0
3	19	4094	3862.5	3978.3	9	14	11.5	0.3
3	20	6806	6951	6878.5	10	5	7.5	0.1
3	21	3619	3398.5	3508.8	8	7	7.5	0.2
4	1	1202	1165.5	1183.8	4	3	3.5	0.3
4	2	11260	11519	11389.5	2	2	2.0	0.0
4	3	10871	9827	10349.0	2	1	1.5	0.0
4	4	10979	11136.5	11057.8	6	6	6.0	0.1
4	5	9937	10382	10159.5	2	3	2.5	0.0
4	6	6595	6445	6520.0	2	5	3.5	0.1
4	7	7908	8456	8182.0	3.5	2	2.8	0.0
4	8	3881	4311	4096.0	3	5	4.0	0.1
4	9	5472.5	8042	6757.3	2.5	2	2.3	0.0
4	10	9074	10995.5	10034.8	13	12	12.5	0.1
4	11	1412	1125	1268.5	1.5	3	2.3	0.2
4	13	10644	8273.5	9458.8	4	2	3.0	0.0
4	14	9110	8096	8603.0	8	8.5	8.3	0.1
4	15	895	654	774.5	33	21	27.0	3.5
4	17	10489	9893	10191.0	4	4	4.0	0.0
4	18	10560	12072	11316.0	2.5	3.5	3.0	0.0
4	19	4572	4080	4326.0	8.5	7	7.8	0.2
4	20	7878.5	7721	7799.8	3	5	4.0	0.1
4	21	3754	4536.5	4145.3	5	8	6.5	0.2
5	1	954.5	1205	1079.8	4	3	3.5	0.3
5	2	11789	12022	11905.5	2	2	2.0	0.0
5	3	10550	10152.5	10351.3	3	2	2.5	0.0
5	4	10850.5	10667.5	10759.0	9	7.5	8.3	0.1
5	5	10418	10275.5	10346.8	3	1	2.0	0.0
5	6	6544	6449.5	6496.8	2	5	3.5	0.1
5	7	7445	7749.5	7597.3	1	2	1.5	0.0
5	8	2818.5	4315	3566.8	7	9	8.0	0.2
5	9	5237.5	6514	5875.8	7.5	4	5.8	0.1
5	10	9804	12619.5	11211.8	13.5	20	16.8	0.1
5	11	1307	839.5	1073.3	2	3	2.5	0.2
5	13	8917	8704.5	8810.8	1.5	2.5	2.0	0.0
5	14	8863	8177	8520.0	12.5	11	11.8	0.1
5	15	895	659	777.0	32	32	32.0	4.1
5	17	10407	10291	10349.0	5	5	5.0	0.0
5	18	10616	11672	11144.0	4	3	3.5	0.0
5	19	4914	4065.5	4489.8	10	9.5	9.8	0.2
5	20	6071	7589.5	6830.3	7	4.5	5.8	0.1
5	21	4064	3549	3806.5	6.5	6	6.3	0.2
6	2	5135.5	4593	4864.3	18.5	24	21.3	0.4
6	3	5042	5179	5110.5	3	2	2.5	0.0

6	4	5276	5619	5447.5	7	6	6.5	0.1
6	5	5164	5017	5090.5	2	3	2.5	0.0
6	6	1126.5	1100.5	1113.5	485	518	501.5	45.0
6	7	1848	1785.5	1816.8	981	709	845.0	46.5
6	8	216.5	586	401.3	212	298	255.0	63.6
6	9	1303.5	2302	1802.8	11	16	13.5	0.7
6	10	2048.5	2838	2443.3	1775	2326	2050.5	83.9
6	13	3801	4264.5	4032.8	8	7	7.5	0.2
6	14	4079	3764	3921.5	14	8.5	11.3	0.3
6	17	4532	4391	4461.5	4	4	4.0	0.1
6	18	5550	5815	5682.5	2	3.5	2.8	0.0
6	19	1003	901	952.0	240.5	206.5	223.5	23.5
6	20	1347.5	1421	1384.3	545.5	493	519.3	37.5
6	21	868.5	696	782.3	548	226	387.0	49.5
7	2	6182.5	5422	5802.3	108	138	123.0	2.1
7	3	5973	5720	5846.5	3	1	2.0	0.0
7	4	5136	5447.5	5291.8	6	6	6.0	0.1
7	5	5507	5360	5433.5	2	3	2.5	0.0
7	6	2484.5	2507.5	2496.0	1282.5	1214	1248.3	50.0
7	7	1982	2421	2201.5	1230.5	1097	1163.8	52.9
7	8	1193	1274	1233.5	260	720	490.0	39.7
7	9	3197	2679	2938.0	91	77	84.0	2.9
7	10	5142.5	6565	5853.8	1872	2731	2301.5	39.3
7	13	4712.5	4681.5	4697.0	9	15	12.0	0.3
7	14	4760.5	4528	4644.3	10	7	8.5	0.2
7	17	5045	4922	4983.5	4	5	4.5	0.1
7	18	5499	5683.5	5591.3	3	4	3.5	0.1
7	19	1211	1184	1197.5	434	687	560.5	46.8
7	20	2061	2223.5	2142.3	1366	1379	1372.5	64.1
7	21	1225	1520	1372.5	860	1002	931.0	67.8
9	2	830	888	859.0	3	4	3.5	0.4
9	3	869	821.5	845.3	2	3	2.5	0.3
9	4	873	867	870.0	4.5	4	4.3	0.5
9	5	837	813	825.0	3	2	2.5	0.3
9	9	574	391.5	482.8	3	4	3.5	0.7
9	10	675	683	679.0	34.5	21	27.8	4.1
9	13	697	856	776.5	1	3	2.0	0.3
9	14	605.5	528.5	567.0	8	9	8.5	1.5
9	17	642	706.5	674.3	4	3	3.5	0.5
9	18	773	824	798.5	1	2	1.5	0.2
10	2	5105	5195	5150.0	56.5	60	58.3	1.1
10	3	4446.5	4129	4287.8	2.5	2	2.3	0.1
10	4	4360	4765	4562.5	7	10	8.5	0.2
10	5	4626	4769.5	4697.8	2	2	2.0	0.0
10	6	1998	2105	2051.5	680	812.5	746.3	36.4
10	7	2644	2214	2429.0	745	790	767.5	31.6
10	8	1375	1164	1269.5	537	428	482.5	38.0
10	9	3018	2443	2730.5	30	38	34.0	1.2
10	10	3630	4690	4160.0	1478.5	1308.5	1393.5	33.5
10	13	4009	3613	3811.0	8	9	8.5	0.2

10	14	3369	3158	3263.5	9.5	7	8.3	0.3
10	17	3772	4031.5	3901.8	3	3.5	3.3	0.1
10	18	4975	4877.5	4926.3	5	4	4.5	0.1
10	19	1443.5	1251	1347.3	267	210	238.5	17.7
10	20	2449.5	2057.5	2253.5	624	473	548.5	24.3
10	21	995	935	965.0	284	345	314.5	32.6
11	2	1268	1346	1307.0	18	14	16.0	1.2
11	3	1360	1312	1336.0	2	1	1.5	0.1
11	4	1285.5	1232	1258.8	5.5	5	5.3	0.4
11	5	1257	1162	1209.5	2	3	2.5	0.2
11	9	576	571	573.5	13	15	14.0	2.4
11	10	521	649	585.0	533	421	477.0	81.5
11	13	703.5	805	754.3	3	3	3.0	0.4
11	14	813	742	777.5	7	9	8.0	1.0
11	17	965	882	923.5	4	2	3.0	0.3
11	18	1242	1184.5	1213.3	3	2	2.5	0.2
13	1	630	759	694.5	3.5	5	4.3	0.6
13	2	6464	6060.5	6262.3	2	3	2.5	0.0
13	3	6031	5795	5913.0	1	1	1.0	0.0
13	4	6097.5	6357	6227.3	7	8	7.5	0.1
13	5	5412	5513	5462.5	2	2	2.0	0.0
13	6	4604	4140	4372.0	10	12.5	11.3	0.3
13	7	4998	4592	4795.0	5	6	5.5	0.1
13	8	2576	2735	2655.5	10	11	10.5	0.4
13	9	2670	4106	3388.0	4	3	3.5	0.1
13	10	6238	7381.5	6809.8	27	23	25.0	0.4
13	11	549	675	612.0	5.5	5	5.3	0.9
13	13	4134	4627	4380.5	0	1	0.5	0.0
13	14	3872	4085	3978.5	8	7	7.5	0.2
13	17	5358	5254.5	5306.3	2.5	4	3.3	0.1
13	18	5888.5	5222	5555.3	2	3	2.5	0.0
13	19	2303.5	2245	2274.3	9.5	8.5	9.0	0.4
13	20	5517.5	4367.5	4942.5	10	5.5	7.8	0.2
13	21	2157.5	1896	2026.8	9	9.5	9.3	0.5
14	1	778	781	779.5	5	6	5.5	0.7
14	2	6525	7136	6830.5	4	2	3.0	0.0
14	3	6655.5	6476.5	6566.0	1	1	1.0	0.0
14	4	6051	6323	6187.0	11	9	10.0	0.2
14	5	6644	5998	6321.0	2	2	2.0	0.0
14	6	4749	4891	4820.0	3	6	4.5	0.1
14	7	5519	5201	5360.0	0	2	1.0	0.0
14	8	2796	2701	2748.5	6	8	7.0	0.3
14	9	3022	3591	3306.5	5	6.5	5.8	0.2
14	10	7116	7717	7416.5	14	12	13.0	0.2
14	11	668	690	679.0	3	3	3.0	0.4
14	13	5381.5	3668	4524.8	3	3	3.0	0.1
14	14	4815.5	4449	4632.3	15	11.5	13.3	0.3
14	17	5373	5467.5	5420.3	4	5	4.5	0.1
14	18	6283	6480	6381.5	3	3	3.0	0.0
14	19	2534.5	2587	2560.8	8	10	9.0	0.4

14	20	5776	4573	5174.5	4	6	5.0	0.1
14	21	2171	1940.5	2055.8	8	4	6.0	0.3
15	2	1564	1525.5	1544.8	10	11.5	10.8	0.7
15	3	1647	1405	1526.0	2	1	1.5	0.1
15	4	1333	1602	1467.5	6	6	6.0	0.4
15	5	1543	1456.5	1499.8	2	2	2.0	0.1
15	6	475	544	509.5	182	176	179.0	35.1
15	9	671.5	470	570.8	8.5	7	7.8	1.4
15	10	823	822	822.5	342.5	347	344.8	41.9
15	13	1229	1277.5	1253.3	4	4	4.0	0.3
15	14	1011	806.5	908.8	8	8	8.0	0.9
15	17	1254.5	1265	1259.8	3	3	3.0	0.2
15	18	1514.5	1495	1504.8	2.5	4	3.3	0.2
15	20	665	467	566.0	182.5	171	176.8	31.2
17	1	1287	1468	1377.5	4	4	4.0	0.3
17	2	9634	9924	9779.0	2	2.5	2.3	0.0
17	3	8639	9184	8911.5	2.5	2	2.3	0.0
17	4	7892	9097	8494.5	8	7	7.5	0.1
17	5	9107	7729	8418.0	2	2	2.0	0.0
17	6	6657	6778.5	6717.8	4	1.5	2.8	0.0
17	7	7470	8472.5	7971.3	2	3	2.5	0.0
17	8	3722.5	3838.5	3780.5	9	7	8.0	0.2
17	9	5551.5	6174	5862.8	7	3	5.0	0.1
17	10	7171	10234	8702.5	7	10	8.5	0.1
17	11	674	912	793.0	3.5	4	3.8	0.5
17	13	7006	6644	6825.0	2	1	1.5	0.0
17	14	7066.5	6658	6862.3	9	12	10.5	0.2
17	15	678.5	577	627.8	29	33.5	31.3	5.0
17	17	8013	7435.5	7724.3	3	5	4.0	0.1
17	18	9130	9074.5	9102.3	3	3	3.0	0.0
17	19	3638	3472	3555.0	10	9	9.5	0.3
17	20	7633	7298	7465.5	5	6	5.5	0.1
17	21	3077	3483	3280.0	5	6	5.5	0.2
18	1	1373	1595	1484.0	8	7	7.5	0.5
18	2	9770.5	9737	9753.8	5	7	6.0	0.1
18	3	8588	8954	8771.0	4	4.5	4.3	0.0
18	4	9503	8518	9010.5	11	9	10.0	0.1
18	5	8522	8182	8352.0	5	5	5.0	0.1
18	6	7566	7789	7677.5	5	5	5.0	0.1
18	7	7894	7806	7850.0	6.5	5.5	6.0	0.1
18	8	2787	4719	3753.0	9.5	8	8.8	0.2
18	9	5993	6021	6007.0	6.5	5.5	6.0	0.1
18	10	9496.5	10450	9973.3	17	15	16.0	0.2
18	11	1109	1107	1108.0	9	3	6.0	0.5
18	13	7227	6861	7044.0	4	5	4.5	0.1
18	14	6672	6497.5	6584.8	15	13	14.0	0.2
18	15	709	512	610.5	36	45	40.5	6.6
18	17	8248	7868	8058.0	7.5	7	7.3	0.1
18	18	9059	9941	9500.0	5	5	5.0	0.1
18	19	3969.5	3953	3961.3	12	12	12.0	0.3

18	20	7712	7792	7752.0	9	14	11.5	0.1
18	21	3483	3741.5	3612.3	8.5	15	11.8	0.3
19	2	1624.5	1766	1695.3	15	15	15.0	0.9
19	3	1955.5	1679	1817.3	1.5	2	1.8	0.1
19	4	1749	1670	1709.5	5	7	6.0	0.4
19	5	1520	1453.5	1486.8	3	2.5	2.8	0.2
19	9	533	564	548.5	21	12	16.5	3.0
19	10	993.5	1002	997.8	616	630	623.0	62.4
19	13	1105	1299	1202.0	2	11	6.5	0.5
19	14	1149.5	921	1035.3	8	6.5	7.3	0.7
19	17	1352	1086.5	1219.3	2	2	2.0	0.2
19	18	1539.5	1611	1575.3	1.5	3	2.3	0.1
20	2	5003	5473.5	5238.3	13.5	23	18.3	0.3
20	3	5179.5	4843	5011.3	2	2	2.0	0.0
20	4	4508	5147	4827.5	8	7	7.5	0.2
20	5	5555	4314	4934.5	3	2	2.5	0.1
20	6	1708	1677.5	1692.8	515	547	531.0	31.4
20	7	1788	1995	1891.5	521	521	521.0	27.5
20	8	958	969	963.5	307	298.5	302.8	31.4
20	9	2956	2567	2761.5	18	6	12.0	0.4
20	10	3028.5	3083	3055.8	1287	982	1134.5	37.1
20	13	4731	4870	4800.5	5	4.5	4.8	0.1
20	14	3743	3432	3587.5	11	12	11.5	0.3
20	17	4538	4324.5	4431.3	4	4	4.0	0.1
20	18	5025	5399	5212.0	2	4	3.0	0.1
20	19	1467	1205	1336.0	236	126	181.0	13.5
20	20	1753	1712	1732.5	318.5	384	351.3	20.3
20	21	1070	1109	1089.5	326	387	356.5	32.7
21	2	2051.5	2337	2194.3	25	29.5	27.3	1.2
21	3	2096	1847	1971.5	3	2	2.5	0.1
21	4	2018	2220	2119.0	7	6	6.5	0.3
21	5	1852	1769	1810.5	3	3	3.0	0.2
21	6	427	504	465.5	325	202.5	263.8	56.7
21	7	873.5	1060	966.8	395	547	471.0	48.7
21	9	898.5	636	767.3	17	14.5	15.8	2.1
21	10	841.5	886.5	864.0	526.5	472	499.3	57.8
21	13	1826	1651	1738.5	4	3	3.5	0.2
21	14	1241.5	1213.5	1227.5	7	6	6.5	0.5
21	17	1341	1352	1346.5	3.5	4	3.8	0.3
21	18	1657.5	1504	1580.8	3	2	2.5	0.2
21	20	779	522	650.5	280	331	305.5	47.0

Table S5. Anti-C clones on toxins

Captor	Tracer	C MFI 1	C MFI 2	C mMFI	Non-C MFI 1	Non-C MFI 2	Non-C mMFI	% x-reactivity
1	1	1403	1537	1470.0	4	3	3.5	0.2
1	2	1546	1393	1469.5	9	5	7.0	0.5
1	3	779.5	1244	1011.8	6	6	6.0	0.6
1	4	871	878.5	874.8	6	4	5.0	0.6
1	5	1038	781	909.5	9.5	9.5	9.5	1.0
1	6	5409	5688	5548.5	4	7.5	5.8	0.1
1	7	5445	4919	5182.0	5	3	4.0	0.1
1	8	1208.5	1067	1137.8	4	6	5.0	0.4
1	9	683.5	839	761.3	10	7.5	8.8	1.1
1	10	5221	5162	5191.5	10	13	11.5	0.2
1	11	5938	6205	6071.5	3	3	3.0	0.0
1	12	1058	2860	1959.0	9	9	9.0	0.5
1	15	5582	5288	5435.0	8	6	7.0	0.1
1	16	1471.5	1047	1259.3	9.5	5	7.3	0.6
1	18	1556	1394	1475.0	5	8	6.5	0.4
1	20	1350	1434	1392.0	4	6	5.0	0.4
1	21	1740.5	1394.5	1567.5	12.5	12	12.3	0.8
1	22	891.5	1104	997.8	7	7	7.0	0.7
1	23	850	854	852.0	4	3	3.5	0.4
1	24	7070.5	6948	7009.3	10	6	8.0	0.1
1	25	4503.5	3950	4226.8	6	10	8.0	0.2
1	26	1339	920	1129.5	6	6	6.0	0.5
3	4	443.5	575	509.3	83	8	45.5	8.9
3	5	535	527	531.0	357	392	374.5	70.5
3	8	435	582.5	508.8	341	331	336.0	66.0
3	21	571	775	673.0	1057	965	1011.0	150.2
4	5	646	632	639.0	23	20.5	21.8	3.4
4	8	582	551	566.5	15	16	15.5	2.7
4	9	732	784	758.0	29	33.5	31.3	4.1
4	10	412.5	545	478.8	27	22	24.5	5.1
4	16	549	376.5	462.8	9	13	11.0	2.4
4	18	512	577	544.5	13	12	12.5	2.3
4	21	983	1349	1166.0	31.5	31	31.3	2.7
4	25	530	599	564.5	24	28	26.0	4.6
6	1	3899	3992	3945.5	4	4	4.0	0.1
6	2	3130	2528	2829.0	5	4	4.5	0.2
6	6	2507	2789	2648.0	1.5	3	2.3	0.1
6	11	2078.5	2311	2194.8	3	2	2.5	0.1
6	12	63	656.5	359.8	9.5	9	9.3	2.6
6	15	1009	998	1003.5	5	5	5.0	0.5
6	24	2387.5	2059	2223.3	5	5	5.0	0.2
6	25	612	637.5	624.8	6	7	6.5	1.0
7	4	449	590	519.5	172	3	87.5	16.8
7	5	620.5	705	662.8	692	735.5	713.8	107.7

7	8	724	717	720.5	597	556	576.5	80.0
7	9	781	868	824.5	1384	1226	1305.0	158.3
7	10	521	616	568.5	1192	1341.5	1266.8	222.8
7	15	549	476	512.5	898.5	520	709.3	138.4
7	16	585	581	583.0	875	936	905.5	155.3
7	18	583.5	747	665.3	1120	961	1040.5	156.4
7	21	1134	1679	1406.5	1673.5	1403	1538.3	109.4
7	25	679	670.5	674.8	1024	1105.5	1064.8	157.8
9	21	76	126	101.0	601	362.5	481.8	477.0
10	1	3182	3114	3148.0	13	14	13.5	0.4
10	2	2601	1804	2202.5	15	13	14.0	0.6
10	6	2118	1993	2055.5	10	7	8.5	0.4
10	11	1728	1747	1737.5	8	6	7.0	0.4
10	15	624	602.5	613.3	10.5	11	10.8	1.8
10	24	1571	1267	1419.0	10	13	11.5	0.8
11	24	583	573	578.0	10	8	9.0	1.6
15	1	2199	2223	2211.0	27	41	34.0	1.5
15	2	1828.5	1189	1508.8	1428.5	247	837.8	55.5
15	3	579	814	696.5	2788.5	2592.5	2690.5	386.3
15	4	1053	1458.5	1255.8	873.5	43.5	458.5	36.5
15	5	1302	1398	1350.0	2428.5	2654.5	2541.5	188.3
15	6	1375.5	2155.5	1765.5	403	1321	862.0	48.8
15	7	1512	1712	1612.0	1006	1152	1079.0	66.9
15	8	1285.5	1282	1283.8	2618.5	2264	2441.3	190.2
15	9	1048	1249	1148.5	3388	3868.5	3628.3	315.9
15	10	1330.5	1411	1370.8	3269	3448	3358.5	245.0
15	11	2068	2176	2122.0	975	610.5	792.8	37.4
15	12	722	1089.5	905.8	2667	2791	2729.0	301.3
15	15	702.5	579	640.8	2308	2531	2419.5	377.6
15	16	1255.5	1056	1155.8	3130	3464	3297.0	285.3
15	18	1123.5	1047	1085.3	3568	3487	3527.5	325.0
15	21	1860	1776	1818.0	5170.5	5191.5	5181.0	285.0
15	22	599	627	613.0	2383	2356	2369.5	386.5
15	23	650.5	593.5	622.0	978	1015	996.5	160.2
15	24	436.5	436	436.3	586	651.5	618.8	141.8
15	25	692	683	687.5	2699	2536	2617.5	380.7
16	2	102	32	67.0	608	73	340.5	508.2
16	3	111	161	136.0	1569	1468	1518.5	1116.5
16	5	261	222	241.5	1319	1565	1442.0	597.1
16	6	112	137.5	124.8	151.5	601.5	376.5	301.8
16	8	197	213.5	205.3	1350.5	1290	1320.3	643.2
16	9	272	268	270.0	1941	1925	1933.0	715.9
16	10	171	196	183.5	2070.5	1954	2012.3	1096.6
16	12	137	125	131.0	1484	1546	1515.0	1156.5
16	15	173	147	160.0	1190	1293	1241.5	775.9
16	16	135.5	184	159.8	1485	1369	1427.0	893.3
16	18	160	196	178.0	1657	1630.5	1643.8	923.5
16	21	263	650	456.5	2091.5	2072.5	2082.0	456.1

16	22	122	127.5	124.8	1252	1453.5	1352.8	1084.4
16	25	167	171	169.0	1343.5	1556	1449.8	857.8
18	2	239.5	74	156.8	1558.5	4	781.3	498.4
18	3	304.5	424.5	364.5	3224	2943	3083.5	846.0
18	4	421	455.5	438.3	1091.5	21	556.3	126.9
18	5	590	597.5	593.8	2815.5	2925	2870.3	483.4
18	6	299	297	298.0	451	1531	991.0	332.6
18	7	334	369	351.5	949	848.5	898.8	255.7
18	8	524	529	526.5	2826	2715.5	2770.8	526.3
18	9	593	662	627.5	3952	4376.5	4164.3	663.6
18	10	376	429	402.5	3956	3939.5	3947.8	980.8
18	11	208	240.5	224.3	1098.5	841	969.8	432.4
18	12	314.5	275.5	295.0	3411	3326	3368.5	1141.9
18	15	372.5	375	373.8	2767	3057	2912.0	779.1
18	16	456	418	437.0	3335	3329.5	3332.3	762.5
18	18	434	430.5	432.3	3675	3746	3710.5	858.4
18	21	804.5	1208	1006.3	4702	4290	4496.0	446.8
18	22	295	362	328.5	3563	3255	3409.0	1037.7
18	23	218.5	200	209.3	1021	1025	1023.0	488.9
18	24	208.5	184	196.3	929	831	880.0	448.4
18	25	472	472	472.0	3056.5	3378	3217.3	681.6
21	2	216	62	139.0	916.5	126.5	521.5	375.2
21	3	288	353	320.5	2009	2002	2005.5	625.7
21	4	397	446	421.5	739.5	17	378.3	89.7
21	5	500	562	531.0	1945	2108	2026.5	381.6
21	6	230	269	249.5	325	1061	693.0	277.8
21	7	356	329	342.5	722.5	765	743.8	217.2
21	8	480	471	475.5	1990	1970	1980.0	416.4
21	9	514	587	550.5	2984.5	2883	2933.8	532.9
21	10	382.5	433	407.8	2652	2748	2700.0	662.2
21	11	174	219	196.5	632.5	536	584.3	297.3
21	12	281	335	308.0	2286	2236	2261.0	734.1
21	15	327	290	308.5	1825	1949	1887.0	611.7
21	16	391	432	411.5	2370	2549.5	2459.8	597.8
21	18	347.5	399	373.3	2649	2708	2678.5	717.6
21	21	763	1012	887.5	3646.5	3497	3571.8	402.5
21	22	256	263	259.5	2045.5	2217	2131.3	821.3
21	23	168.5	165	166.8	665	625.5	645.3	387.0
21	24	156	199	177.5	546	492.5	519.3	292.5
21	25	466	422	444.0	2458	2245	2351.5	529.6
22	3	228	293	260.5	700	577.5	638.8	245.2
22	4	610.5	627.5	619.0	176.5	12	94.3	15.2
22	5	753	703.5	728.3	834.5	829	831.8	114.2
22	7	461	555	508.0	562.5	401.5	482.0	94.9
22	8	494	635	564.5	996	901	948.5	168.0
22	9	527	528.5	527.8	1335	1334	1334.5	252.9
22	10	472.5	473.5	473.0	1465	1266	1365.5	288.7
22	12	310	288	299.0	877	719	798.0	266.9

22	15	224	189	206.5	653.5	794	723.8	350.5
22	16	379	272	325.5	1148.5	1494	1321.3	405.9
22	18	340	332.5	336.3	1164.5	1068.5	1116.5	332.0
22	21	805.5	1049	927.3	1972	2040	2006.0	216.3
22	22	143	179	161.0	655	639	647.0	401.9
22	23	451	406	428.5	568.5	462	515.3	120.2
22	25	259	280	269.5	780	771	775.5	287.8
23	3	272	339	305.5	711	683	697.0	228.2
23	5	563	567	565.0	859	914	886.5	156.9
23	8	489	561	525.0	795	718.5	756.8	144.1
23	9	540	519	529.5	956	1058.5	1007.3	190.2
23	10	347	397	372.0	1175	980.5	1077.8	289.7
23	12	363.5	301	332.3	873	697.5	785.3	236.3
23	15	371.5	370.5	371.0	773	751.5	762.3	205.5
23	16	582	479	530.5	1040	1127	1083.5	204.2
23	18	598	597	597.5	1145.5	1153	1149.3	192.3
23	21	994.5	1002	998.3	1713	1645	1679.0	168.2
23	22	309	301.5	305.3	826	928	877.0	287.3
23	25	438	514.5	476.3	942	906	924.0	194.0
24	1	5206	4617	4911.5	14	18	16.0	0.3
24	2	4281	3591	3936.0	17	19.5	18.3	0.5
24	6	4939	4297	4618.0	12	19	15.5	0.3
24	7	2502.5	2574	2538.3	16	15	15.5	0.6
24	10	2172.5	1900	2036.3	31.5	32.5	32.0	1.6
24	11	4467	4749	4608.0	16	8	12.0	0.3
24	12	188.5	1291	739.8	30	18.5	24.3	3.3
25	9	273.5	362	317.8	657	609	633.0	199.2
25	10	343	280.5	311.8	682.5	706	694.3	222.7
25	21	436	584.5	510.3	822	670.5	746.3	146.3

Table S6. Anti-C clones on complexes

Captor	Tracer	Cc MFI 1	Cc MFI 2	Cc mMFI	Non-Cc MFI 1	Non-Cc MFI 2	Non-Cc mMFI	% x-reactivity
1	6	2738	2252	2495.0	4	5	4.5	0.2
1	7	2434	148	1291.0	2.5	4	3.3	0.3
1	10	2233	2090.5	2161.8	4	2	3.0	0.1
1	11	2360	1690	2025.0	3	4	3.5	0.2
1	15	2562.5	2756	2659.3	5.5	5	5.3	0.2
1	24	3408.5	4124	3766.3	4	2.5	3.3	0.1
1	25	1550	1788	1669.0	9	9	9.0	0.5
3	3	3029	3200	3114.5	361	401.5	381.3	12.2
3	4	2914	2875	2894.5	7	94	50.5	1.7
3	5	2132	2229	2180.5	486	442	464.0	21.3
3	6	2401	338	1369.5	4	4.5	4.3	0.3
3	7	2574.5	2300	2437.3	346	306	326.0	13.4
3	8	3843	2662.5	3252.8	289	332	310.5	9.5
3	9	3597	4287	3942.0	877.5	798	837.8	21.3
3	10	2437	2295.5	2366.3	6	3	4.5	0.2
3	11	1760.5	1969	1864.8	73	107	90.0	4.8
3	12	2740	2943.5	2841.8	483	534	508.5	17.9
3	15	2566	2844.5	2705.3	282.5	336	309.3	11.4
3	16	3235	3742	3488.5	359	350	354.5	10.2
3	18	3821.5	3720.5	3771.0	363	275	319.0	8.5
3	20	605	505.5	555.3	33	35.5	34.3	6.2
3	21	5682.5	5188	5435.3	768	988.5	878.3	16.2
3	22	2402	2511	2456.5	232.5	210.5	221.5	9.0
3	23	1901	1946.5	1923.8	125.5	150	137.8	7.2
3	24	1279.5	1534.5	1407.0	43	41	42.0	3.0
3	25	3845	3349	3597.0	258	211	234.5	6.5
4	3	2727	2748	2737.5	12	15	13.5	0.5
4	4	1116.5	1074	1095.3	2	6	4.0	0.4
4	5	2114	2029.5	2071.8	27	31	29.0	1.4
4	6	2028.5	134.5	1081.5	2	2	2.0	0.2
4	7	1342	1142	1242.0	9	11	10.0	0.8
4	8	3127	2758	2942.5	21	18	19.5	0.7
4	9	3249.5	3534	3391.8	37	28	32.5	1.0
4	10	1914	1619	1766.5	1	1	1.0	0.1
4	11	1532	1916	1724.0	7	7	7.0	0.4
4	12	2483	2684	2583.5	28.5	31	29.8	1.2
4	15	2208.5	2359.5	2284.0	18	15	16.5	0.7
4	16	2525	2284	2404.5	10	10	10.0	0.4
4	18	2766	2970	2868.0	8	8	8.0	0.3
4	21	3876	4085	3980.5	21	23	22.0	0.6
4	22	2388	2329	2358.5	9.5	8	8.8	0.4
4	23	1056	1064	1060.0	5	5	5.0	0.5
4	24	1231	1370.5	1300.8	5	5	5.0	0.4
4	25	3294	3127	3210.5	24	17	20.5	0.6

5	3	1176	930	1053.0	56.5	56	56.3	5.3
5	4	1174	1153.5	1163.8	1	14	7.5	0.6
5	5	762.5	729	745.8	92.5	77	84.8	11.4
5	6	1040.5	134.5	587.5	2.5	2	2.3	0.4
5	7	1154.5	1003	1078.8	78	70	74.0	6.9
5	8	1468	846	1157.0	44.5	42	43.3	3.7
5	9	1571	1623	1597.0	191	161.5	176.3	11.0
5	10	1046	812	929.0	4	3	3.5	0.4
5	11	591.5	643	617.3	13.5	17	15.3	2.5
5	12	1018	1191.5	1104.8	62	72.5	67.3	6.1
5	15	933	1277	1105.0	47	55	51.0	4.6
5	16	1137	983.5	1060.3	39	51.5	45.3	4.3
5	18	1252.5	1328	1290.3	37	44	40.5	3.1
5	21	1641.5	1951.5	1796.5	111	159	135.0	7.5
5	22	841	901.5	871.3	29.5	36	32.8	3.8
5	23	827	848.5	837.8	22	20	21.0	2.5
5	25	1412	1558	1485.0	45	35	40.0	2.7
6	1	2139	2148	2143.5	3	3	3.0	0.1
6	2	1630.5	1261	1445.8	3	3	3.0	0.2
6	6	1463	1465.5	1464.3	2	2	2.0	0.1
6	11	970	1050	1010.0	2	2.5	2.3	0.2
6	24	971	1073	1022.0	2	4	3.0	0.3
7	3	2598.5	2389	2493.8	881	943	912.0	36.6
7	4	1112.5	1107	1109.8	3	263	133.0	12.0
7	5	2029	1972	2000.5	1137	1081.5	1109.3	55.4
7	6	2224	147	1185.5	2	1	1.5	0.1
7	7	1351	1071	1211.0	426	394	410.0	33.9
7	8	3394	2581	2987.5	867	1107	987.0	33.0
7	9	3281	3557.5	3419.3	1696	1700.5	1698.3	49.7
7	10	2087	1695	1891.0	3	4	3.5	0.2
7	11	1622	1784	1703.0	256	328	292.0	17.1
7	12	2501	2633	2567.0	1303.5	1320	1311.8	51.1
7	15	2308	2532	2420.0	909	964	936.5	38.7
7	16	2563	2977	2770.0	958.5	1082.5	1020.5	36.8
7	18	3084	2902	2993.0	1061	1070	1065.5	35.6
7	21	4243	3803.5	4023.3	1681	1858	1769.5	44.0
7	22	2548	2782	2665.0	846	850.5	848.3	31.8
7	23	1138	1102.5	1120.3	232.5	245	238.8	21.3
7	24	1153	1301	1227.0	197	198	197.5	16.1
7	25	3427.5	3667	3547.3	1120	968	1044.0	29.4
8	3	972	841	906.5	57	51	54.0	6.0
8	4	893	940	916.5	2	18	10.0	1.1
8	5	611	612.5	611.8	72	82	77.0	12.6
8	6	703	96	399.5	1	1	1.0	0.3
8	7	845	785	815.0	63.5	56.5	60.0	7.4
8	8	1290	763.5	1026.8	44	44	44.0	4.3
8	9	1022	924.5	973.3	80	100	90.0	9.2
8	10	809.5	611.5	710.5	2	2	2.0	0.3

8	11	445.5	556	500.8	14	15	14.5	2.9
8	12	718.5	850	784.3	69	76.5	72.8	9.3
8	15	912	881	896.5	51	44	47.5	5.3
8	16	701.5	748	724.8	33.5	50	41.8	5.8
8	18	953	909	931.0	40	39	39.5	4.2
8	21	1845.5	1455.5	1650.5	118.5	119	118.8	7.2
8	22	638	888	763.0	29	28	28.5	3.7
8	23	469.5	513	491.3	19	18	18.5	3.8
8	25	1281	1318	1299.5	45.5	57	51.3	3.9
9	8	637	325	481.0	56	55	55.5	11.5
9	16	482	570	526.0	87.5	106.5	97.0	18.4
9	18	578	560	569.0	103	92	97.5	17.1
9	21	1149	1020	1084.5	273	305	289.0	26.6
9	25	855	801	828.0	127	119	123.0	14.9
10	1	1528	1506	1517.0	11	9	10.0	0.7
10	2	1142	905	1023.5	10	12	11.0	1.1
10	6	887	969.5	928.3	7	7	7.0	0.8
10	11	584.5	584	584.3	7	9	8.0	1.4
10	24	490	572	531.0	8	11	9.5	1.8
12	3	634	605.5	619.8	116.5	84	100.3	16.2
12	4	753	696	724.5	5	27	16.0	2.2
12	5	513	487	500.0	158	170	164.0	32.8
12	6	602.5	83	342.8	2	2	2.0	0.6
12	7	898	740.5	819.3	137	131	134.0	16.4
12	8	1216	493.5	854.8	113	98	105.5	12.3
12	9	936	970	953.0	252.5	207	229.8	24.1
12	10	548.5	463.5	506.0	2	2.5	2.3	0.4
12	12	572	689.5	630.8	127	119	123.0	19.5
12	15	652	661.5	656.8	81.5	96	88.8	13.5
12	16	623	735	679.0	81	128	104.5	15.4
12	18	728	595	661.5	104.5	97.5	101.0	15.3
12	21	1114.5	1209	1161.8	233	245	239.0	20.6
12	22	573	509.5	541.3	63	65	64.0	11.8
12	25	931	892	911.5	78	114.5	96.3	10.6
15	1	1014	954	984.0	32	31	31.5	3.2
15	2	842	650.5	746.3	180	185	182.5	24.5
15	3	4257	4193.5	4225.3	2754	2854	2804.0	66.4
15	4	3236	3145	3190.5	41	1089	565.0	17.7
15	5	3239	3285	3262.0	2661	2737.5	2699.3	82.7
15	6	3756	948	2352.0	32	31	31.5	1.3
15	7	3243	3206	3224.5	1139	1128.5	1133.8	35.2
15	8	5283	3767	4525.0	2463	2504	2483.5	54.9
15	9	5148	5226.5	5187.3	3912	3333.5	3622.8	69.8
15	10	3260	2793	3026.5	37	31	34.0	1.1
15	11	2883	3238	3060.5	821	1177	999.0	32.6
15	12	3888.5	4210	4049.3	2810	2755	2782.5	68.7
15	15	3569	3535	3552.0	2122.5	1955	2038.8	57.4
15	16	4389	4935.5	4662.3	2656.5	2836	2746.3	58.9

15	18	5251.5	5304.5	5278.0	2986.5	2722.5	2854.5	54.1
15	20	623	580	601.5	174.5	196	185.3	30.8
15	21	6740.5	7239	6989.8	4411	4427	4419.0	63.2
15	22	3618.5	3714	3666.3	1943	1997.5	1970.3	53.7
15	23	2078	2189	2133.5	777	841.5	809.3	37.9
15	24	2024.5	2227	2125.8	523	599.5	561.3	26.4
15	25	5048.5	5067	5057.8	2257	2359	2308.0	45.6
16	3	1201.5	1036	1118.8	1222.5	899	1060.8	94.8
16	4	518	531	524.5	7	390	198.5	37.8
16	5	751	835	793.0	1238	1251	1244.5	156.9
16	6	616	77.5	346.8	5.5	7	6.3	1.8
16	7	605	577	591.0	370.5	298	334.3	56.6
16	8	1237	1048	1142.5	1136	1182	1159.0	101.4
16	9	1474	1322	1398.0	1615	1499	1557.0	111.4
16	10	835.5	573	704.3	5.5	8	6.8	1.0
16	11	564	632	598.0	239	410	324.5	54.3
16	12	796	914	855.0	1208.5	1251	1229.8	143.8
16	15	960	913	936.5	859.5	868.5	864.0	92.3
16	16	765	802	783.5	835	1050	942.5	120.3
16	18	917	847.5	882.3	1018	1025	1021.5	115.8
16	21	1246.5	1259	1252.8	1337	1457	1397.0	111.5
16	22	928	1287	1107.5	968	965	966.5	87.3
16	25	1425	1227.5	1326.3	1345.5	1112.5	1229.0	92.7
18	3	2571	2259	2415.0	2806.5	3022	2914.3	120.7
18	4	1244	1226	1235.0	18	1127	572.5	46.4
18	5	2012	1992	2002.0	2804.5	2868.5	2836.5	141.7
18	6	1999	210	1104.5	11	9	10.0	0.9
18	7	1540	1210	1375.0	863.5	961	912.3	66.3
18	8	2830.5	2402	2616.3	2769	2858	2813.5	107.5
18	9	3482	3302.5	3392.3	3876	3942	3909.0	115.2
18	10	1823	1694	1758.5	22	12.5	17.3	1.0
18	11	1461	1627.5	1544.3	905	1157	1031.0	66.8
18	12	2154	2442	2298.0	3112	2974	3043.0	132.4
18	15	2039	2148	2093.5	2398	2223	2310.5	110.4
18	16	1981	2179	2080.0	2397	2543	2470.0	118.8
18	18	2077	2186	2131.5	2903.5	2859	2881.3	135.2
18	21	2933	2811	2872.0	3736	4075.5	3905.8	136.0
18	22	2438	2484	2461.0	2431.5	2514.5	2473.0	100.5
18	23	857	811	834.0	671	754	712.5	85.4
18	24	1250	1232	1241.0	648.5	684.5	666.5	53.7
18	25	3303.5	3057	3180.3	2824	2874	2849.0	89.6
20	3	523	593	558.0	96	99	97.5	17.5
20	4	512	558	535.0	4.5	28	16.3	3.0
20	8	684.5	420.5	552.5	75.5	68	71.8	13.0
20	9	519	528.5	523.8	159	148.5	153.8	29.4
20	21	760.5	722	741.3	147	151	149.0	20.1
20	25	704	563	633.5	97.5	61	79.3	12.5
21	3	2117	1752	1934.5	1791	1989	1890.0	97.7

21	4	1061	1171	1116.0	15	739	377.0	33.8
21	5	1501	1445.5	1473.3	1966	1854	1910.0	129.6
21	6	1520	169	844.5	13	14	13.5	1.6
21	7	1215	1049	1132.0	685	704	694.5	61.4
21	8	2427	1620.5	2023.8	1765.5	1831	1798.3	88.9
21	9	2481.5	2565.5	2523.5	2688.5	2578.5	2633.5	104.4
21	10	1537	1277	1407.0	15	13	14.0	1.0
21	11	1117	1308	1212.5	519	736	627.5	51.8
21	12	1759	1781	1770.0	2040	2247	2143.5	121.1
21	15	1737	1741	1739.0	1466	1684.5	1575.3	90.6
21	16	1659	1563.5	1611.3	1623	1940	1781.5	110.6
21	18	1703	1696.5	1699.8	1982	1865	1923.5	113.2
21	21	2439	2570	2504.5	2706	2750	2728.0	108.9
21	22	1774	1749	1761.5	1762	1635	1698.5	96.4
21	23	596.5	570	583.3	391	450	420.5	72.1
21	24	848.5	823	835.8	398.5	475	436.8	52.3
21	25	2472	2332.5	2402.3	2098	1914	2006.0	83.5
22	3	3633	3449	3541.0	950	801	875.5	24.7
22	4	3141	2901.5	3021.3	11	309	160.0	5.3
22	5	2657	2387	2522.0	1121	1004	1062.5	42.1
22	6	2828	409	1618.5	5	5	5.0	0.3
22	7	2994.5	3034.5	3014.5	761	935	848.0	28.1
22	8	4564	2848	3706.0	694	746	720.0	19.4
22	9	4742	4612	4677.0	1782	1914	1848.0	39.5
22	10	3164	2764	2964.0	7	8	7.5	0.3
22	11	1928	2407	2167.5	216	287	251.5	11.6
22	12	3333	3452	3392.5	1083	1175.5	1129.3	33.3
22	15	2949	3076.5	3012.8	563.5	692	627.8	20.8
22	16	3897	4474	4185.5	810.5	967	888.8	21.2
22	18	4255	4410	4332.5	880	948.5	914.3	21.1
22	20	673	631	652.0	66	84	75.0	11.5
22	21	6681	6958	6819.5	1765.5	1786	1775.8	26.0
22	22	2825.5	2899	2862.3	550	584.5	567.3	19.8
22	23	2174	2306	2240.0	356	320	338.0	15.1
22	24	1717	1854	1785.5	89	119	104.0	5.8
22	25	4333.5	4145	4239.3	611	489	550.0	13.0
23	3	1177	1102	1139.5	465	455	460.0	40.4
23	4	539	612	575.5	13	183.5	98.3	17.1
23	5	959	903	931.0	613	692	652.5	70.1
23	6	1049	70	559.5	11	18.5	14.8	2.6
23	7	586	515	550.5	167	190	178.5	32.4
23	8	1566	1155	1360.5	574	580.5	577.3	42.4
23	9	1225	1508.5	1366.8	806	707	756.5	55.4
23	10	772	604	688.0	13	12.5	12.8	1.9
23	11	629	814.5	721.8	139	167.5	153.3	21.2
23	12	1127	962	1044.5	650	638	644.0	61.7
23	15	1115	1169	1142.0	512	488.5	500.3	43.8
23	16	1081	913.5	997.3	508	700	604.0	60.6

23	18	1284	1232	1258.0	671.5	661	666.3	53.0
23	21	1765	1866	1815.5	919.5	996	957.8	52.8
23	22	1045	938	991.5	533	465.5	499.3	50.4
23	24	571	649	610.0	116.5	106	111.3	18.2
23	25	1556	1390	1473.0	634	579	606.5	41.2
24	1	3497	3280	3388.5	14.5	16	15.3	0.5
24	2	2898	2427	2662.5	9.5	15	12.3	0.5
24	6	3310	3565.5	3437.8	13.5	6	9.8	0.3
24	7	1872	136.5	1004.3	17	14	15.5	1.5
24	10	1065	1069	1067.0	10	13	11.5	1.1
24	11	2927	3067.5	2997.3	9	11	10.0	0.3
25	3	2313	2263	2288.0	274	311	292.5	12.8
25	4	1812.5	1863	1837.8	4	55	29.5	1.6
25	5	1471.5	1822	1646.8	263	386	324.5	19.7
25	6	1753	276	1014.5	2	2	2.0	0.2
25	7	2032	1572	1802.0	212	210	211.0	11.7
25	8	3059	1842.5	2450.8	226.5	203	214.8	8.8
25	9	3176	3620.5	3398.3	955.5	802.5	879.0	25.9
25	10	2118.5	1590	1854.3	4	7	5.5	0.3
25	11	1337	1458	1397.5	57.5	72	64.8	4.6
25	12	2015	2500	2257.5	349	447	398.0	17.6
25	15	1836	2044	1940.0	212.5	213	212.8	11.0
25	16	2948.5	2529.5	2739.0	289	266	277.5	10.1
25	18	3014	3286	3150.0	300.5	238	269.3	8.5
25	21	5106	4089	4597.5	737.5	726	731.8	15.9
25	22	2216	1933.5	2074.8	144	154.5	149.3	7.2
25	23	1376	1374	1375.0	73.5	89	81.3	5.9
25	24	960	861	910.5	16	28	22.0	2.4
25	25	2522	2748	2635.0	213	171.5	192.3	7.3

Table S7. Anti-D clones on toxins

Captor	Tracer	D MFI 1	D MFI 2	D mMFI	Non-D MFI 1	Non-D MFI 2	Non-D mMFI	% x-reactivity
1	15	640	665	652.5	12	14	13.0	2.0
1	16	710	654.5	682.3	9	9	9.0	1.3
1	17	1076	950	1013.0	21.5	23	22.3	2.2
1	22	765	756	760.5	18	15	16.5	2.2
1	27	722.5	794	758.3	18.5	16	17.3	2.3
1	28	534	548	541.0	10	12	11.0	2.0
17	15	581	661	621.0	277.5	296	286.8	46.2
17	17	599	559.5	579.3	106	106	106.0	18.3
17	22	529.5	598	563.8	154	139	146.5	26.0
22	1	910	876	893.0	49	44	46.5	5.2
22	3	614.5	673	643.8	38	31	34.5	5.4
22	10	1293	1252.5	1272.8	127.5	121	124.3	9.8
22	11	1099	1115	1107.0	57	61.5	59.3	5.4
22	14	985	903.5	944.3	20	19	19.5	2.1
22	15	2021	2024.5	2022.8	355	344	349.5	17.3
22	16	1440.5	1345	1392.8	32	32.5	32.3	2.3
22	17	2140	2142	2141.0	259.5	235.5	247.5	11.6
22	22	1353.5	1324	1338.8	143.5	149	146.3	10.9
22	27	1214	1259	1236.5	185.5	242.5	214.0	17.3
22	28	924	962.5	943.3	108.5	115	111.8	11.8
22	32	1192	1217	1204.5	207	193	200.0	16.6
22	34	679	445	562.0	178.5	70	124.3	22.1
24	17	479	529.5	504.3	53	58	55.5	11.0
27	15	551	534.5	542.8	197.5	191.5	194.5	35.8
27	17	800	832	816.0	154	140	147.0	18.0
27	22	592	556.5	574.3	139.5	122	130.8	22.8
27	27	577	600	588.5	199	199.5	199.3	33.9
28	17	788	843.5	815.8	135	111.5	123.3	15.1
28	22	517	488	502.5	105.5	100	102.8	20.4
28	27	467	504.5	485.8	143	158.5	150.8	31.0
29	15	700.5	757	728.8	262	269	265.5	36.4
29	16	609.5	613	611.3	23	26	24.5	4.0
29	17	1328	1177	1252.5	202.5	181	191.8	15.3
29	22	784	818	801.0	170	156	163.0	20.3
29	27	703	696	699.5	210	263.5	236.8	33.8
29	28	535	542	538.5	143	160	151.5	28.1
29	32	743	765.5	754.3	237.5	225	231.3	30.7
31	17	665	681	673.0	111	108.5	109.8	16.3

Table S8. Anti-D clones on complexes

Captor	Tracer	Dc MFI 1	Dc MFI 2	Dc mMFI	Non-Dc MFI 1	Non-Dc MFI 2	Non-Dc mMFI	% x-reactivity
1	17	1217.5	1158	1187.8	16	14	15.0	1.3
1	22	848	763	805.5	10.5	11	10.8	1.3
1	27	739	688	713.5	11	12	11.5	1.6
1	28	528	519	523.5	10	12	11.0	2.1
11	17	632	659.5	645.8	16	14	15.0	2.3
16	17	673	620.5	646.8	84	88.5	86.3	13.3
17	17	849	809	829.0	196	190	193.0	23.3
17	22	596.5	561	578.8	353	292.5	322.8	55.8
17	32	403	527	465.0	403	374	388.5	83.5
22	16	1420.5	1265	1342.8	2	6	4	0.3
22	17	2643.5	2570	2606.8	851	949.5	900.3	34.5
22	22	1349	1419	1384.0	721	662.5	691.8	50.0
22	27	1342	1281	1311.5	768	765.5	766.8	58.5
22	28	972	997	984.5	510	572.5	541.3	55.0
22	32	1153	1290.5	1221.8	966.5	950	958.3	78.4
22	34	801	629	715.0	717	773.5	745.3	104.2
24	17	696.5	715.5	706.0	170	187	178.5	25.3
27	17	1061	986	1023.5	380.5	383	381.8	37.3
27	22	651	615	633.0	355	379	367.0	58.0
27	27	640	663	651.5	455	510	482.5	74.1
27	32	528	628	578.0	621	505	563.0	97.4
28	17	1018	982.5	1000.3	343	335	339.0	33.9
28	22	575	507	541.0	339.5	314.5	327.0	60.4
28	27	522	529	525.5	399.5	409	404.3	76.9
29	17	1621	1638	1629.5	578.5	591.5	585.0	35.9
29	22	933.5	886	909.8	532.5	487	509.8	56.0
29	27	839	795	817.0	660	622	641.0	78.5
29	28	657	586	621.5	424	429	426.5	68.6
29	32	722	859	790.5	804	747	775.5	98.1
31	17	886	905	895.5	347	324	335.5	37.5

Table S9. Anti-E clones on toxins

Captor	Tracer	E<br>MFI 1	E<br>MFI 2	E<br>mMFI	Non-E<br>MFI 1	Non-E<br>MFI 2	Non-E<br>mMFI	% x-reactivity
2	3	505	425	465.0	64.5	72	68.3	14.7
2	4	1624	2091	1857.5	15	14	14.5	0.8
2	7	412	512	462.0	16	12.5	14.3	3.1
2	8	1429	1396	1412.5	25	15	20.0	1.4
3	4	1405	1487	1446.0	2	2	2.0	0.1
3	8	962.5	1035	998.8	3	3	3.0	0.3
4	1	1289	1098	1193.5	1	3	2.0	0.2
4	2	1260	1236	1248.0	3	3.5	3.3	0.3
4	3	1106.5	1212	1159.3	3	2	2.5	0.2
4	4	538.5	523.5	531.0	2	2	2.0	0.4
4	7	1365	1325	1345.0	3.5	2	2.8	0.2
4	8	1413	1583	1498.0	4	2	3.0	0.2
6	3	560	472	516.0	6	15	10.5	2.0
6	4	1126	1106	1116.0	6	6	6.0	0.5
6	7	721	682.5	701.8	6	11.5	8.8	1.2
6	8	1136	1112.5	1124.3	5	3	4.0	0.4
7	4	3110	2994.5	3052.3	2	3.5	2.8	0.1
7	8	3079	2944.5	3011.8	5	4	4.5	0.1
8	1	4048.5	3759	3903.8	7	7	7.0	0.2
8	2	3482.5	3617	3549.8	11	14	12.5	0.4
8	3	3166	3228.5	3197.3	7.5	6	6.8	0.2
8	4	4644	4771	4707.5	5	6.5	5.8	0.1
8	5	1305	1277	1291.0	6	6	6.0	0.5
8	6	1610	1446	1528.0	8	7	7.5	0.5
8	7	4335	4232	4283.5	9	5	7.0	0.2
8	8	1736	1999	1867.5	10.5	10	10.3	0.5

Table S10. Anti-E clones on complexes

Captor	Tracer	Ec MFI 1	Ec MFI 2	Ec mMFI	Non-Ec MFI 1	Non-Ec MFI 2	Non-Ec mMFI	% x-reactivity
2	4	1104	1123	1113.5	17	11	14.0	1.3
2	8	657	629.5	643.3	16	12	14.0	2.2
3	4	1048	984	1016.0	1	1	1.0	0.1
3	8	485	524	504.5	3	4	3.5	0.7
4	1	652.5	666	659.3	2	2	2.0	0.3
4	2	665	638	651.5	3	1	2.0	0.3
4	3	490	599.5	544.8	1	2	1.5	0.3
4	7	621	837	729.0	3	3.5	3.3	0.4
4	8	732	838	785.0	3	3	3.0	0.4
7	4	2185.5	2436	2310.8	3	4	3.5	0.2
7	8	2175	2107	2141.0	3	4	3.5	0.2
8	1	2466.5	2529	2497.8	11	5	8.0	0.3
8	2	2078	1954.5	2016.3	10	7	8.5	0.4
8	3	2000.5	2139	2069.8	7.5	6	6.8	0.3
8	4	3333	3449	3391.0	6	8	7.0	0.2
8	5	607	768	687.5	9	6	7.5	1.1
8	6	663.5	754	708.8	7	8	7.5	1.1
8	7	2575	2840.5	2707.8	8	10	9.0	0.3
8	8	1054.5	999	1026.8	9	9	9.0	0.9

Table S11. Anti-F clones on toxins

Captor	Tracer	F MFI 1	F MFI 2	F mMFI	non-F MFI 1	non-F MFI 2	non-F mMFI	% x-reactivity
1	5	1673	1370	1521.5	5	13	9.0	0.6
1	8	803.5	627.5	715.5	2	7	4.5	0.6
1	9	923	1254	1088.5	6	3	4.5	0.4
3	5	3705	3741.5	3723.3	9	12	10.5	0.3
3	8	3153	3133	3143.0	11	10	10.5	0.3
3	9	5722	5087	5404.5	9	6.5	7.8	0.1
3	10	591	666.5	628.8	7	10	8.5	1.4
5	5	1944	1722	1833.0	9	6	7.5	0.4
5	8	1704	1865	1784.5	6.5	9	7.8	0.4
5	9	3780.5	4518	4149.3	4	3	3.5	0.1
5	10	622	535	578.5	4	4	4.0	0.7
7	5	3122	3078	3100.0	33	26	29.5	1.0
7	8	2308	2343	2325.5	35.5	28.5	32.0	1.4
7	9	2621	2785	2703.0	23.5	23	23.3	0.9
8	5	1740	1685	1712.5	8	10	9.0	0.5
8	9	2406	2356	2381.0	5	6	5.5	0.2
9	5	7109	6954.5	7031.8	4	5	4.5	0.1
9	7	561	490.5	525.8	1	2.5	1.8	0.3
9	8	4779	4824	4801.5	4	6	5.0	0.1
9	9	2563	3021	2792.0	2	4	3.0	0.1
10	5	529.5	583	556.3	5	4	4.5	0.8
10	9	703.5	774.5	739.0	1	2	1.5	0.2

Table S12. Anti-F clones on complexes

Captor	Tracer	Fc MFI 1	Fc MFI 2	Fc mMFI	non-Fc MFI 1	non-Fc MFI 2	non-Fc mMFI	% x-reactivity
3	5	3439	3116	3277.5	19	23	21.0	0.6
3	8	2513	1746	2129.5	10	13	11.5	0.5
3	9	4945	5285	5115.0	9	8	8.5	0.2
5	5	905.5	920	912.8	6	5.5	5.8	0.6
5	8	971	1084	1027.5	2	8	5.0	0.5
5	9	3360	3439	3399.5	4	5	4.5	0.1
7	5	1403	1338.5	1370.8	32	39.5	35.8	2.6
7	8	734	654	694.0	35	40	37.5	5.4
7	9	1064.5	849	956.8	43	32	37.5	3.9
8	5	1174	1178	1176.0	9	6	7.5	0.6
8	9	1720	1596	1658.0	4	4.5	4.3	0.3
9	5	6053	5827	5940.0	7	8	7.5	0.1
9	8	3394.5	3658	3526.3	5	5	5.0	0.1
9	9	2428	2250	2339.0	4	1	2.5	0.1

Table S13. Anti-G clones on toxins

Captor	Tracer	G MFI 1	G MFI 2	G mMFI	Non-G MFI 1	Non-G MFI 2	Non-G mMFI	% x-reactivity
1	2	840	672.5	756.3	87	88	87.5	11.6
1	3	1017.5	1048	1032.8	83.5	79	81.3	7.9
1	3	4224	3623.5	3923.8	7	5	6.0	0.2
1	11	1137	1166	1151.5	62	57	59.5	5.2
2	3	930.5	1043	986.8	5	5	5.0	0.5
2	18	1060	1170	1115.0	8	5	6.5	0.6
2	19	842	833	837.5	5	8	6.5	0.8
2	21	753.5	720.5	737.0	13.5	5	9.3	1.3
3	3	603	586	594.5	2	2.5	2.3	0.4
4	3	1255.5	1252.5	1254.0	173	134	153.5	12.2
4	11	774	810	792.0	117	143.5	130.3	16.4
4	15	350	503.5	426.8	1162	152	657.0	154.0
4	18	1031	1342	1186.5	118	134	126.0	10.6
4	19	992	1014	1003.0	141	135.5	138.3	13.8
4	21	897	976.5	936.8	88	129	108.5	11.6
4	20	645.5	680	662.8	101.5	107	104.3	15.7
4	26	532.5	469	500.8	162.5	132.5	147.5	29.5
7	11	560	553	556.5	20.5	24	22.3	4.0
7	15	940	889	914.5	33	36	34.5	3.8
7	26	1745	1841	1793.0	24	30	27.0	1.5
8	3	1164	1309	1236.5	6	6	6.0	0.5
11	11	627.5	619	623.3	45	52	48.5	7.8
15	3	1288.5	1338.5	1313.5	4	5	4.5	0.3
15	11	1156	938	1047.0	5	5.5	5.3	0.5
15	26	1377	1453	1415.0	4	6	5.0	0.4
18	2	3016	2877	2946.5	65	70	67.5	2.3
18	3	4320.5	4084	4202.3	73	64	68.5	1.6
18	4	806	800	803.0	76	54	65.0	8.1
18	11	2636	2515	2575.5	64	56	60.0	2.3
18	26	505.5	525.5	515.5	79.5	82	80.8	15.7
19	2	934	806	870.0	8	8	8.0	0.9
19	3	1336	1397	1366.5	10	13	11.5	0.8
19	11	754.5	640	697.3	8	7	7.5	1.1
21	2	4553	4390	4471.5	34	40	37.0	0.8
21	3	6829	6825.5	6827.3	34	39	36.5	0.5
21	4	1408	1417	1412.5	31	24	27.5	1.9
21	8	1580	1813.5	1696.8	35	40	37.5	2.2
21	11	4439	4680	4559.5	33.5	39	36.3	0.8
21	15	689	723	706.0	34	50	42.0	5.9
21	24	996	885	940.5	29	24	26.5	2.8
21	26	979.5	1081	1030.3	120	45	82.5	8.0
20	2	3209.5	3004	3106.8	2	2	2.0	0.1
20	3	4811	5204	5007.5	3	2	2.5	0.0
20	4	828	939	883.5	1	1	1.0	0.1

20	8	1139	1217	1178.0	2	1	1.5	0.1
20	11	2955	2963.5	2959.3	3	3	3.0	0.1
20	26	593	574	583.5	3	1	2.0	0.3
25	15	768	749	758.5	14	16	15.0	2.0
25	26	1047	1165.5	1106.3	12	13	12.5	1.1

Table S14. Anti-G clones on complexes

Captor	Tracer	Gc MFI 1	Gc MFI 2	Gc mMFI	Non-Gc MFI 1	Non-Gc MFI 2	Non-Gc mMFI	% x-reactivity
1	2	2893	2808	2850.5	73	67	70.0	2.5
1	3	3734	4354.5	4044.3	80.5	71	75.8	1.9
1	4	846	881	863.5	85	83	84.0	9.7
1	6	497.5	540	518.8	75	88	81.5	15.7
1	8	918	911.5	914.8	85	74	79.5	8.7
1	11	5075	4865	4970.0	63.5	66	64.8	1.3
1	12	570.5	555	562.8	61	70.5	65.8	11.7
2	1	955.5	1050	1002.8	4	5	4.5	0.4
2	2	2708	2415	2561.5	4	8	6.0	0.2
2	3	4701	4949	4825.0	6	6	6.0	0.1
2	4	881	881	881.0	8	3	5.5	0.6
2	8	855.5	989	922.3	4	6	5.0	0.5
2	11	4102	3874.5	3988.3	5.5	6	5.8	0.1
2	15	1037	1179	1108.0	7	7.5	7.3	0.7
2	18	1414	1368	1391.0	5.5	5.5	5.5	0.4
2	19	919	1053	986.0	5	5	5.0	0.5
2	21	967	1055	1011.0	6	5	5.5	0.5
2	20	606	617.5	611.8	5	7	6.0	1.0
2	26	1142.5	986	1064.3	5	5	5.0	0.5
3	1	550	658.5	604.3	2	2	2.0	0.3
3	2	1867	1501	1684.0	2	3	2.5	0.1
3	3	2823	3167.5	2995.3	3	2	2.5	0.1
3	4	495.5	535	515.3	1	2	1.5	0.3
3	8	609	573	591.0	2	1	1.5	0.3
3	11	2418	2204	2311.0	2	2	2.0	0.1
3	15	556	670	613.0	2	4	3.0	0.5
3	18	721	903	812.0	2	2	2.0	0.2
3	19	526.5	481.5	504.0	2	2	2.0	0.4
3	21	545	578	561.5	1.5	1	1.3	0.2
3	26	594	530	562.0	3	2.5	2.8	0.5
4	1	1238	1379.5	1308.8	155.5	196	175.8	13.4
4	2	2995	3290	3142.5	252.5	231	241.8	7.7
4	3	4486	4719	4602.5	158	198	178.0	3.9
4	4	1040.5	1194.5	1117.5	204	160	182.0	16.3
4	5	668	667	667.5	133.5	151	142.3	21.3
4	8	813	863	838.0	224	177	200.5	23.9
4	11	4143	3904	4023.5	132	151	141.5	3.5
4	15	1128	1102.5	1115.3	238	228	233.0	20.9
4	18	1583.5	1768.5	1676.0	213.5	246	229.8	13.7
4	19	1226	1265	1245.5	133	166	149.5	12.0
4	21	1172	1159.5	1165.8	223	217	220.0	18.9
4	20	904	970	937.0	235.5	185.5	210.5	22.5
4	24	627	738.5	682.8	244	220	232.0	34.0
4	26	1319	1516.5	1417.8	150.5	217	183.8	13.0
7	2	506	513.5	509.8	25	22	23.5	4.6

7	3	957	969	963.0	21	23	22.0	2.3
7	11	832.5	745.5	789.0	21	19	20.0	2.5
8	3	1686.5	2038	1862.3	7	7	7.0	0.4
8	11	1342	1380	1361.0	6	6	6.0	0.4
10	2	594.5	677	635.8	24.5	15	19.8	3.1
10	3	1027	1100.5	1063.8	20	19	19.5	1.8
10	11	751	791	771.0	18	18	18.0	2.3
11	1	764	817	790.5	45	48.5	46.8	5.9
11	2	1645	1586	1615.5	49	48	48.5	3.0
11	3	2505.5	2689.5	2597.5	49	51	50.0	1.9
11	11	1992.5	2043	2017.8	47	42.5	44.8	2.2
11	18	819	821	820.0	55	52	53.5	6.5
11	19	570.5	612	591.3	63	59	61.0	10.3
11	21	594.5	603	598.8	66	60	63.0	10.5
12	2	565	398	481.5	3	3	3.0	0.6
12	3	815	1007	911.0	2	2	2.0	0.2
12	11	565	683	624.0	3	2	2.5	0.4
13	2	543	504	523.5	30	26	28.0	5.3
13	3	792.5	971	881.8	36	32	34.0	3.9
13	11	567	560	563.5	29	22.5	25.8	4.6
14	2	640	576.5	608.3	15.5	17	16.3	2.7
14	3	807	983	895.0	22	16.5	19.3	2.2
14	11	760.5	763	761.8	18.5	14	16.3	2.1
15	2	852	786	819.0	5	3	4.0	0.5
15	3	1461	1981	1721.0	6	6	6.0	0.3
15	11	1179	1170	1174.5	3	3	3.0	0.3
18	2	3379.5	3115.5	3247.5	68	53	60.5	1.9
18	3	4512.5	4785.5	4649.0	73	64	68.5	1.5
18	4	989	1201	1095.0	57.5	55	56.3	5.1
18	6	501	486	493.5	47	52	49.5	10.0
18	8	1109	995	1052.0	64	53	58.5	5.6
18	11	4319.5	4335	4327.3	50.5	49.5	50.0	1.2
18	12	554	560	557.0	58	60	59.0	10.6
18	15	1765.5	1875.5	1820.5	88	86	87.0	4.8
18	18	549	552.5	550.8	83.5	69	76.3	13.8
18	24	569	526	547.5	84	65.5	74.8	13.7
18	26	1601	1530.5	1565.8	76	65	70.5	4.5
19	2	1178	1191	1184.5	8	10	9.0	0.8
19	3	1653	1903	1778.0	8	8	8.0	0.4
19	11	1585	1605	1595.0	10	5	7.5	0.5
21	2	4829	4971.5	4900.3	40	44	42.0	0.9
21	3	7613	7726	7669.5	71	37	54.0	0.7
21	4	1749	1860	1804.5	24	31	27.5	1.5
21	5	809	782.5	795.8	34	26	30.0	3.8
21	6	1377	1321	1349.0	25	43	34.0	2.5
21	7	579	573	576.0	34.5	21.5	28.0	4.9
21	8	1870.5	2095.5	1983.0	52	40	46.0	2.3
21	9	820	852.5	836.3	21	37	29.0	3.5
21	10	716	773.5	744.8	56	32	44.0	5.9
21	11	8053	8187	8120.0	27	28.5	27.8	0.3

21	12	1430	1547	1488.5	29	44	36.5	2.5
21	14	660	746	703.0	38	26	32.0	4.6
21	15	3845	3620	3732.5	64.5	33	48.8	1.3
21	16	947	916	931.5	30.5	41	35.8	3.8
21	18	648.5	681	664.8	39.5	28	33.8	5.1
21	24	1215	1168	1191.5	39	38.5	38.8	3.3
21	25	400.5	525	462.8	33	23	28.0	6.1
21	26	4071	3726	3898.5	48.5	43	45.8	1.2
20	2	3793	3792	3792.5	3	3	3.0	0.1
20	3	5017	5730	5373.5	2	2	2.0	0.0
20	4	1072.5	1205	1138.8	1	1	1.0	0.1
20	6	634	577	605.5	2	1	1.5	0.2
20	8	1288	1278.5	1283.3	2	1	1.5	0.1
20	11	5683	5555	5619.0	3	3	3.0	0.1
20	12	638.5	617	627.8	2	3	2.5	0.4
20	15	2332	2251	2291.5	2	2	2.0	0.1
20	24	611	582	596.5	1	1	1.0	0.2
20	26	1967.5	1833	1900.3	1	3	2.0	0.1
24	1	766	783.5	774.8	1	2	1.5	0.2
24	2	2378.5	2101	2239.8	2	2	2.0	0.1
24	3	3134.5	3656	3395.3	2	2	2.0	0.1
24	4	545	657	601.0	2	2	2.0	0.3
24	8	845	894	869.5	2	2	2.0	0.2
24	11	3319	3220	3269.5	3	3	3.0	0.1
24	15	780	741	760.5	2	3	2.5	0.3
24	18	845	865	855.0	2	3	2.5	0.3
24	20	705	660	682.5	2	2	2.0	0.3
24	21	499.5	505.5	502.5	2	2	2.0	0.4
24	26	798	662	730.0	4	3	3.5	0.5
26	3	501	597	549.0	3	2	2.5	0.5
