# Supplementary material for: Llama Single Domain Antibodies Specific for the 7 Botulinum Neurotoxin Serotypes as Heptaplex Immunoreagents
Source: PLoS One. 2010 Jan 21;5(1):e8818. doi: 10.1371/journal.pone.0008818 (PMC2809108; doi:10.1371/journal.pone.0008818)
Supplement: Figure S1 — Primary structures of anti-BoNT sdAb clones. Predicted amino acid sequences of sdAb identified as positive by monoclonal phage ELISA on each serotype of toxin: a) A toxin; b) B toxin c) C toxin d) D toxin, e) E toxin, f) F toxin, g) G toxin. (0.13 MB PDF) [file pone.0008818.s002.pdf]

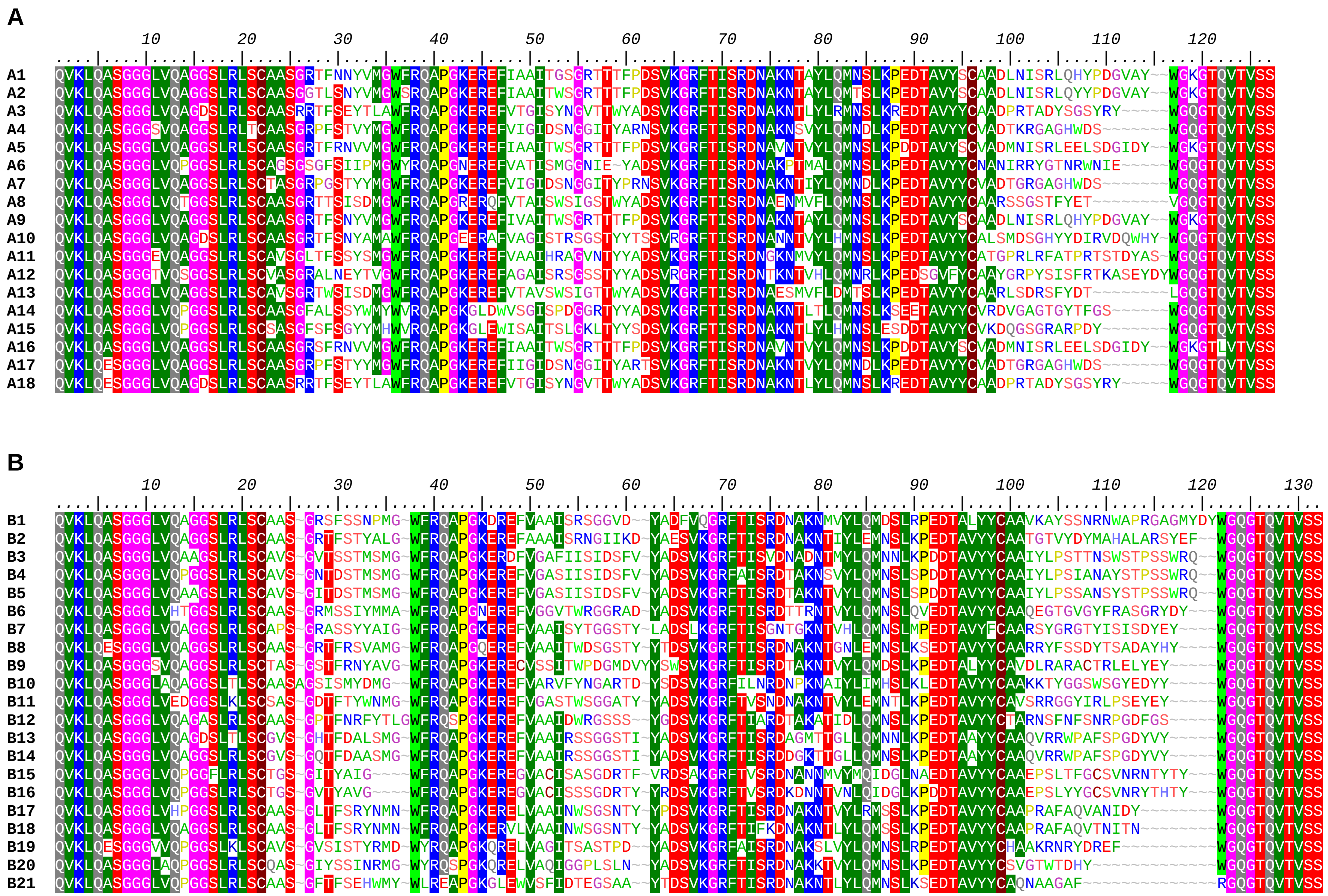
A
A1 QVKLQASGGGLVQAGGSLRLSCAASGRTFNNYVMGWFRQAPGKEREFIAAITGSGRTTTFPDSVKGRFTISRDNAKNTAYLQMNSLKPEDTAVYSCAADLNISRLQHYPDGVAY~~WGKGTQVTVSS
A2 QVKLQASGGGLVQAGGSLRLSCAASGGTLSNYVMGWSRQAPGKEREFIAAITWSGRTTTFPDSVKGRFTISRDNAKNTAYLQMTSLKPEDTAVYSCAADLNISRLQYYPDGVAY~~WGKGTQVTVSS
A3 QVKLQASGGGLVQAGDSLRLSCAASRRTFSEYTLAWFRQAPGKEREFVTGISYNGVTTWYADSVKGRFTISRDNAKNTLYLRMNSLKREDTAVYYCAADPRTADYSGSYRY~~~~~WGQGTQVTVSS
A4 QVKLQASGGGSVQAGGSLRLTCAASGRPFSTVYMGWFRQAPGKEREFVIGIDSNGGITYARNSVKGRFTISRDNAKNSVYLQMNDLKPEDTAVYYCVADTKRGAGHWDS~~~~~~~WGQGTQVTVSS
A5 QVKLQASGGGLVQAGGSLRLSCAASGRTFRNVVMGWFRQAPGKEREFIAAITWSGRTTTFPDSVKGRFTISRDNAVNTVYLQMNSLKPDDTAVYSCVADMNISRLEELSDGIDY~~WGKGTQVTVSS
A6 QVKLQASGGGLVQPGGSLRLSCAGSGSGFSIIPMGWYRQAPGNEREFVATISMGGNIE~YADSVKGRFTISRDNAKPTMALQMNSLKPEDTAVYYCNANIRRYGTNRWNIE~~~~~WGQGTQVTVSS
A7 QVKLQASGGGLVQAGGSLRLSCTASGRPGSTYYMGWFRQAPGKEREFVIGIDSNGGITYPRNSVKGRFTISRDNAKNTIYLQMNDLKPEDTAVYYCVADTGRGAGHWDS~~~~~~~WGQGTQVTVSS
A8 QVKLQASGGGLVQTGGSLRLSCAASGRTTSISDMGWFRQAPGRERQFVTAISWSIGSTWYADSVKGRFTISRDNAENMVFLQMNSLKPEDTAVYYCAARSSGSTFYET~~~~~~~~VGQGTQVTVSS
A9 QVKLQASGGGLVQAGGSLRLSCAASGRTFSNYVMGWFRQAPGKEREFIVAITWSGRTTTFPDSVKGRFTISRDNAKNTAYLQMNSLKPEDTAVYSCAADLNISRLQHYPDGVAY~~WGKGTQVTVSS
A10 QVKLQASGGGLVQAGDSLRLSCAASGRTFSNYAMAWFRQAPGEERAFVAGISTRSGSTYYTSSVRGRFTISRDNANNTVYLHMNSLKPEDTAVYYCALSMDSGHYYDIRVDQWHY~WGQGTQVTVSS
A11 QVKLQASGGGEVQAGGSLRLSCAVSGLTFSSYSMGWFRQAPGKEREFVAAIHRAGVNTYYADSVKGRFTISRDNGKNMVYLQMNSLKPEDTAVYYCATGPRLRFATPRTSTDYAS~WGQGTQVTVSS
A12 QVKLQASGGGTVQSGGSLRLSCVASGRALNEYTVGWFRQAPGKEREFAGAISRSGSSTYYADSVRGRFTISRDNTKNTVHLQMNRLKPEDSGVFYCAAYGRPYSISFRTKASEYDYWGQGTQVTVSS
A13 QVKLQASGGGLVQAGGSLRLSCAVSGRTWSISDMGWFRQAPGKEREFVTAVSWSIGTTWYADSVKGRFTISRDNAESMVFLDMTSLKPEDTAVYYCAARLSDRSFYDT~~~~~~~~LGQGTQVTVSS
A14 QVKLQASGGGLVQPGGSLRLSCAASGFALSSYWMYWVRQAPGKGLDWVSGISPDGGRTYYADSVKGRFTISRDNAKNTLTLQMNSLKSEETAVYYCVRDVGAGTGYTFGS~~~~~~WGQGTQVTVSS
A15 QVKLQASGGGLVQPGGSLRLSCSASGFSFSGYYMHWVRQAPGKGLEWISAITSLGKLTYYSDSVKGRFTISRDNAKNTLYLHMNSLESDDTAVYYCVKDQGSGRARPDY~~~~~~~WGQGTQVTVSS
A16 QVKLQASGGGLVQAGGSLRLSCAASGRSFRNVVMGWFRQAPGKEREFIAAITWSGRTTTFPDSVKGRFTISRDNAVNTVYLQMNSLKPDDTAVYSCVADMNISRLEELSDGIDY~~WGKGTLVTVSS
A17 QVKLQESGGGLVQAGGSLRLSCAASGRPFSTYYMGWFRQAPGKEREFIIGIDSNGGITYARTSVKGRFTISRDNAKNTVYLQMNDLKPEDTAVYYCVADTGRGAGHWDS~~~~~~~WGQGTQVTVSS
A18 QVKLQESGGGLVQAGDSLRLSCAASRRTFSEYTLAWFRQAPGKEREFVTGISYNGVTTWYADSVKGRFTISRDNAKNTLYLQMNSLKREDTAVYYCAADPRTADYSGSYRY~~~~~WGQGTQVTVSS
B
B1 QVKLQASGGGLVQAGGSLRLSCAAS~GRSFSSNPMG~WFRQAPGKDREFVAAISRSGGVD~~YADFVQGRFTISRDNAKNMVYLQMDSLRPEDTALYYCAAVKAYSSNRNWAPRGAGMYDYWGQGTQVTVSS
B2 QVKLQASGGGLVQAGGSLRLSCAAS~GRTFSTYALG~WFRQAPGKEREFAAAISRNGIIKD~YAESVKGRFTISRDNAKNTIYLEMNSLKPEDTAVYYCAATGTVYDYMAHALARSYEF~~WGQGTQVTVSS
B3 QVKLQASGGGLVQAAGSLRLSCAVS~GVTSSTMSMG~WFRQAPGKERDFVGAFIISIDSFV~YADSVKGRFTISVDNADNTMYLQMNNLKPDDTAVYYCAAIYLPSTTNSWSTPSSWRQ~~WGQGTQVTVSS
B4 QVKLQASGGGLVQPGGSLRLSCAVS~GNTDSTMSMG~WFRQAPGKEREFVGASIISIDSFV~YADSVKGRFAISRDTAKNSVYLQMNSLSPDDTAVYYCAAIYLPSIANAYSTPSSWRQ~~WGQGTQVTVSS
B5 QVKLQASGGGLVQAAGSLRLSCAVS~GITDSTMSMG~WFRQAPGKEREFVGASIISIDSFV~YADSVKGRFTISRDTAKNTVYLQMNSLSPDDTAVYYCAAIYLPSSANSYSTPSSWRQ~~WGQGTQVTVSS
B6 QVKLQASGGGLVHTGGSLRLSCAAS~GRMSSIYMMA~WFRQAPGNEREFVGGVTWRGGRAD~YADSVKGRFTISRDTTRNTVYLQMNSLQVEDTAVYYCAAQEGTGVGYFRASGRYDY~~~WGQGTQVTVSS
B7 QVKLQASGGGLVQAGGSLRLSCAPS~GRASSYYAIG~WFRQAPGKEREFVAAISYTGGSTY~LADSLKGRFTISGNTGKNTVHLQMNSLMPEDTAVYFCAARSYGRGTYISISDYEY~~~~WGQGTQVTVSS
B8 QVKLQESGGGLVQAGGSLRLSCAAS~GRTFRSVAMG~WFRQAPGQEREFVAAITWDSGSTY~YTDSVKGRFTISRDNAKNTGNLEMNSLKSEDTAVYYCAARRYFSSDYTSADAYHY~~~~WGQGTQVTVSS
B9 QVKLQASGGGSVQAGGSLRLSCTAS~GSTFRNYAVG~WFRQAPGKERECVSSITWPDGMDVYYSWSVKGRFTISRDTAKNTVYLQMDSLKPEDTALYYCAVDLRARACTRLELYEY~~~~~WGQGTQVTVSS
B10 QVKLQASGGGLAQAGGSLTLSCAASAGSISMYDMG~~WFRQAPGKEREFVARVFYNGARTD~YSDSVKGRFILNRDNPKNAIYLIMHSLKLEDTAVYYCAAKKTYGGSWSGYEDYY~~~~~WGQGTQVTVSS
B11 QVKLQASGGGLVEDGGSLKLSCSAS~GDTFTYWNMG~WFRQAPGKEREFVGASTWSGGATY~YADSVKGRFTVSNDNAKNTVYLEMNTLKPEDTAVYYCAVSRRGGYIRLPSEYEY~~~~~WGQGTQVTVSS
B12 QVKLQASGGGLVQAGASLRLSCAAS~GPTFNRFYTLGWFRQSPGKEREFVAAIDWRGSSS~~YGDSVKGRFTIARDTAKATIDLQMNSLKPEDTAVYYCTARNSFNFSNRPGDFGS~~~~~WGQGTQVTVSS
B13 QVKLQASGGGLVQAGDSLTLSCGVS~GHTFDALSMG~WFRQAPGKEREFVAAIRSSGGSTI~YADSVKGRFTISRDAGMTTGLLQMNNLKPEDTAAYYCAAQVRRWPAFSPGDYVY~~~~~WGQGTQVTVSS
B14 QVKLQASGGGLVQAGGSLRLSCGVS~GQTFDAASMG~WFRQAPGKEREFVAAIRSSGGSTI~YADSVKGRFTISRDDGKTTGLLQMNSLKPEDTAAYYCAAQVRRWPAFSPGDYVY~~~~~WGQGTQVTVSS
B15 QVKLQASGGGLVQPGGFLRLSCTGS~GITYAIG~~~~WFRQAPGKEREGVACISASGDRTF~VRDSAKGRFTVSRDNANNMVYMQIDGLNAEDTAVYYCAAEPSLTFGCSVNRNTYTY~~~WGQGTQVTVSS
B16 QVKLQASGGGLVQPGGSLRLSCTGS~GVTYAVG~~~~WFRQAPGKEREGVACISSSGDRTY~YRDSVKGRFTVSRDKDNNTVNLQIDGLKPDDTAVYYCAAEPSLYYGCSVNRYTHTY~~~WGQGTQVTVSS
B17 QVKLQASGGGLVHPGGSLRLSCAAS~GLTFSRYNMN~WFRQAPGKERELVAAINWSGSNTY~YPDSVKGRFTISRDNAKNTVYLRMSSLKPEDTAVYYCAAPRAFAQVANIDY~~~~~~~~WGQGTQVTVSS
B18 QVKLQASGGGLVQAGGSLRLSCAAS~GLTFSRYNMN~WFRQAPGKERVLVAAINWSGSNTY~YADSVKGRFTIFKDNAKNTLYLQMSSLKPEDTAVYYCAAPRAFAQVTNITN~~~~~~~~WGQGTQVTVSS
B19 QVKLQESGGGVVQPGGSLKLSCAVS~GVSISTYRMD~WYRQAPGKQRELVAGITSASTPD~~YADSVKGRFAISRDNAKSLVYLQMNSLRPEDTAVYYCHAAKRNRYDREF~~~~~~~~~~WGQGTQVTVSS
B20 QVKLQASGGGLAQPGGSLRLSCQAS~GIYSSINRMG~WYRQSPGKQRELVAQIGGPLSLN~~YADSVKGRFTISRDNAKKTVYLQMNSLKPEDTAVYYCSVGTWTDHY~~~~~~~~~~~~~WGQGTQVTVSS
B21 QVKLQASGGGLVQPGGSLRLSCAAS~GFTFSEHWMY~WLREAPGKGLEWVSFIDTEGSAA~~YTDSVKGRFTISRDNAKNTLYLQMNSLKSEDTAVYYCAQNAAGAF~~~~~~~~~~~~~~RGQGTQVTVSS

C

```
             10        20        30        40        50        60        70        80        90        100       110       120       130
     ....|....|....|....|....|....|....|....|....|....|....|....|....|....|....|....|....|....|....|....|....|....|....|....|....|....|....
C1   QVKLQASGGGLVQGGGSLRLSCAASGRTFSTYGVG~~WFRQAPGKE~REFVAGISWNGDSTYYAPSVKGRFTISRDNTKNTAYLQMNSLKPEDTAVYYC~~~~~~~AVGTISYSLRYAAPASYDY~~~WGQGTQVTVSS
C2   QVKLQASGGGLVQPGGSLRLSCVGSGRTFTNSRMG~~WFRQAPGKE~REFVSVVSWSGETTYYSDSVKGRFTISRDNAKNTVYLQMNSLKPEDTAVYFC~~~~~~~AADFPPLTHWHTRPSVYDY~~~WGQGTQVTVSS
C3   QVKLQASGGGLVQAGGSLRLSCGVSGHTLSGVVAG~~WIRQAPGKD~REFVAALSGSGLSTYYADSVKGRFTISRDNAKNTVYLQMDSLQPEDTAVYYC~~~~~~~AASPEGPVRTPASYRF~~~~~~WGQGTQVTVSS
C4   QVKLQASGGGLVQAGGSLRLSCAASGRTFSSYVMG~~WFRQAPEKE~REFVAAANRDGTSTYHADSVRGRFTISRDDAKNTVYLQMNSMKPEDAAVYYC~~~~~~~AAAEDPYHLSPYFYRKEDKYTYWGQGTQVTVSS
C5   QVKLQASGGGLVQAGGSLRLSCAASLSTASDFVAG~~WFRQAPGKD~REFVAALSGSGLSTYYGDSVKGRFTISRDNAKKMVYLQMDDLKPEDTAVYYC~~~~~~~AATPEGPVRSPTAYRY~~~~~~WGQGTQVTVSS
C6   QVKLQASGGGLVQPGGSLRLSCAASGRAISAYIMA~~WFRQVPGKE~REFVAGTHWNARSTYYDDSAEGRFTISRDNAKNTVYLEMNSLKPEDTAVYFC~~~~~~~AQDQSPYGTSLSLRGSKY~~~~WGQGTQVTVSS
C7   QVKLQASGGGLVQTGDSLRLSCAASELTFSDYVMG~~WFRQAPGKE~REFVAAINRSGVSTRHVDSVRGRFTISRDNAKNTLFLQMNSLKAEDTAVYYC~~~~~~~AAGRNMWTENIWTYSSPEY~~~WGQGTQVTVSS
C8   QVKLQASGGGLVQPGGSLRLSCAASGRGFNTYSIG~~WFRQAPGKD~REFVAAISGGGGATNYADPVKGRFIIARDNAKNTVYLQMNNLKPEDTAVYYC~~~~~~~AASQLYIRPDYDDPIPY~~~~~WGQGTQVTVSS
C9   QVKLQASGGGSVQAGGSLRLSCAVFGHTLNGVVSG~~WFRQAPGKD~REFVAALSGSGISTYYADSEKGRFTISRDIAKQTVYLQMDSLKPEDTAVYFC~~~~~~~AASPEGAVRSPSSYRF~~~~~~WGQGTQVTVSS
C10  QVKLQESGGGAVQAGVSLRLSCAASGRAISTYNMA~~WFRQVPGKE~REFLAGSHWNGRSTYYDDSAKGRFTISRDNAKNTVYLQMNALMPGDTAVYFC~~~~~~~AQDQSPYGTSLSLRGSKY~~~~WGQGTQVTVSS
C11  QVKLQASGGGLVQAGGSLSLACTASGRTSNPLNMG~~WFRQAPEKE~REFVSSISASGTTAGYAVSVKGRFTISRDDAANTVYLQMNSLKPEDTAVYYC~~~~~~~AADPASYYSNARYSKAVEYRY~WGQGTQVTVSS
C12  QVKLQASGGGLVQPGGSLRLSCVGSGSVLSQNAMA~~WYRQAPGKQ~REVVAVIGSRGTISYA~DSVKGRFIISRDNAKNMVDLQMNSLKPEDTAVYFC~~~~~~~HTRGPNTNWVVY~~~~~~~~~~WGQGTQVTVSS
C13  QVKLQASGGGLVQAGGSLRLSCAASGRPLRLYQVG~~WFRQAPEKE~REFVGRFLWNQNYTIYSRSLPGRFTISRDNAENTVYLQMNNLQPEDTAVYFC~~~~~~~AADITGGGHNYAY~~~~~~~~~WGQGTQVTVSS
C14  QVKLQASGGGLVQPGGSLRLSCVGSGGVLSQNAMA~~WYRQAPGKQ~REVVAVIGSRGTISYA~DSVKGRFIISRDNAKNMVDLQMNSLKPEDTAVYFC~~~~~~~HTRGPNTNWVVY~~~~~~~~~~WGQGTQVTVSS
C15  QVKLQASGGGLVQPGGSLRLSCVGSGSEFSLNAMG~~WYRQAPGKQ~REVVAIIGARGTTSYA~DSVRGRFIISRDNAKNMVYLQMNSLNVEDTAVYLC~~~~~~~NTRGPNTNWTTY~~~~~~~~~~WGQGTQVTVSS
C16  QVKLQASGGGLVQPGASLRLSCAASVRTLGNYVMG~~WFRQAPGKE~REFVTAMTGTGEWIWS~DSVKGRFTPSRDNAKNTVYLQMNSLKPEDTALYYC~~~~~~~AARTGTVLTRTASDYSF~~~~~WGQGTQVTVSS
C17  QVKLQASGGGLVQAGGSLRLSCTASGLISSAYGMG~~WFRQAPGKE~REFVGTITWRNSRYYG~DSVKGRFTVSRGNARNMVILQMDSLKPEDTAVYYC~~~~~~~AVRTRGPANYGSDYKRSVEYDFWGPGTQVTVSS
C18  QVKLQASGGGLVQPGGSLRLSCAGDGSIFRDSSIG~~WYRQAPGEQ~REFVAIIASGGSTNYA~DSAKGRFTISRDNAKNTVYLQMNSLKPEDTGVYYC~~~~~~~NLKRYSFGEKNY~~~~~~~~~~WGQGTQVTVSS
C19  QVKLQASGGGLVQPGGSLRLSCVGSGSLFSVSSVG~~WYRQAAGKQ~REFVAVITSGSSTNYA~DSVKGRFTISRDNAKNTVDLQMTSLKPEDTAVYYC~~~~~~~NLKRAPHYHPSDY~~~~~~~~~WGRGTLVTVSS
C20  QVKLQASGGGLAQAGGSLRLSCKASGRAFSNLNMA~~WFRQTPDNDEREFVAAIRWVGTTYYA~DSVKGRFTISRDSAENTMNLQMNNLKPEDTAVYYC~~~~~~~SAHDRSNSANVYYPQNYGY~~~WGQGTQVTVSS
C21  QVKLQASGGGLVQPGGSLRLSCAASGSIFNTYSIG~~WYRQGPGKQ~REYIAVIGVAGSTNYG~DSMKGRITISRDNDKNMVYLQMNSLKPEDTAVYYC~~~~~~~KAFRRDGDY~~~~~~~~~~~~~WGQGTQVTVSS
C22  QVKLQASGGGLVQPGGSLRLSCTVSGHAFSDINLG~~WFRSKTGEE~RAFVAAISTSGGTWYA~DSVKGRFTISRDSAKNAVYLQMNSLKAEDTAVYYC~~~~~~~AVLREGGIYSSSSYAY~~~~~~WGQGTQVTVSS
C23  QVKLQASGGGLAQAGGSLRLSCAASARSTRAYDMA~~WFRQAPGKE~REFVASITWGSSSTDYSDSVKGRFAITSDAGIFSGGSAKKTAYLQMNSLKTEDTAVYYCAATFDRRWTDTGRAPYPY~~~~WGQGTQVTVSS
C24  QVKLQASGGGLVQAGGSLTLSCADSGHTFSIMA~~~~WFRQAPGKE~REFVASRTWRNWTYYP~DSVKGRFTISRDSAKNTLYLQMNSLKPEDTAVYYC~~~~~~~AARRMDSGSYRYNDRETYDY~~WGQGTQVTVSS
C25  QVKLQASGGGLVQAGGSLRLSCVASGPMGTFSNYPMAWFRQASGKD~REFVGAITGSGGSTWYADSVKGRFTISRDNAKNTVYLQMNSLEPEDTAVYYC~~~~~~~ATGAEGSIRLPSNYNT~~~~~~WGQGTQVTVSS
C26  QVKLQASGGGLVQAGDSLKLSCAASGRTYTMG~~~~~WFRQAPGKE~REFVAAILWSGENIGNEDWVKGRFTISRDVAKNTVFLEMNSLKPEDTAVYYC~~~~~~~AATAHGALSSRQNSYDY~~~~~WGQGTQVTVSS
```

D

```
             10        20        30        40        50        60        70        80        90        100       110       120       130
     ....|....|....|....|....|....|....|....|....|....|....|....|....|....|....|....|....|....|....|....|....|....|....|....|....|....|...
D1   QVKLQASGGGLVQPGGSLRLSCAASGGSFSTLNMAWFRQAPGNEREVII~~AISGNEDYGYYADSVKGRFTISKDSAKTTVYLQMNNLKPEDTAVYYCAATRRARNWLGAPFDY~~~~~~~~WGQGTQVTVSS
D2   QVKLQASGGGVVQAGGSLRLSCAASGRTVTNYAMGWFRQAPGKEREFLA~~RIRWRGTDTYYADSVKGRFTVSRDNAKNTVYLQMNSLKPEDTAVYYCAVHTSSGSFSDTKTYDY~~~~~~~WGQGTQVTVSS
D3   QVKLQASGGGSVQAGDSLRLSCAASGGSFSTLNMAWFRQAPGNEREVII~~AISGNEDYGYYADSVKGRFTISKDSAKTTVYLQMNNLKPEDTAVYYCAATRRARNWLGAPFDY~~~~~~~~WGQGTQVTVSS
D4   QVKLQASGGGLVQPGGSLRLSCAASGFTFSSYWMYWVRQAPGKGLEWVSI~INTGGGNTY~YADSVKGRFTISRDNAKNTLYLQMNSLKSEDTAVYYCARDPTLGSWQYDY~~~~~~~~~~~WGQGTQVTVSS
D5   QVKLQASGGGLVQPGGSLRLSCAASGFIFSSYSMSWVRQAPGKGPERVSS~ISNGGGYTY~YTDSVKGRFTISRDNAKNTLYLQMNSLKPEDTALYYCTKRRNPGSAMGSFPGDYES~~~~~RGQGTQVTVSS
D6   QVKLQASGGGVVQAGGSLRLSCTASGRTVTNYAMGWFRQAPGKEREFLAR~IRWRGTDTY~YADSVKGRFTVSRDNAKDTVYLQMNSLKPEDTAVYYCAVHTSSGSFSDTKTYDY~~~~~~~WGQGTQVTVSS
D7   QVKLQASGGGLVQAGGSLRLSCAASGRTFSGYGMGWFRQAPGQERDFVAA~IESSGGGAE~YANSVQGRFSIFRDNAKNTVYLQMNSLKPEDTAVYYCAARTVWATSIYANDYTY~~~~~~~WGQGTQVTVSS
D8   QVKLQASGGGLVQAGGSLRLSCAASELTFSDYVMGWFRQAPGKEREFVAA~INRSGVSTR~HVDSVRGRFTISRDNAKNTLFLQMNSLKAEDTAVYYCAAGRNMWTENIWTYSSPEY~~~~~WGQGTQVTVSS
D9   QVKLQASGGGLVQPGGSLRLSCLASGFTFEHYSMAWFRQAPGKDRLFVAR~ISRNGVITD~YADSLKGRFTVSRDNAKNTVYLQMNSLTPEDTAVYYCAADAQRSQTTYVGAYEY~~~~~~~WGQGTQVTVSS
D10  QVKLQQSGGGSVQAGDSLRLSCAASGGSFSTLNMAWFRQAPGNEREVIIA~ISGSEDYGY~YADSVKGRFTISKDSAKTTVYLQMNNLKPEDTAVYYCAATRRARNWLGAPFDY~~~~~~~~WGQGTQVTVSS
D11  QVKLQASGGGLVQAGGSLRLSCAASGRTFNNLHVAWFRQAPGNEREPVTA~KSWSGDYEY~YADSAKGRFTISMDSGKTTVYLQMNNLKPEDTALYYCAATTRARNWLGAPFDY~~~~~~~~WGRGTQVTVSS
D12  QVKLQASGGGLVQPGGSLRLSCAASGFTFSSSDLTWVRQAPGKGLEWVSS~IYSGGSITY~YADSVKGRFTISRDNAKNTLYLQMNSLKSEDTAVYYCARGGSYYYSPNDD~~~~~~~~~~~WGQGTQVTVSS
D13  QVKLQESGGGLVQPGGSLRVSCAASGFTFSSYYMSWVRQAPGKGLEWVSG~INTGGGSTY~YADSVKGRFTISRDNAENTLYLQMNSLKPEDTALYYCARGVPGRAWYVYDD~~~~~~~~~~WGQGTQVTVSS
D14  QVKLQASGGGLAQAGGSLTLSCAASGRSFSTYVMGWFRQAPGKEREFVAA~IRWNGGNVY~YGDSMKGRFTISRDSAKNTVYLQMNNLNSDDTAVYFCAARGPNSGTRYFYTDEKSADWFRDWGQGTQVTVSS
D15  QVKLQASGGGSVHTGDSLRLSCAASGGTFERLNMAWFRQAPGKEREVINA~VSGDGGYGY~YADSVKGRFTISKDSAKTTVYLEMNNLKPEDTAIYYCAATRRARNWLGAPFDY~~~~~~~~WGQGTQVTVSS
D16  QVKLQASGGGLAQPGGSLTLSCAASGRTLDTYVMGWFRQAPGKEREFVAA~IRWNGGNVY~YGDSPKGRFTISRDSAKNTVYLQMNSLNSEDTAVYFCAARGPNPGTRYFYTDEKSTDWFRDWGQGTQVTVSS
```

```
D17  QVKLQASGGGLVQAGGSLRLSCAASGQFYSTWAMGWFRQAPGKEREFVAA~VNWNGRVKD~YVNSVKGRFTISRDNAKNTIYLQMNSLSPEDTAAYYCATGRYPNVDTIRSMRIDY~~~~~~WGQGTQVTVSS
D18  QVKLQASGGGLVQAGGSLRLSCAASGRTFSSYVTGWFRQAPEREREFVAA~ANRNGTSTY~HADSVRGRFTISRDDAKNTVYLQMNSMKPEDAAVYYCAAAEDTYRLSPYFYRTEDKYAF~~WGQGTQVTVSS
D19  QVKLQASGGGLVQPGESLTLSCVASGFTFSSFWMYWVRQAPGKGLEWVSA~VNAGGGSQF~YPDSVKGRFTTSRDNAKNTVYLQMNSLKPEDTALYYCARGSDPTIGPLRSDY~~~~~~~~~WGQGTQVTVSS
D20  QVKLQASGGGLVQPGGSLRLSCAASGFRFSNYAMSWVRRAPGKGLEWVSG~ISDSGSLTN~YIDSVKGRFTISRDNARSTLTLQMDSLKPEDTAVYYCTKSRYSNIAVIRGRNDFGDD~~~~WGPGTQVTVSS
D21  QVKLQASGGGLVQPGGSLRLSCAASGRPLRLYQVGWFRQAPEKEREFVGR~FLWNQNYTI~YSRSLPGRFTISRDNAENTVYLQMNNLQPEDTAVYFCAADITGGGHNYAY~~~~~~~~~~~WGQGTQVTVSS
D22  QVKLQASGGGLVQPGGSLRLSCAASGNIFSDRSMGWYRQAPGKQREYVAA~IGAGGSTN~~YADFVKGRFTISRDNAKNTGYLQMNSLQPEDTAVYYCKLYSAPTTRDARREY~~~~~~~~~WGQGTQVTVSS
D23  QVKLQASGGGLAQAGSSLRLSCKASGRAFSNLNMAWFRQAPDNNEREFVAAIRWVGTTY~~YSDSVKGRFTISRDSAENTMNLQMNNLKPEDTAVYYCSAHDRSNSANVYYPQNYGY~~~~~WGQGTQVTVSS
D24  QVKLQASGGGLVQPGGSLRLSCAASGSLFSVSSMGWYRQAPGKEREYVAA~ITSGSSTN~~YADFVKGRFTISRDNAKNMVYLQMNNLKPEDTAVYYCKLIRRSGPEY~~~~~~~~~~~~~~WGQGTQVTVSS
D25  QVKLQASGGGLVPAGGSLRLSCVASGGTFSDYNMAWFRQAPDNEREFVAA~IRWIGATY~~YADSVKGRFTISRDNAVNTVNLQMNNLEPEDTAVYYCSAHHRRDSANVYYPSNYDN~~~~~WGHGTQVTVSS
D26  QVKLQESGGGLAQSGDSLRLSCKLSGRASSTQTLAWFRQAPGKEREFVAA~IDYTGRTTE~YTDSVKGRCTISRNNAKNMVFVQMINLKPEDTAVYYCAATPVLKYPLSVPIRADQYDL~~~WGQGTQVTVSS
D27  QVKLQASGGGLVQPGGSLRLSCAASETIFSTYSMGWDRQVPGKQRELVAV~ITTGSSTN~~YADSVKGRFTISRDNAKNTVYLEMHNLKPEDTAVYYCRTWVAPGRGTAY~~~~~~~~~~~~WGQGTQVTVSS
D28  QVKLQASGGGLVEPGGSLRLSCTASETIFSGNSMGWYRQAPGKERAFVAV~IGAGGSTN~~YADSVKGRFTISRDNAKNTVYLQMNDLKPEDTAVYFCTAWVNRLNR~~~~~~~~~~~~~~~WGQGTQVTVSS
D29  QVKLQASGGGLVQPGGSLRLSCAGSGSIFSDSSVGWYRQAPGKEREYVGV~IASGGSTN~~YADFVKGRFTISRDNAKNTVYLQMNSLKPEDTAVYYCKMIRRGGPDY~~~~~~~~~~~~~~WGQGTQVTVSS
D30  QVKLQASGGGMVQPGGSLRLSCTASESIFSGNAMGWYRQAPGKDRAFVAV~IGSGGSTN~~YDDSVKGRFTISRDNAKNTVYLQMNSLKPEDTAVYFCTAWVNRQNR~~~~~~~~~~~~~~~WGQGTQVTVSS
D31  QVKLQASGGGLVQPGGSLRLSCEASGSDVSMYSMGWYRQAPGNQRAFVAV~MASGGSTN~~YADSVKGRFTISRDNAKNTVYLQMNNLQPEDTGVYYCKVYRRAGDY~~~~~~~~~~~~~~~WGKGTQVTVSS
D32  QVKLQASGGGLVQPGGSLRLSCVGSGSEFSLNAMGWYRQAPGKQREVVAI~IGARGTTS~~YADSVRGRFIISRDNAKNMVYLQMNSLNVEDTAVYLCNTRGPNTNWTTY~~~~~~~~~~~~WGQGTQVTVSS
D33  QVKLQASGGGLVLPGGSLTLSCAASGTIFGISVMAWYRQAPGKQRELVAS~LTTGGTPN~~YQDSVKGRFTISRDIAKRTVTLQMHSLKAEDTAVYYCNANVRSSRSSYNYPEY~~~~~~~~WGQGTQVTVSS
D34  QVKLQASGGGLVQPGGSLRLSCVGSGSVLSQNAMAWYRQAPGKQREVVAV~IGSRGTIS~~YADSVKGRFIISRDNAKNMVDLQMNSLKPEDTAVYFCHTRGPNTNWVVY~~~~~~~~~~~~WGQGTQVTVSS
D35  QVKLQASGGGLVQPGGSLRLSCVASGNIFSSRPWAWFRQAPGKQREFVAL~ITGPGTRT~~YAPFAKGRFTISRDNARNTVFLQMNNLKPEDTAVYYCKDYQSDY~~~~~~~~~~~~~~~~~WGQGTQVTVSS
D36  QVKLQASGGGLVQAGGSLKLSCTASGRTYTMG~~~WFRQAPGKEREFVAA~ILWSGENIG~NEDWVKGRFTISRDVAKNAVFLEMNSLKPEDTAVYYCAATASGALSSRQNSYDY~~~~~~~WDQGTQVTVSS
```

E

```
              10        20        30        40        50        60        70        80        90       100       110       120
     ....|....|....|....|....|....|....|....|....|....|....|....|....|....|....|....|....|....|....|....|....|....|....|....|....|...
E1   QVKLQASGGGLVQAGGSLRLSCAGPGLTFANYHMGWFRQGPGKEREFVGAIRWSGSNTYYADSVKGRFTISRDNAKSTVYLQMTNLKP~EDTAVYYCAAELGSTVPAGLRDAGRYDYWGQGTQVTVSS
E2   QVKLQASGGGLVQAGGSLRLSCAASGLTFTNYHMGWFRQGPGKEREFVAGIRYTGSNTYYVDSVKGRFTISRDNAKNMVYLQMNSLKP~GDTAVYYCAAELGSTVPSGLRDAGRYDYWGQGTQVTVSS
E3   QVKLQASGGGLVQAGGSLRLSCAASGLTFGNYHMAWFRQGPGKEREFVAAIRWSGSNTYYADSVKGRFTISRDNAKNTVYLQMNSLKP~EDTAVYYCAAELGSTVPSGLRDAGRIDYWGQGTQVTVSS
E4   QVKLQASGGGLVQAGGSLRLSCAASGRTFSNFAMGWFRQAPGKEREFVAALSWSNGSTAYPDAMKGRFTISRDNAGNTGYLQMNTLKP~EDTAVYYCAAVERPRARIPVHSYEFTS~WGQGTQVTVSS
E5   QVKLQASGGGLVQAGGSLRLSCVASGRTFNSYIMGWFRQAPGKEREFVAAIRWIGSSTYYGDSVRGRFTISRDNARNTVYLQMNSLKP~EDTAVYYCASGSRFYPRDAAKYEY~~~~WGQGTQVTVSS
E6   QVKLQASGGGLVQAGDSLRLSCAASGRTLSIYATGWFRQAPGKEREFVSAIRSTGSDTYYANSVKGRFTIARDNAKNMVYLQMNSLKP~EDTAVYYCAAGRGIRLGVSRRANDYDY~WGQGTQVTVSS
E7   QVKLQASGGGLVQAGDSLRLSCAASGRTLVIYAIAWFRQAPGKEREFVSAVRSTGSDTYYANSVKGRFTISRDNSRNNVYLQMDSLQP~EDTAVYYCAAGRGQRLGVSRRQNDYDF~WGQGIQVTVSS
E8   QVKLQASGGGVVQAGGSLRLSCAASSRAFSQYIMAWYRQPPGKEREFLAAITWNGDSTYYADSVKGRFTISKDNAKNTAYLQMNSLIPREDTAVYHCAAAPGITTPQTMEDKRVLKFWGQGTQVTVSS
```

F

```
              10        20        30        40        50        60        70        80        90       100       110       120       130
     ....|....|....|....|....|....|....|....|....|....|....|....|....|....|....|....|....|....|....|....|....|....|....|....|....|....|.
F1   QVKLQASGGGKVQSGGSLRLSCAASEESFNRYGVGWYRQAPGKERVFIA~NIRGRTGSTHYHASVAGRFALSRDIAKKTVYLQMNNLRPEDTAMYYCAADRSGGSTYQF~~~~~~~~~~~WGQGTQVTVSS
F2   QVKLQASGGGLVQAGGSLRLSCAASGRTINMG~~~WFRQAPGKEREFVAAIRWTISSGY~YADSVKGRFTISGDDAKNTVYLQMNSLKPEDTAVYTCAASIKPSSLSIMNPLKYNY~~~~WGQGTQVTVSS
F3   QVKLQASGGGLVQAGGSLRLSCAASGRTISMG~~~WFRQAPGKEREFVSAMRWTSSNTY~DAEFVKGRFTMSGDDAKNTVYLQMNSLKSEDTAVYYCAASTIPKSFSIMNPLKYDY~~~~WGQGTQVTVSS
F4   QVKLQASGGGLVQAGGSLRLSCAASGRTFSTSAMGWFRRAPGKERAFVAGISWNGARTY~YADSVEGRFTISRDNAKNTVYLQMNSLNPEDTAVYYCAATNTGGSYYQPPDYEYEY~~~~WGQGTQVTVSS
F5   QVKLQASGGGLVQAGGSLRLSCAASGGAFSSYSMAWVRQVPGKEREFVAAISTGGAVTK~YADSVKGRFTISRDNPKNTVSLQMNSLKTEDTAVYYCAGRRGTGRTWNDGYNY~~~~~~~WGQGTQVTVSS
F6   QVKLQASGGGLVQPGASLRLSCAASGRTVSSYNMGWFRQAPGKEREFVAAISWGGARTL~YADSVKGRFTISRDNANNTVYLQLSSLKPEDTAVFYCAGDSNRARLSRRNNEYEY~~~~~WGQGTQVTVSS
F7   QVKLQASGGGLVQAGGSLRLSCAASERTFSTWTMGWFRQAPGKEREFVAHITWGGDGPY~YADSVKGRFTVSKDDAKKTVYLEMNNLKPEDTAVYFCAAARGFIPGPRGPTDFGS~~~~~WGQGTQVTVSS
F8   QVKLQASGGGLVQPGGSLRLSCAASGRSFTDYRMGWFRQAPGKEREFVAAVRSNGGLL~~HAGSVKGRFTVSRDNGKNMLFLQMTSLKPEDTGVYTCAVGSNWKGSSTSSGGYDY~~~~~WGQGTQVTVSS
F9   QVKLQESGGGSVQAGGSLRLSCAASGRAASNHAMGWFRQAPGKEREFVVLISWSGRSTY~YADSVKGRFTISRDFAQNAVNLQMNSLKPEDTGVYYCAAVRQYGTSWYQFREDTSRYDY~WGQGTQVTVSS
F10  QVKLQASGGGLVQAGGSLRLSCIASGRTFSRYAMGWFRQAPGKEREFVAGISWSGSTTY~YADSMKGRFTVSRDNAKNTVYLQMNSLKPEDTAVYYCAAERQTDTWYSPRYYSTSDEYDYWGQGTQVTVSS
```

G

```
              10        20        30        40        50        60        70        80        90        100       110       120       130
     ....|....|....|....|....|....|....|....|....|....|....|....|....|....|....|....|....|....|....|....|....|....|....|....|....|....|....|.
G1   QVKLQASGGGLVQAGGSLRLSCAASGRTFSSYAMG~WFRQAPGKEREFVATITWDGDSTA~~~~~YADSVKGRFTVSRDNAKNTVYLQIHSLKPEDTAVYYCAADRTRYRTRWWPGRSDEYTG~~WGQGTQVTVSS
G2   QVKLQASGGGLVQAGGSLRLSCAASGRTSMMNAMV~WFRQPPGKEREFVAGISWGGPSTA~~~~~YADSVKGRFTISRDSAKNTAYLQMDSLKPEDTAVYYCAYRATRVGIGSFSYTSPDSYDY~WGQGTQVTVSL
G3   QVKLQASGGGLVQAGGSLRLSCAASGRTTSSYAMG~WFRQAPGKEREFVAAHSWAGPSTS~~~~~YADSVKGRFTISRDNAENMVYLQMDSLKPEDTAVYYCTARATRVGVGSFAFSSPGSYPY~WGQGTQVVSS
G4   QVKLQASGGGLVQAGDSLRLSCAASGRTSNPYAVA~WFRQAPGEEREFVAVINWAGDTDR~~~~~YRNFEKGRFTISRDNAKNTAYLQMVSLKPEDTAVYYCAARLTRLGVGSWDSDKEDMYQY~WGLGTQVTVSS
G5   QVKLQASGGGLVQAGGSLRLSCAASGLAFSTYAIG~WFRQAPGKEQEFVAAIKSTDGASTS~~~~YADSVRGRFTISRDNAKSTVYLQMSYLKPEDSAVYYCAAPESRYVYAGAWDTANWYTY~~WGQGTQVTVSS
G6   QVKLQASGGGLVQAGGSLRLACAASGRTLSEYTLA~WFRQAPGKERESVATITWNGGSTV~~~~~YSDSVMGRFTISRDKPKSTVYLQMNSLKPEDTAVYFCAAKLSRLYCLSAGCYPY~~~~~~WGQGTQVTVSS
G7   QVKLQESGGGLVQAGDSLRLSCAWSGRTFIGSYVLAWFRRAPGKEREFVSTINWNGDLTS~~~~~YADSVKGRFTISRDNARDTVYLQMNSLKPEDTAVYYCAAGRRTGARNINDYNY~~~~~~WGQGTQVTVSS
G8   QVKLQASGGGLVQAGGSLRLACAASGFTFSSYAMG~WFRRGPGVEREFLAAITRDGFESM~~~~~YGDSVKGRFTISRENAKSTVFLQMNSLKPEDTAVYYCAASRNYMYHDMPPISEDY~~~~~RGQGTQVTVSS
G9   QVKLQASGGGLVQAGGSLRLSCTASGRSVSRYFMG~WFRQAPGKEREFVAAITWRDGITG~~~~~YADSVQGRFIVSRDNAQNTVYLQMHRLQPEDTAVYYCGAASYYEGEIRNLAPAY~~~~~~WGQGTQVTVSS
G10  QVKLQASGGGLMQAGGSLTLSCVASGRTFSRYFVG~WFRQAPGKEREFVAALSGSGSTIE~~~~~YADSVKGRFTISRDNAKKTVTLQMNNLKPEDTAVYVCAATSFYSSTLHSGAEYHY~~~~~WGQGTQVTVSS
G11  QVKLQASGGGLVQAGGSLRLSCAASGRTLSVYRMG~WFRQAPGKEREYVAAINWRGGGDTY~~~~IPASVKGRFTISRDNAENTMYLQMNSLEPEDTAVYYCAAGNRYGPTWIRDSEYNY~~~~~WGRGTQVTVSS
G12  QVKLQASGGGSVQAGGSLRLSCAASGFSYSVYGVG~WFRQAPGKEREFLATISFSGGVAD~~~~~YADSVKGRFTISRDNAKNTVYLQMNSLKPEDTAVYYCAASLGRGKYAH~~~~~~~~~~~~FGQGTQVTVSS
G13  QVKLQASGGGLVQAGGSLRLSCAASGRAFNGYSMA~WFRQAPRKEREFVARIEWSDGTTS~~~~~YSDPVKGRFTISGDRAKNTVYLQMNAMKPEDTAVYYCAQSAGRGRFDQ~~~~~~~~~~~~WGHGTQVTVSS
G14  QVKLQASGGGLVPAGDSLRLTCAASGRAFAMG~~~~WFRQAPGKEREFVAAIDRNYGLTK~~~~~YGNSVKGRFTISRDNAKNTVYLQMDSLKPEDTTVYYCAVKSDLDDWSAGVQYSQFYNY~~WGQGTQVTVSS
G15  QVKLQASGGGLVQVGGSLGLSCAASGGSFSNDYIA~WFRQAPGKEREFVAALPWSGGRDY~~~~~FGRSVKGRFSISRDIAKNTVYLQMNSLKPEDTAVYYCAVSRGTSGDINSPYFYKV~~~~~WGQGTQVTVSS
G16  QVKLQASGGGSVQAGGSLNLSCVVSGRSSGNYVVG~WFRQTFGKERDVVAAIKWTTGAPY~~~~~YVDSVKGRFTIFRYAAQMTVYLQMSSLKPEDTAVYYCAATRDRSTFININDYTNSHEYDYWGQGTQVTVSS
G17  QVKLQASGGGLAQAGGSLRLSCAASPPTSILVIMG~WFRQAPGKAREFVARLNLAGDTAD~~~~~YADSVRGRFAISRANARNQVDLLINNLQPEDTAVYYCAAGVRLGARFDV~~~~~~~~~~~WGQGTQVTVSS
G18  QVKLQASGGGLVQAGGSLKLSCTASGRTYTMG~~~~WFRQAPGKEREFVAAILWSGESIG~~~~~NEDSVKGRFTISRDVAKKTVSLEMNSLKPEDTAVYYCAATAHGALSSRSSSYDY~~~~~~WGHGTQVTVSS
G19  QVKLQASGGGLVQAGGSLKLSCTASGRTYTMG~~~~WFRQPPGKEREFVAAILWSGESIG~~~~~NEG~~~~RFTISRDVAKKTVSLEMNSLKPEDTAVYYCAATAHGALSSRSNSYDY~~~~~~WGHGTQVTVSS
G20  QVKLQASGGGLVQAGGSLRLSCVASGRTYTMA~~~~WFRQAPGKEREFVAAILWSGEWIG~~~~~NEG~~~~RFTISRDTATNTVSLEMNNLRPEDTAVYYCAATAQGALSSRSNSYDY~~~~~~WGQGTQVTVSS
G21  QVKLQASGGGLVQAGGSLRLSCAASGRTVSSYAMG~WFRQAPGKEREFVASILWSGESEL~~~~~NEDFALGRFTISRDSARNSVSLELNVLKPEDTAVYYCAASAQGVLSRRSNSYDY~~~~~~WGQGTQVTVSS
G22  QVKLQESGGGLVQTGGSLRLSCTPSERTFRWYVMG~WFRQAPGKEREFVAAILRSGESEL~~~~~NERSVLGRFTISRDTAKNTVFLQMNSLKLEDTAVYYCAASAQGALSRRSNSYDY~~~~~~WGQGTQVTVSS
G23  QVKLQASGGGLVQPGGSLRLSCAVSGFSLEGNAAG~WFRQAPGKEREGVACISQFIKGTY~~~~~YVDSVWGRFTISRDIANNAVYLQMNNLEPEDTAVYYCAVRPGYSCSSGAHHYDY~~~~~~WGQGTQVTVSS
G24  QVKLQASGGGLVQPGGSLRLSCAASGFTFGNYWMY~WVRQAPGKGLEWVSSINEDGTTGY~~~~~YRNSVKGRFAVSRDNAKNTLYLQMNSLKSEDTAVYFCAKDKTPYRSTSGQGENVEYEY~~GGQGTQVTVSS
G25  QVKLQASGGGLVQSGGSLRLSCVASESIFKGNTMG~WYRRAPGNPGKQRELIARIIDSGIAV~~~YSESVKGRFTIAKDNAGNTVSLQMDNLKPDDTAIYYCNKVPAFSFDH~~~~~~~~~~~~~WGQGTQVTVSS
G26  QVKLQASGGGLVQPGGSLRLTCVASESIVSFRAMS~WYRQAPGKQREAVATISNGGNIG~~~~~~YADSVKGRFTISRDNAKNTVYLQMNSLKPEDTGVYYCKARRDYGADH~~~~~~~~~~~~~WGNGTQVTVSS
G27  QVKLQASGGGLVQSGGSLRLSCAASASIGGIKTMG~WIRLAPGKQRVPGEQRELVAVIGGNFETHYLDSVKGRFSISREGAKNTVYLQMDSLKPEDTADYYCFAMRSAGWIPSTNDY~~~~~~~~WGQGTQVTVSS
```
