# Supplementary figures and images for: Llama Single Domain Antibodies Specific for the 7 Botulinum Neurotoxin Serotypes as Heptaplex Immunoreagents
Source: PLoS One. 2010 Jan 21;5(1):e8818. doi: 10.1371/journal.pone.0008818 (PMC2809108; doi:10.1371/journal.pone.0008818)

**A**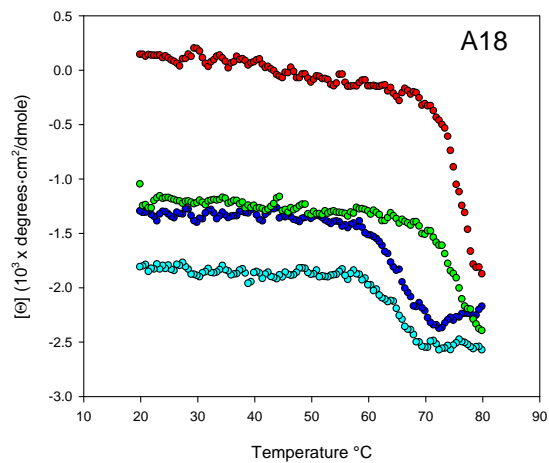**B**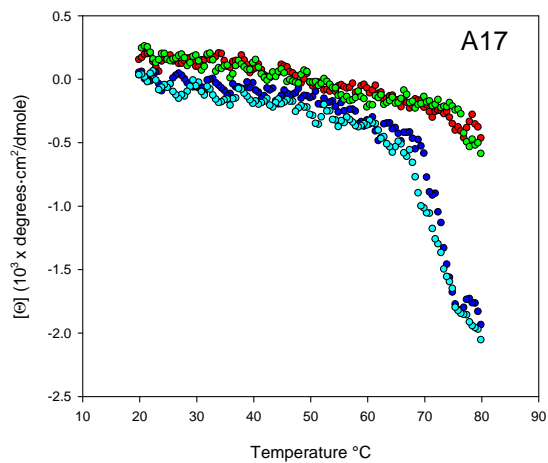**C**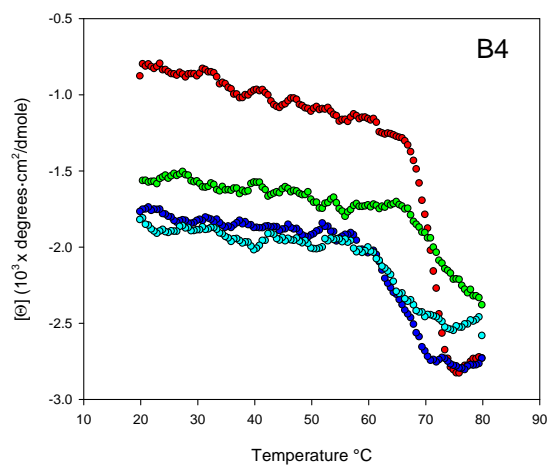**D**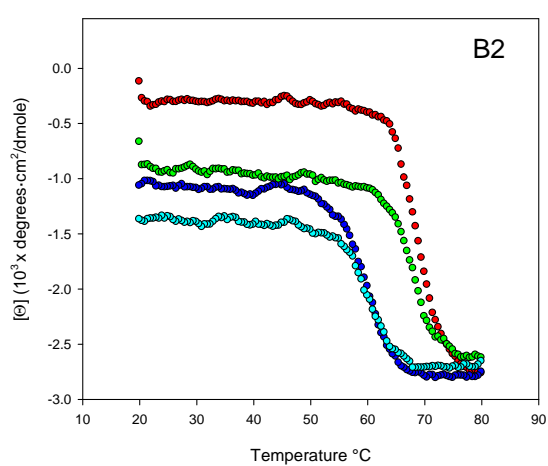**E**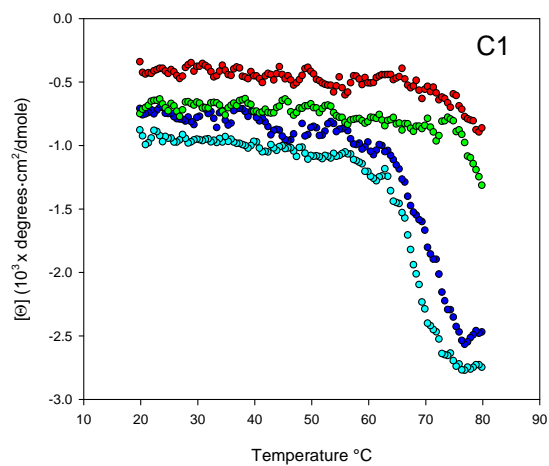**F**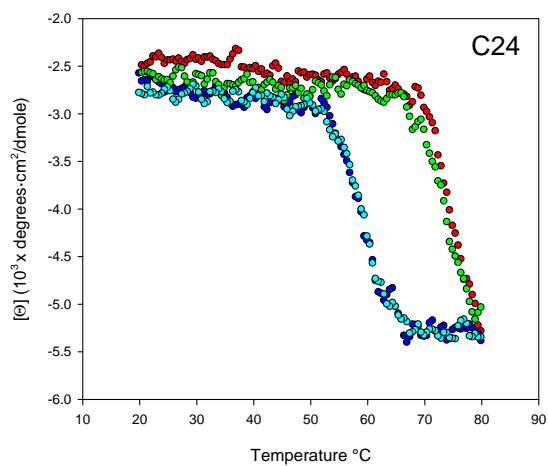

**G**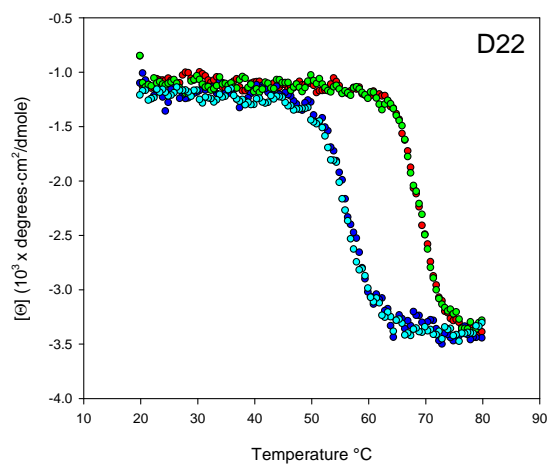**H**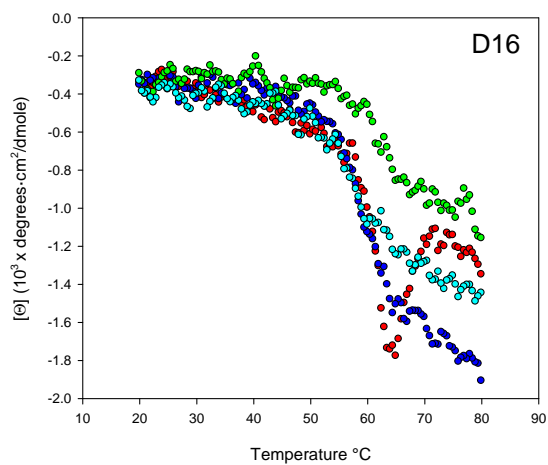**I**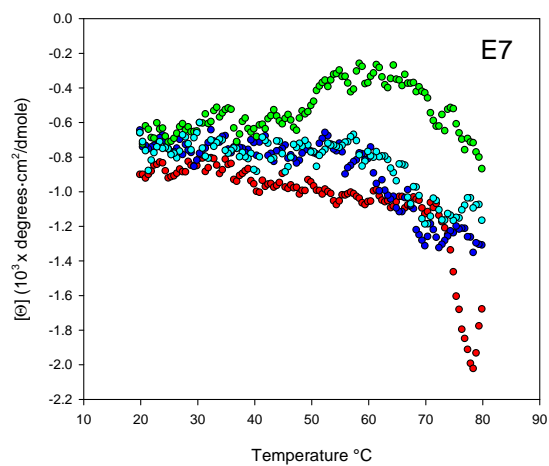**J**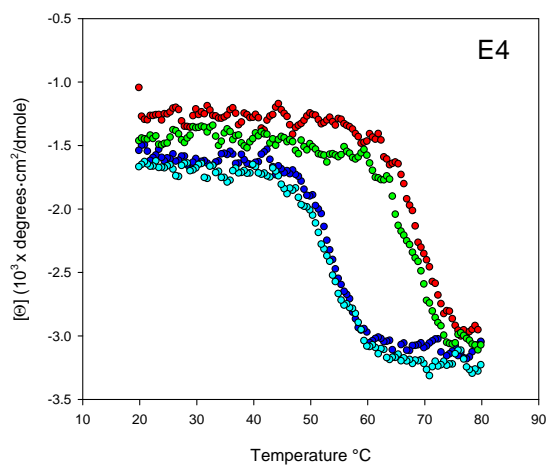**K**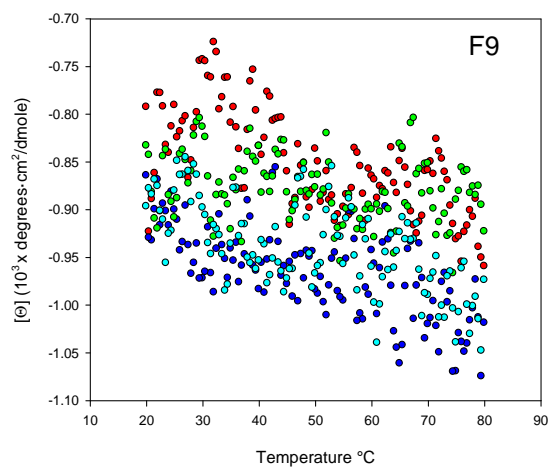**L**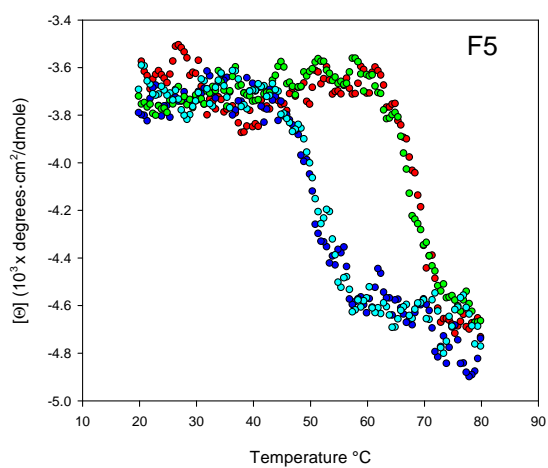

**M**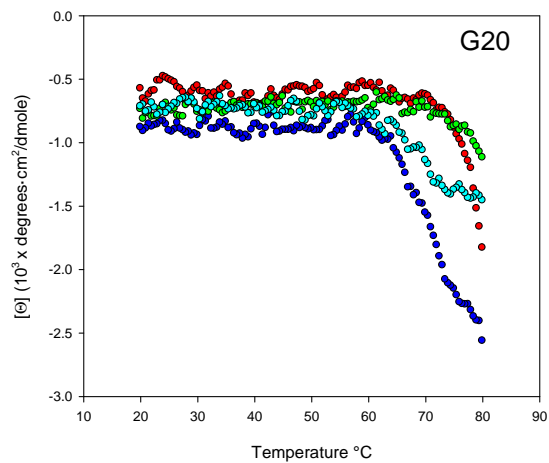**N**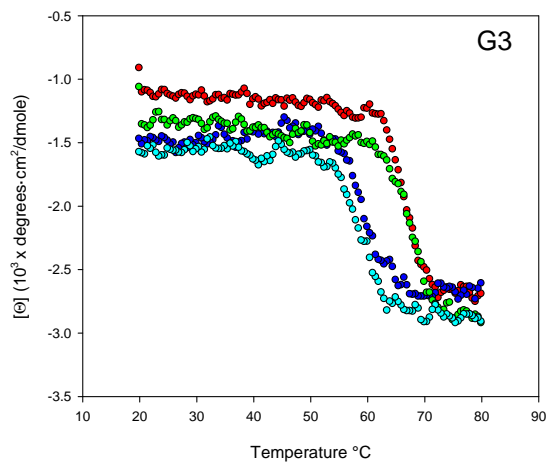**O**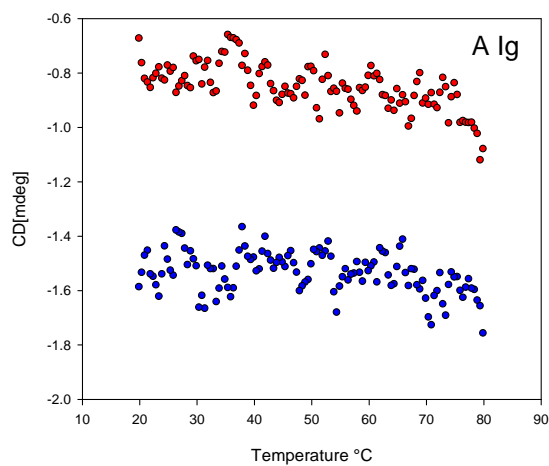**P**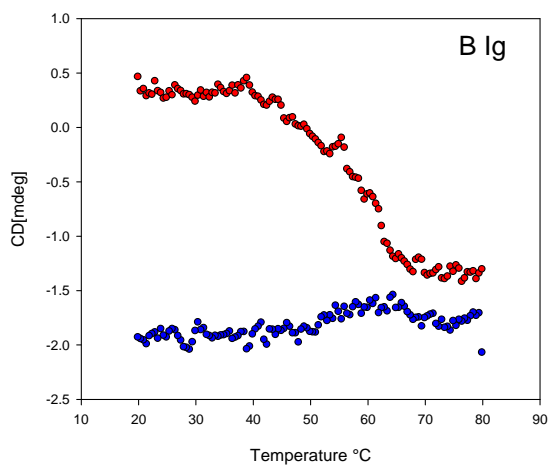**Q**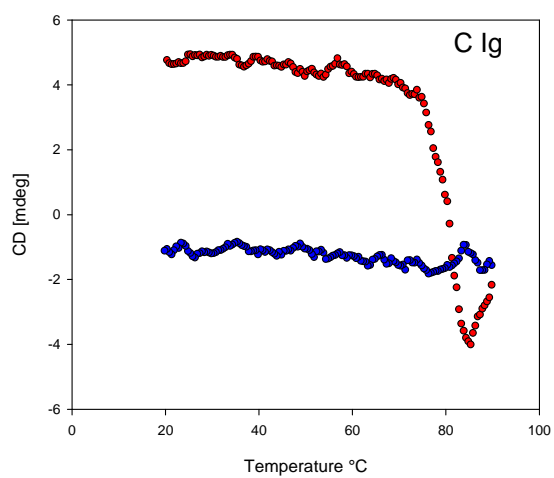**R**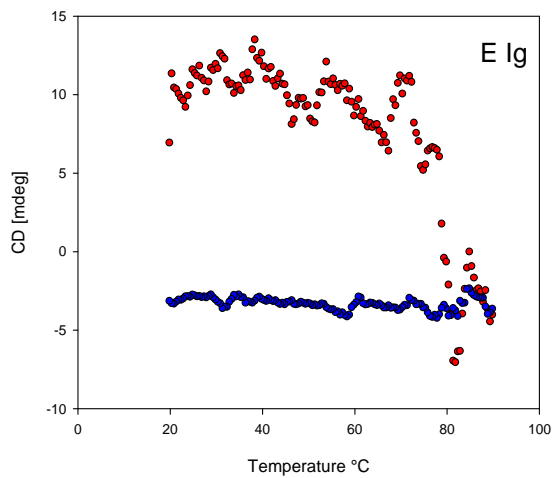

3

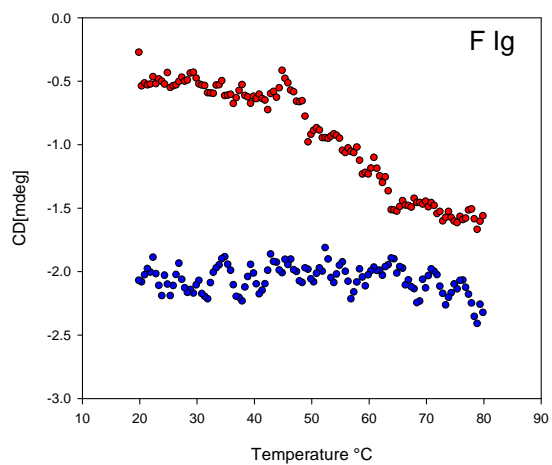

Supplement: Figure S2 — Examining the refoldabilty of sdAb in contrast to conventional immunoglobulins. Circular dichroism analysis of our final captor tracer pairs of sdAb specific for each serotype versus polyclonal immunoglobulins for those serotypes that were available: a) A18, b) A17, c) B4, d) B2, e) C1, f) C24, g) D22, h) D16, i) E7, j) E4, k) F9, l) F5, m) G20, n) G3 o) A Ig, p) B Ig, q) C Ig, r) E Ig, s) F Ig. First heating = red, first cooling = dark blue, second heating = green, second cooling = light blue. (0.15 MB PDF) [file pone.0008818.s003.pdf]
